# Bottom‐Up Space Use With Top‐Down Temporal Risk Buffering in Arid Herbivore Communities

**DOI:** 10.1002/ece3.72836

**Published:** 2026-01-04

**Authors:** John Heydinger, Uakendisa Muzuma, Tammy Hoth‐Hanssen, Genevieve Finerty, Natalia Borrego, James Beasley

**Affiliations:** ^1^ Savannah River Ecology Laboratory University of Georgia Aiken South Carolina USA; ^2^ Lion Rangers Program Kunene Namibia; ^3^ Ministry of Environment Forestry and Tourism Windhoek Namibia; ^4^ Witwatersrand University Johannesburg South Africa; ^5^ Namibian Lion Trust Kamanjab Namibia; ^6^ Max Planck Institute of Animal Behavior University of Konstanz Konstanz Germany; ^7^ Savannah River Ecology Laboratory, Warnell School of Forestry University of Georgia Aitkin South Carolina USA

**Keywords:** browsers, camera traps, diel overlap, grazers, landscape of fear, megaherbivores, Namibia, predator–prey

## Abstract

The landscape of fear (LOF) framework predicts that prey adapt their behavior to mitigate predation risk, yet the framework's expression in resource‐limited, unfenced systems remains poorly understood. Across seven large herbivore species in an unfenced arid system, space use is governed by bottom‐up constraints while predator risk is buffered in time, producing consistent low diel overlap with nocturnal carnivores and trait‐dependent moderation of spatial responses. We used camera trap data from northwest Namibia to examine how five herbivore species (gemsbok *Oryx gazella* , southern giraffe *Giraffa giraffa* , greater kudu *Tragelaphus strepsiceros* , Hartmann's mountain zebra *Equus zebra* , springbok *Antidorcas marsupialis* ) and two megaherbivores (black rhinoceros *Diceros bicornis* , African bush elephant *Loxodonta africana* ) navigate bottom‐up environmental constraints and top‐down predation risk from lions ( *Panthera leo* ) and spotted hyenas ( *Crocuta crocuta* ). We tested four hypotheses: (1) that bottom‐up environmental constraints would explain more variance in herbivore space use than predator covariates; (2) that herbivores would reduce diel overlap (Δ) with nocturnal predators rather than vacating resource patches; (3) that megaherbivores would show minimal spatiotemporal avoidance of predators, relative to smaller herbivores; and (4) that herbivores would respond reactively to recent predator presence, rather than proactively by avoiding areas of chronic predator activity. Our findings support a hierarchical LOF in which bottom‐up constraints (dry‐season progression, visibility for grazers, habitat structure for browsers) contribute to herbivore space use and top‐down predator pressures guide temporal activity: most species exhibited strongly diurnal activity, resulting in low temporal overlap with nocturnal carnivores (Δ ≈0.11–0.21 for grazers; Δ ≈0.06–0.26 for browsers). By contrast, spatial responses to predator presence were modulated by environmental context, reinforcing the importance of spatiotemporal plasticity. These results advance understandings of predator–prey dynamics, particularly in dryland ecosystems. We suggest refinements to the LOF framework for multi‐predator, resource‐constrained landscapes.

## Graphical abstract

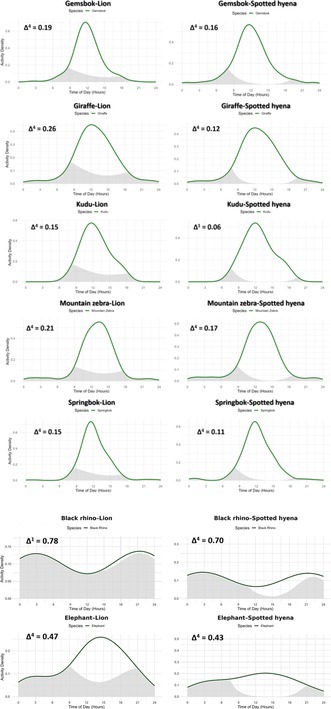

## 1 Introduction

Large herbivores must continually trade off resource acquisition against predation risk. In arid systems, this trade‐off is intensified: food and water are patchy and highly seasonal, while predator encounter risk is variable in space and time (Corp et al. 1998; Meserve et al. 2003). This dynamic has been conceptualized as a trade‐off between bottom‐up constraints (resource availability) governing herbivore space use and top‐down risks from predators (Bowyer et al. 2005), which can lead to spatial and temporal behavioral adjustments linking species' traits to local context (Riginos 2015).

The landscape of fear framework (LOF) formalizes how prey integrate perceived risk beyond actual predation rates (Laundré et al. 2001, 2014). Early work emphasized spatial avoidance of high‐risk places, but more recent studies highlight temporal structuring of risk, especially where resources cannot be abandoned without large fitness costs (Gaynor et al. 2019; Palmer et al. 2022). In such settings, prey often shift activity rather than vacate key patches, reducing diel overlap with predators that are primarily nocturnal (e.g., Tambling et al. 2015). Risk‐sensitive foraging theory further predicts that prey responses are frequently reactive to recent predator cues when proactive spatial avoidance would unduly compromise resource access (Palmer et al. 2017; Houston and Rosenström 2024; see also Schooler et al. 2024).

Critically, risk responses vary by species' traits. Body size, diet, and water dependence jointly shape vulnerability and the effects of ecological constraints. Small‐ to medium‐bodied herbivores typically are vulnerable to predation risk and thus exhibit strong spatiotemporal avoidance of predators (Skinner and Chimimba 2005; Palmer et al. 2017). By contrast, megaherbivores (e.g., black rhinoceros *Diceros bicornis* , African bush elephant *Loxodonta africana* ) are largely invulnerable to predation and are expected to tolerate predator presence near high‐value resources, modulating activity modestly, if at all (du Toit 2005; Whyte 2005). Diet and habitat structure add further constraints: browsers can experience co‐variance in resources and risk where forage also provides predator cover, whereas water‐dependent grazers are periodically forced into high‐risk areas near water (Valeix et al. 2009), especially during periods of water scarcity, e.g., dry seasons in arid areas.

We test these dynamics in an unfenced, arid ecosystem in northwest Namibia where large herbivores share space with apex predators (lions *Panthera leo* , spotted hyenas *Crocuta crocuta* ). This landscape provides a natural laboratory for assessing when bottom‐up environmental constraints, top‐down predation, and behavioral flexibility interact to shape herbivore space use and temporal activity. Using trail camera data collected from key resource sites across multiple dry seasons, we analyzed seven focal herbivore species spanning a broad trait spectrum—gemsbok ( *Oryx gazella* , mixed feeder), southern giraffe ( *Giraffa giraffa* , browser), greater kudu ( *Tragelaphus strepsiceros* , browser), Hartmann's mountain zebra ( *Equus zebra* , grazer), springbok ( *Antidorcas marsupialis* , grazer), and two megaherbivores, black rhino and elephant. These species are locally preyed upon by lions (excepting megaherbivores, Stander 1991, 2006, 2007, 2008, 2018; Lion Rangers unpublished data; local prey data for spotted hyena were unavailable), are locally abundant, and of high conservation and tourism value (Brown and Ramey 2022; MEFT (Namibia Ministry of Environment, Forestry and Tourism) 2022; NACSO (Namibia Association of CBNRM Support Organizations) 2023). This cross‐species research design assessing spatial and temporal interactions enabled us to explicitly evaluate herbivore trait‐mediated trade‐offs under arid system constraints in a multi‐predator landscape.

Drawing on the LOF and risk‐sensitive foraging theory (Houston and Rosenström 2024), we developed four trait‐informed hypotheses about how environmental constraints and predation risk jointly structure herbivore behavior. First, in a seasonal, water‐limited system, environmental constraints (visibility/cover, distance to water, dry season progression) would explain more variance in daily herbivore detections than predator covariates for both grazers and browsers (Laundré et al. 2014). We predicted that environmental covariate effect sizes (and model inclusion frequencies) would generally exceed those of predator terms. Second, where spatial refuge options are constrained, herbivores will reduce diel overlap (Δ) with nocturnal carnivores rather than vacating resource patches (Tambling et al. 2015). We predicted that herbivore detections would be predominantly diurnal and that spatial co‐occurrence with predators would be common. Third, that megaherbivores would show minimal spatiotemporal avoidance of predators compared to smaller herbivores. We predicted weak or null predator coefficients in spatial models and that any timing shifts would be modest rather than indicating strong suppression. Finally, that herbivores would respond more strongly to recent predator activity rather than by proactively avoiding areas with chronic predator presence (Tambling et al. 2015; Palmer et al. 2017). We predicted that short‐term (0–6 h) time windows would indicate larger herbivore response to predator presence than delayed windows (6–24 h), with trait‐ and environment‐dependent interactions (e.g., stronger predator effects later in the dry season) exceeding main effects.

Together these hypotheses articulate a hierarchical LOF that emerges from the intersection of body size, foraging ecology, water dependence, and arid‐system seasonality. We evaluate these hypotheses using hierarchical Bayesian models of species detections, conditional logistic analyses testing reactive responses to recent predator events, and circular activity models quantifying diel overlap. This integrated, trait‐anchored approach clarifies when and why bottom‐up forces dominate spatial decisions and how top‐down risk is shifted in time, providing generalizable insights into predator–prey coexistence in variable, multi‐use landscapes.

## 2 Materials and Methods

### 2.1 Study Area

**FIGURE 1:**
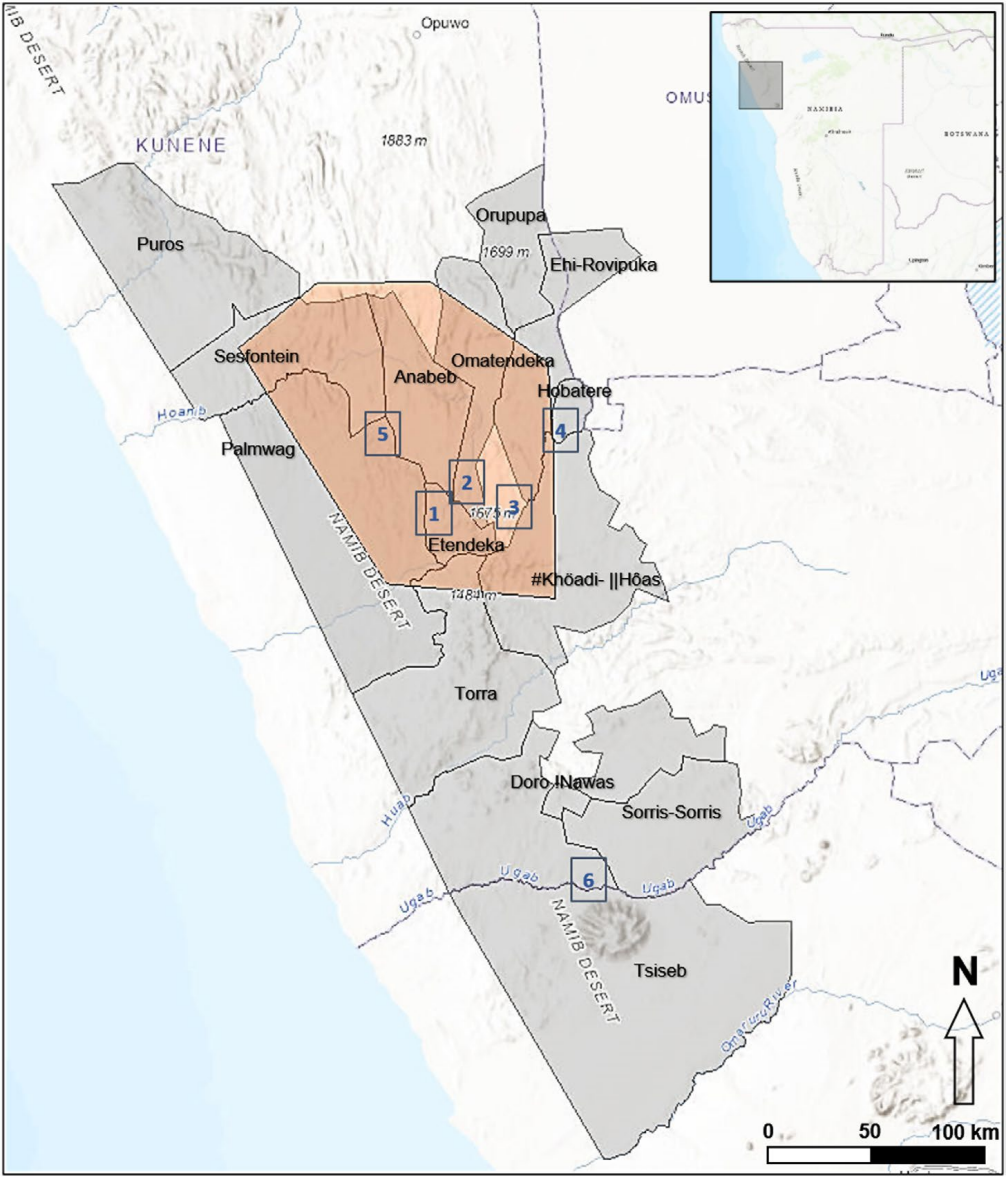
Map of study area showing communal conservancies and tourism concessions along with camera deployment areas (Etendeka #1, Otjiapa #2, Omirembue #3, Hobatere #4, Anabeb/Palmwag #5, Ugab #6) and core lion ( *Panthera leo* ) range (orange polygon) in northwest Namibia. Details in Table S1; Figure S1–S6.

This study was conducted across six areas in northwest Namibia (Figure 1), encompassing 106.97 km^2^ across five unfenced communal conservancies and three tourism concessions (Table S1; Figure S1–S6). The study region spanned core desert‐adapted lion range (Stander 2018; Heydinger, Muzuma, and Packer 2024), comprising unfenced, human‐influenced drylands.

Three primary ecological zones characterize the study area: Nama karoo with northwestern escarpment and inselberg vegetation, western highlands dominated by mopane ( *Colophospermum mopane* ), and the northern Namib Desert (Atlas of Namibia Team 2022). Soils are rocky and nutrient‐poor, and rainfall is scarce and unpredictable (< 200 mm annually and > 60% annual variability) generally following a west‐to‐east gradient. From 2000 to 2011, annual rainfall averaged 215 mm, supporting increases in wildlife and livestock numbers (Owen‐Smith 2010; NACSO (Namibia Association of CBNRM Support Organizations) 2023; Liebenberg unpublished data). From 2012 to 2023, rainfall dropped to an annual average of 67.5 mm, while potential evapotranspiration exceeded 2400 mm (Atlas of Namibia Team 2022); rainfall is also highly seasonal. During the wet season (Jan–May), high temperatures average 30.5°C with brief, localized showers; the cool dry season (Jun–Sep) has cooler nights (mean high = 28.5°C, lows ~14°C); and the warm dry season (Oct–Dec) brings extreme heat (mean high = 34.1°C), with prey concentrating in the shade of riverbeds and near water sources. Approximately 19,000 human residents inhabiting the study area and surrounding landscape derive their income primarily from semi‐nomadic pastoralism (Heydinger, Muzuma, Brassine, et al. 2024).

### 2.2 Camera Deployment and Independence

Across six dry‐season periods, we deployed a total of 374 motion‐triggered trail cameras (Panthera PoacherCam V7, New York, NY, USA) at waterholes (*n* = 18), along ephemeral riverbeds (*n* = 51), 4 × 4 tracks (*n* = 21), and game trails (*n* = 284), focusing on wildlife movement corridors and resource hotspots; camera spacing and positioning emphasized focal species detections—a commonly used approach in dryland systems (e.g., Portas et al. 2022, Table S1, Figure S1–S6 for deployment details). Cameras were field‐tested for detection distance (~30–40 m) suitable for both medium‐ (30–100 kg) and large‐bodied species (> 100 kg), set to low trigger sensitivity to minimize false activations, and calibrated for optimal flash brightness (white incandescent) for image clarity and detection distance. There was little evidence that flash deterred target species; avoidance would, if anything, lead to conservative detection estimates. Cameras operated 24/7 with a five‐second delay between single triggers and were checked every 2–3 weeks to replace batteries. Camera placement was guided by local expert trackers (Lion Rangers 2025) to maximize species detections in a heterogeneous, rugged landscape (Portas et al. 2022).

Images (~170,000) were AI‐classified using TrapTagger (traptagger.co.uk) and human‐verified (AI error < 0.05%). Camera data were filtered to separately retain species of interest—each focal species was assessed separately. For analyses, cameras sharing an ecological feature (e.g., waterhole) or movement corridor (e.g., game trail, 4 × 4 track) and within ~150 m of a shared, imaginary, central point (1–9 cameras) were grouped together as “camera clusters” to avoid pseudo‐replication in species detections. Clustering was performed post hoc by the camera deployment team and also based on landscape features. To further minimize pseudo‐replication, we applied a 30‐min independence rule, retaining only the first detection of each species within a 30‐min window (Welch et al. 2023). Efficacy of the camera cluster approach was checked for each species by running models without camera clustering (each camera site treated separately), with a similar 30‐min independence rule.

### 2.3 Environmental and Biotic Covariates

We compiled environmental and anthropogenic covariates from field assessments and remote sensing and transformed these as necessary for model fitting. We provide a full data dictionary (Table S2) listing measurement, units, temporal resolution, raw distribution, data transformations, and source of each covariate. *Vegetation cover* (1–3.5) indexed obstruction of green and woody vegetation. *Visibility* (1–4) was based on predominant line‐of‐sight distance across ≥ 51% of the viewshed from a camera. *Water proximity* (0–3) was based on Euclidean distance from camera to nearest water source. *Human use intensity* (0–4) combined accessibility and observed use (foot, vehicle, tourism, livestock)—routine researcher checks did not exceed minimum use intensity value. *Temperature* was based on historical models, as direct readings were not available. *Elevation*, *latitude*, *and longitude* were all based on camera cluster centre GPS location. *Dry‐season progression* measured days since the end of the wet season (31 May). *Lunar illumination* (% moon visibility), *NDVI*, and communal conservancy or tourism concession *management type* were also included, based on remote sensing data and official designations. Biotic covariates included apex *predator presence* (lion, spotted hyena); recorded as daily Bernoulli response (presence/absence ≤ 24 h prior to herbivore detection) at a camera cluster. *Detection rate* referred to the images taken per 100 camera‐nights per cluster for each herbivore or carnivore species (Cusack et al. 2017) and was used when modeling species co‐occurrence probability as a continuous predictor. *Long‐term lion activity* (0–2) quantified seasonal overlap with lion home range, based on the number of collared lions' core (50% kernel density estimator, KDE) and home ranges (95% KDE) overlapping with a camera site (Heydinger unpublished data) during the survey period. Environmental and biotic values were averaged across camera sites within each cluster (human use was averaged across camera clusters for each waterhole within a survey site). Continuous predictors with zeros were transformed as log(1 + *x*) to stabilize skew. Spatial and temporal predictors were standardized (z‐score scaled).

### 2.4 Bayesian Modeling Framework

For each herbivore species we fit four hierarchical Bernoulli candidate models with logit link, random intercepts for survey and sampling unit (camera cluster), and fixed effects for environmental covariates, human use, and predator metrics. Additionally, to assess species co‐occurrence, we fit generalized linear mixed‐effect models (GLMM) with detection rates of other herbivores (eland *Taurotragus oryx* , black‐faced impala *Aepyceros melampus peteersi*) and two subordinate predators (brown hyena *Parahyaena brunnea*, black‐backed jackal *Lupullela mesomelas*) as fixed effects, using stepwise selection to identify influential species‐level covariates for inclusion in Bayesian models (leopard *Panthera pardus* *n* = 37 and cheetah *Acinonyx jubatus* *n* = 14 detections were discarded due to small sample size). GLMMs were fit using lme4::glmer() function with bobyqa optimizer and maxfun = 25.

The four candidate models were: (1) full model with weak priors, a multilevel logistic regression including all environmental, spatial, and species co‐detection covariates; (2) horseshoe‐regularized full model, a replication of the full model incorporating a horseshoe regularization to induce shrinkage of weak or irrelevant predictors while retaining signals with strong posterior density (Piironen and Vehtari 2017a); (3) post hoc model using only ecologically justified and moderately informed covariates based on horseshoe model posterior shrinkage results removing predictors with near‐zero posterior estimates and credible intervals centered between −0.2 and 0.2 (Piironen and Vehtari 2017b). To avoid over‐shrinkage and preserve ecological interpretability, we adopted a Bayesian‐informed retention strategy for the post hoc model. Variables were retained based on moderate posterior support, ecological relevance, or hierarchical modeling structure. To prevent “pure interactions” any covariate participating in retained interactions was also included as a main effect. We relaxed strict 95% credible interval (CrI) thresholds and instead focused on biological plausibility and signal strength across species models. For each model, camera cluster and survey were treated as random effects. This hybrid approach aligns with best practices in ecological Bayesian analysis (e.g., Piironen and Vehtari 2017b). We also fit a (4) null model, including random intercepts for camera cluster and survey only.

We used weakly informative priors for models 1, 3, and 4: normal(0, 2) for fixed effects, normal(0, 5) for the intercept, and exponential(1) for group‐level standard deviations, and horseshoe regularization for model 2. Models were fit using Hamiltonian Monte Carlo sampling via cmdstanr (4 chains × 8000 iterations; 1000 warm‐up), with adapt_delta = 0.99 and max_treedepth = 12 to ensure convergence. All models were fit using the R packages (R Core Team 2024) brms (Buerkner 2017) and cmdstanr (Gabry et al. 2025).

Predictive performance was assessed using Leave‐One‐Out Cross Validation (LOO), extracting expected log pointwise predictive density (ELPD), standard error, and Bayesian *R* ^2^ for each model. Model weights were computed via stacking with loo_model_weights(), and used to generate model‐average predictions integrating across plausible alternatives. To quantify uncertainty, posterior predictions were generated using posterior_epred(), and summarized using posterior means and 95% CrI across 1000 draws per observation. Final inference focused on the post hoc models, which retained strong ecological signals while avoiding overfitting. Model comparisons and visualizations were performed using bayesplot (Gabry et al. 2019), tidyverse (Wickham et al. 2019), and tidybayes (Kay 2024). We report LOO, ELPD, SE, and stacking weights for all models (Table S3).

We interpreted predictor effects using multiple complementary metrics including when 95% CrI did not cross zero (strong); when 90% directionality of posterior distribution fell on one side of zero (moderate); and when the posterior mean was ≥ 1.0 distance from zero, the CrI on that side of zero exceeded two posterior estimate errors and was less than two posterior estimate errors on the other side of zero, and the finding aligned with ecological theory regarding herbivore space use and predator–prey relationships (weak).

### 2.5 Conditional Logistic Regression for Temporal Effects of Predator Presence

We used a time‐stratified case‐crossover design to test whether recent detections of predators (lions, spotted hyenas) influenced the short‐term probability of detecting focal herbivores. The approach controls for spatial, diel, and seasonal variation by matching each “event” (focal herbivore detection) with temporally and spatially proximate “control” hours when the same site was operational but the focal species was not detected. Detections were time‐stamped and assigned to “cluster‐days” running from 12:00 to 11:59 h the following day. Timestamps were standardized to hourly resolution, and for each camera cluster a binary detection matrix was created for lions, spotted hyenas, and each focal herbivore species. For each focal herbivore, all hourly detections were treated as cases, and up to five control hours without detections were randomly selected from the same cluster, month, and hour of day. Using rolling joins across the hourly series, we calculated the time since the last lion or spotted hyena detection and categorized exposure as 0–6 h (short‐term) or 6–24 h (delayed) after predator activity; the 6‐h threshold was selected based on observed diel pattern difference (below) and the need for samples of sufficient size.

We fitted conditional logistic regression models using the clogit() function in the survival package in Program R (Therneau 2023), with predator‐exposure indicators as predictors and a stratum for each case–control set. Coefficients were exponentiated to obtain odds ratios (ORs), interpreted as relative changes in detection odds of focal herbivores following recent predator presence. Confidence intervals (95% CI) were obtained and significance was assessed at *α* = 0.05. Each herbivore species was analyzed separately.

### 2.6 Activity Overlap Analysis

To evaluate temporal risk avoidance, we used circular statistics and kernel density estimation to quantify diel activity overlap between each herbivore species and apex predators at camera clusters where both were present. All timestamps were converted to radians and processed using the overlap (Meredith et al. 2024), circular (Agostinelli and Lund 2024), and activity (Rowcliffe 2023) packages in program R. Overlap coefficients (Δ, Ridout and Linkie 2009) were estimated using kernel density (Δ̂₁ for smaller samples, Δ̂₄ for larger), and 95% CI were derived using 1000 bootstrap iterations. Densities were plotted over a 24‐h cycle to visualize shared versus distinct activity periods. Overlap values were categorized as low (< 0.5), moderate (0.5–0.75), or high (> 0.75, Welch et al. 2023). Final plots showing kernel density functions and overlapping areas were generated using ggplot2 (Wickham 2016), allowing for direct visual comparison of herbivore and carnivore activity patterns across shared sites.

All analyses were conducted in program R v4.3+.

## 3 Results

We collected 11,022 trail camera detections (30‐min rule) over 8288 camera‐days across the six focal areas (Table S1 for details). For analysis, we condensed the 374 cameras deployed into 125 camera clusters—this yielded 5268 independent detections. Sensitivity checks with no camera clustering (each site treated separately) showed that clustering consistently increased the explanatory power of Bayesian models (*R* ^2^); cluster‐based models had narrower credible intervals and clearer post hoc estimates among significant variables and interactions for all species without changing the direction of effect. In addition to detections of each herbivore species (below), our analyses included 243 lion detections, 217 spotted hyena detections, 178 brown hyena detections, and 327 black‐backed jackal detections.

Our inferences relied on Bayesian post hoc models, selected for their parsimony and strong predictive performance as evaluated by expected log predictive density (ELPD) and leave‐one‐out cross‐validation (LOO, Table S3). These models retained ecologically meaningful predictors identified in full models while avoiding overfitting. Although full models with weak priors often yielded slightly higher R^2^ values, they performed worse under predictive stacking and were penalized for complexity. Horseshoe‐prior models aided in identifying weak or inconsistent effects, many of which were excluded from final models.

### 3.1 Species‐Level Patterns

#### 3.1.1 Gemsbok

**FIGURE 2:**
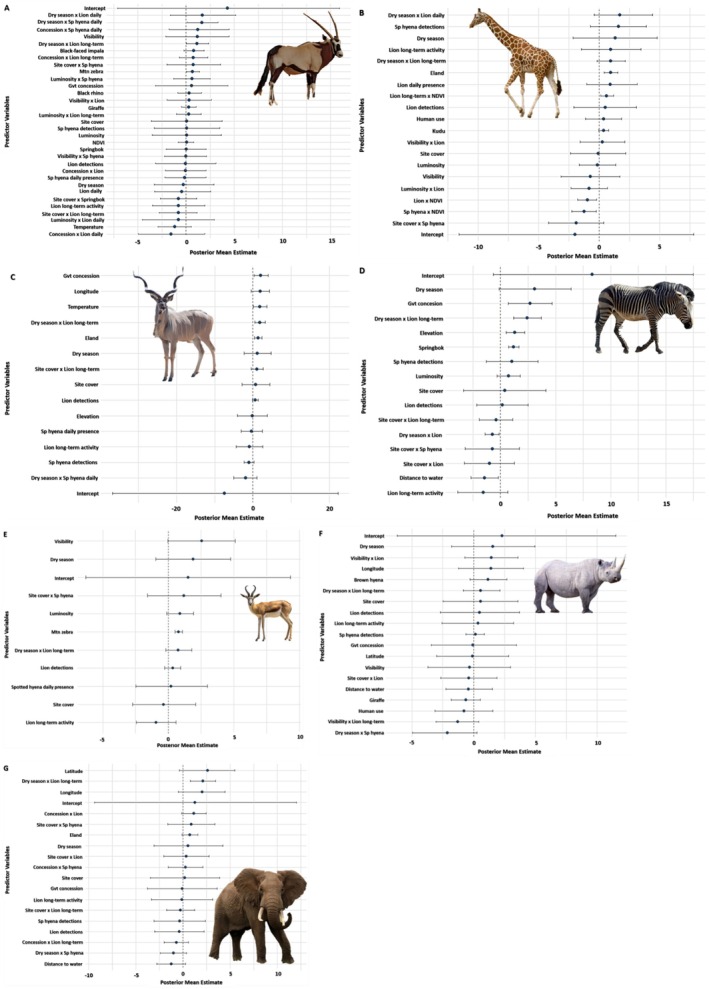
Forest plots of predictor effects on herbivore (gemsbok *Oryx gazella* (A), southern giraffe *Giraffa giraffa* (B), kudu *Tragelaphus strepsiceros* (C), Hartmann's mountain zebra *Equus zebra* (D), springbok *Antidorcas marsupialis* (E)) and megaherbivore (black rhinoceros *Diceros bicornis* (F), African bush elephant *Loxodonta africana* (G)) detections at camera clusters in northwest Namibia, based on Bayesian post hoc (top‐performing) models with logit function. Plots display posterior mean estimates and 95% credible intervals for predictor variables (main and interaction). Credible intervals not overlapping zero indicate strongest effects. (Species) daily, indicates daily presence of relevant species; detections is detection rate of species, lion long‐term is chronic lion activity as measured by GPS/satellite collars (see Table S2).

Trail cameras recorded 306 independent gemsbok detections, with detection probability shaped primarily by environmental context and moderate predator interactions. In the post hoc Bayesian model (Bayes *R* ^2^ = 0.41, 95% CrI: 0.35–0.47; model weight = 0.81, Table S3), few covariates showed strong effects, though detections increased with mountain zebra detections (*β* = 0.66, 95% CrI: 0.03–1.39, Figure 2, Figures S7–S14; Table 1, Table S4); random effects for both camera cluster (*σ* = 0.37, 95% CrI: 0.01–0.96) and survey area (*σ* = 5.13, 95% CrI: 2.76–8.40) reflected the strong influence of spatial heterogeneity on gemsbok detections. Visibility was moderately associated with gemsbok detection (*β* = 1.19, 95% CrI: −2.11–4.46, Table 1), suggesting selection of more open habitats. Detection decreased with temperature (*β* = −1.17, 95% CrI: −2.92–0.54), consistent with reduced activity under extreme heat.

**TABLE 1:** Summary of Bayesian post hoc (top‐performing) model predictors across herbivores species recorded on trail cameras in northwest Namibia.

Species	Key environmental predictors	Key apex predator predictors	Interaction terms (predator × environment)
Gemsbok (*R* ^2^ = 0.41)	Mountain zebra detections** (+), Visibility (+), Temperature (−)	None retained	Lion long‐term × Dry season** (+), Spotted hyena presence × Dry season** (+), Lion presence × Dry season (+), Lion presence × Concession (−), Spotted hyena presence × Concession (+)
Giraffe (*R* ^2^ = 0.39)	Eland detections** (+), Dry season (+)	Spotted hyena detections (+)	Lion × NDVI ** (−) Spotted hyena × NDVI** (−); Lion presence × Dry Season* (+), Lion long‐term × Dry Season* (+), Spotted hyena × Site Cover* (−)
Kudu (*R* ^2^ = 0.40)	Temperature** (+), Concession** (+), Eland detections** (+), Longitude* (+), Dry season (+)	Spotted hyena detections* (−)	Lion long‐term × Dry season** (+), Lion long‐term × Site cover (+), Spotted hyena presence × Dry season (−)
Mountain zebra (*R* ^2^ = 0.37)	Elevation** (+), Concession** (+), Springbok detections** (+), Dry season* (+), Distance to water* (−)	Lion long‐term (−), Spotted hyena detections (+)	Lion long‐term × Dry season** (+), Lion × Dry season* (−), Lion × Site cover (−)
Springbok (*R* ^2^ = 0.32)	Mountain zebra detections** (+), Visibility* (+), Luminosity* (+), Dry season (+)	None retained	Spotted hyena presence × Site cover (+)
Black rhino (*R* ^2^ = 0.34)	Brown hyena detections* (+), Longitude (+), Dry season (+)	None retained	Spotted hyena presence × Dry season* (−), Lion × Visibility (+), Lion long‐term × Visibility (−)
Elephant (*R* ^2^ = 0.41)	Distance to water* (−), Latitude* (+), Eland detections* (+), Longitude (+)	None retained	Lion long‐term × Dry season (+), Lion × Concession* (+), Spotted hyena × Dry season (−)

*Note:* Signs (+/−) indicate direction of effect on detection probability. Strength of predictor: **predictors where 95% credible interval (CrI) does not cross zero; *predictors where 90% of CrI falls on one side of zero; other predictors included when posterior estimate ≥ 1.0 distance from zero, CrI was > 2 estimate errors on the posterior mean side of zero and was < 2 posterior estimate errors on the other side of zero, and aligned with ecological theory. Only predictors retained in the post hoc Bayesian models were included. R^*2*^, post hoc model explanation of variance in species detections (Complete main and interactive predictors are reported in Tables S4–S10).

**FIGURE 3:**
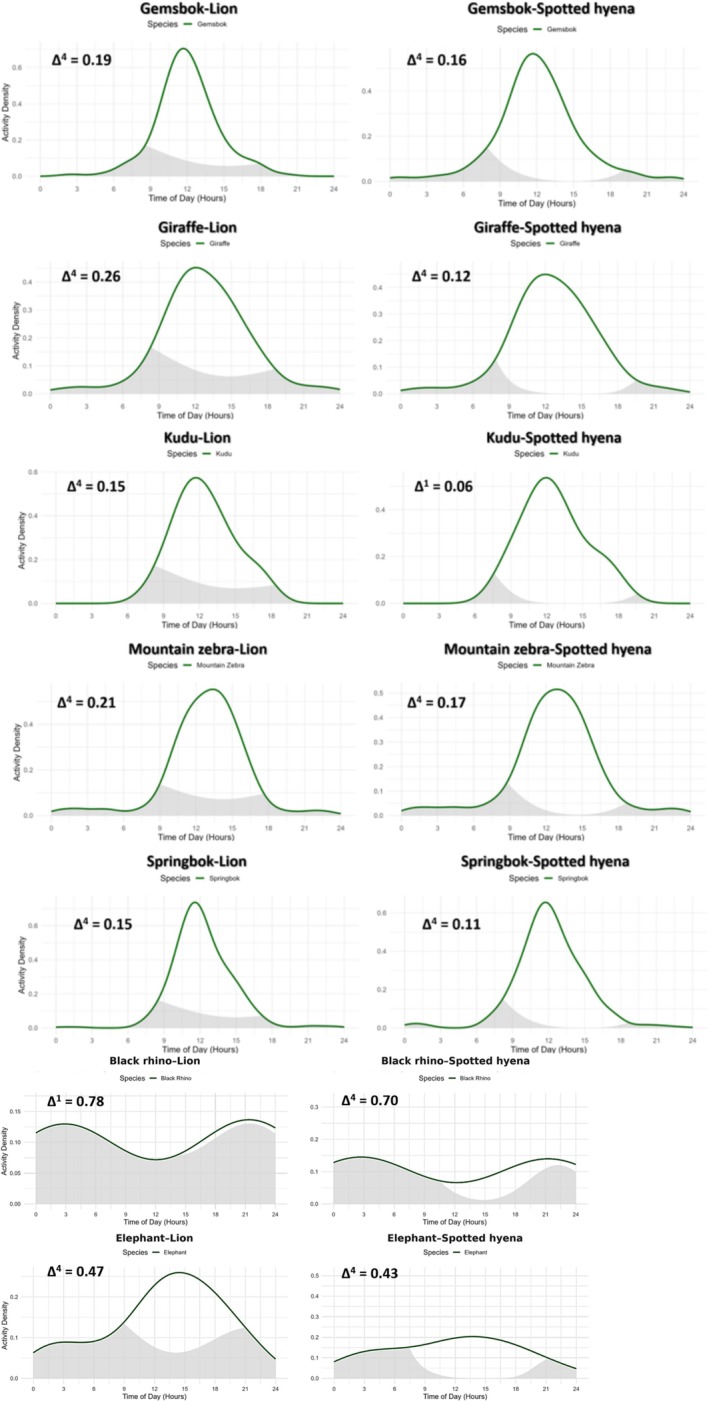
Temporal activity overlap between seven herbivore species (gemsbok *Oryx gazella* , southern giraffe *Giraffa giraffa* , kudu *Tragelaphus strepsiceros* , Hartmann's mountain zebra *Equus zebra* , springbok *Antidorcas marsupialis* , black rhinoceros *Diceros bicornis* , African bush elephant *Loxodonta africana* ) and apex predators (lion *Panthera leo* , spotted hyena *Crocuta crocuta* ) in northwest Namibia. Each panel shows kernel density estimates of diel activity (0–24 h) for herbivores (green lines) and overlapping activity (gray shading) with lions (left column) and spotted hyenas (right column). Δ estimates range from 0 (no overlap) to 1 (complete overlap), based on kernel density estimation of diel activity patterns. (Complete results in Figure S11.)

Predator effects were generally weak. However, interactions indicated seasonal and environmental modulation. Detection probability increased as the dry season progressed when spotted hyenas were present (*β* = 1.65, 95% CrI: 0.11–3.34) and with high lion long‐term activity (*β* = 1.13, 95% CrI: 0.38–2.36), suggesting risk tolerance during periods of resource scarcity. Conditional logistic regression revealed short‐term (0–6 h) spotted hyena presence significantly reduced gemsbok detections (OR ≈0.33, 95% CI = 0.11–0.99, *p* = 0.048, Table 2), while delayed (6–24 h) spotted hyena and all lion effects were nonsignificant. Temporal kernel density analyses showed low diel overlap (Δ^4^ = 0.19 with lions; Δ^4^ = 0.16 with spotted hyenas, Figure 3; Table S11) at camera clusters where gemsbok and apex carnivores were both present. At all camera clusters, gemsbok activity was concentrated near midday (mean activity time: 12.11 h, 95% CI: 11.80–12.42 h). In contrast, both predators (lions mean activity time = 2.20 h, 95% CI: 1.49–2.9 h; spotted hyenas mean activity time: 2.92 h, 95% CI: 2.49–3.39 h) were primarily nocturnal, with peak activity concentrated from after midnight to well before dawn.

**TABLE 2:** Conditional logistic regressions for focal herbivore (gemsbok *Oryx gazella* , southern giraffe *Giraffa giraffa* , kudu *Tragelaphus strepsiceros* , Hartmann's mountain zebra *Equus zebra* , springbok *Antidorcas marsupialis* ) and megaherbivore (black rhinoceros *Diceros bicornis* , African bush elephant *Loxodonta africana* ) hourly detections as an effect of apex predator (lion *Panthera leo* , spotted hyena *Crocuta crocuta* ) detections at camera clusters in northwest Namibia.

Species	Effect	Coefficient	SE	OR	95% CI	*p*
Gemsbok	Lion (0–6)	0.475	0.66	1.61	0.44–5.91	0.475
*n*‐events = 260	(6–24)	−0.471	0.63	0.62	0.18–2.13	0.452
Concordance = 0.526 (±0.01)	Spotted hyena (0–6)	−1.114	0.56	0.33	0.11–0.99	0.048*
Likelihood ratio: *χ* ^2^ = 7.64, 4 df, *p* = 0.1	(6–24)	−0.415	0.36	0.66	0.32–1.33	0.245
Giraffe	Lion (0–6)	−0.208	0.41	0.81	0.36–1.81	0.611
*n*‐events = 351	(6–24)	−0.270	0.36	0.76	0.38–1.54	0.451
Concordance = 0.507 (±0.01)	Spotted hyena (0–6)	−0.032	0.57	0.97	0.32–2.97	0.955
Likelihood ratio: *χ* ^2^ = 5.17, 4 df, *p* = 0.3	(6–24)	0.823	0.37	2.28	1.10–4.73	0.027*
Kudu	Lion (0–6)	0.910	0.65	2.48	0.69–8.94	0.164
*n*‐events = 199	(6–24)	0.641	0.53	1.90	0.67–5.36	0.227
Concordance = 0.518 (±0.01)	Spotted hyena (0–6)	−16.477	3989.10	0.00	Inf	0.997
Likelihood ratio: *χ* ^2^ = 4.8, 4 df, *p* = 0.3	(6–24)	−0.267	0.60	0.77	0.24–2.47	0.655
Mountain zebra	Lion (0–6)	−0.690	0.39	0.50	0.23–1.08	0.079.
*n*‐events = 1174	(6–24)	0.230	0.27	1.26	0.75–2.12	0.388
Concordance = 0.522 (±0.01)	Spotted hyena (0–6)	−1.302	0.32	0.27	0.14–0.51	0.000***
Likelihood ratio: *χ* ^2^ = 33.22, 4 df, *p* = 0.00001	(6–24)	0.482	0.20	1.62	1.10–2.38	0.014*
Springbok	Lion (0–6)	−0.428	0.49	0.65	0.24–1.72	0.386
*n*‐events = 650	(6–24)	−0.421	0.31	0.66	0.36–1.21	0.176
Concordance = 0.518 (±0.01)	Spotted hyena (0–6)	−0.105	0.42	0.90	0.40–2.03	0.800
Likelihood ratio: *χ* ^2^ = 3.21, 4 df, *p* = 0.5	(6–24)	−0.191	0.23	0.83	0.53–1.29	0.397
Black Rhino	Lion (0–6)	−0.990	0.84	0.37	0.07–1.93	0.239
*n*‐events = 53	(6–24)	18.699	9814.28	Inf	Inf	0.998
Concordance = 0.614 (±0.05)	Spotted hyena (0–6)	−1.618	0.77	0.20	0.04–0.90	0.035*
Likelihood ratio: *χ* ^2^ = 9.79, 4 df, *p* = 0.04	(6–24)	0.130	0.95	1.14	0.18–7.33	0.891
Elephant	Lion (0–6)	−2.772	0.62	0.06	0.02–0.21	0.000***
*n*‐events = 228	(6–24)	0.573	0.38	1.77	0.83–3.77	0.137
Concordance = 0.582 (±0.02)	Spotted hyena (0–6)	−1.367	0.69	0.25	0.07–0.99	0.048
Likelihood ratio: *χ* ^2^ = 52.79, 4 df, *p* < 0.0001	(6–24)	0.285	0.49	1.33	0.51–3.46	0.559

*Note:* Focal species were tested for effect by apex carnivores in short‐term (0–6 h) and delayed (6–24 h) windows. Coefficients and standard errors; OR, odds ratio of herbivore following carnivore detection based on exponentiated coefficient; odds ratio 95% confidence interval (CI)—CIs not crossing 1 indicate clear signal; *p*, significance ‘.’ *α* = 0.1; ‘*’ *α* = 0.05; ‘**’ *α* = 0.01; ‘***’ *α* < 0.001.

#### 3.1.2 Giraffe

Trail cameras recorded 471 independent giraffe detections, with detection probability influenced by both environmental and predator covariates and evidence of interaction between vegetation structure and predator presence. Random effects for both camera cluster (*σ* = 0.40, 95% CrI: 0.02–1.01, Table S5) and survey area (*σ* = 5.12, 95% CrI: 2.74–8.47) reflected the strong influence of spatial heterogeneity on giraffe detections. In the Bayesian post hoc model (Bayes *R* ^2^ = 0.39, 95% CrI: 0.33–0.45; model weight = 1.00, Table S3), giraffe detections increased with eland detections (*β* = 0.96, 95% CrI: 0.41–1.55, Figure 2, Figures S15–S21; Table 1), suggesting spatial association among these large browsers. Detections also increased into the dry season (*β* = 1.34, 95% CrI: −2.16–4.80), though with a wide credible interval.

Predator effects interacted strongly with vegetation greenness and seasonal timing. Giraffe detections decreased with increasing NDVI where lion (*β* = −0.96, 95% CrI: −1.79– −0.21) and spotted hyena detections were high (*β* = −1.23, 95% CrI: −2.27– −0.25). In contrast, a positive lion long‐term × NDVI interaction (*β* = 0.64, 95% CrI: 0.13–1.22) suggests an inability to avoid valuable resource patches, even when lions have occupied the area. Detections increased with lion daily presence (*β* = 1.72, 95% CrI: −0.42–4.42) and long‐term activity (*β* = 0.97, 95% CrI: −0.18–2.15) as the dry season progressed.

In the conditional logistic regression, short‐term predator effects were weak, but delayed (6–24 h) spotted hyena presence significantly increased giraffe detections (OR = 2.28, 95% CI = 1.10–4.43, *p* = 0.027, Table 2), suggesting reactive avoidance with delayed return to areas of overlap. Temporal kernel density estimates revealed low diel overlap (Δ^4^ = 0.26 with lions; Δ^4^ = 0.12 with spotted hyenas, Figure 3; Table S11) at camera clusters where giraffe and apex carnivores were both present. Across all camera clusters, giraffe displayed a clear diurnal activity pattern with peak activity around midday (mean activity time = 12.98 h, 95% CI: 12.62–13.31).

#### 3.1.3 Kudu

Trail cameras recorded 291 independent kudu detections with both environmental seasonal predictors and long‐term predator activity influencing detection probability. In the Bayesian post hoc model (Bayes *R* ^2^ = 0.40, 95% CrI: 0.32–0.48; model weight = 1.00, Table S3), kudu detections increased with temperature (*β* = 1.85, 95% CrI: 0.09–3.63, Figure 2, Figures S22–S26; Table 1, Table S6), likely reflecting higher activity near waterholes during periods of environmental stress. Kudu were also more frequently detected in tourism concessions than in communal conservancies (*β* = 2.05, CrI: 0.28–3.98) and in areas further east (*β* = 1.91, CrI: −0.49–4.25), potentially due to lower human disturbance or different habitat structure in these areas. Additionally, kudu detection was positively associated with eland presence (*β* = 1.31, CrI: 0.33–2.38), suggesting habitat convergence. Random effects for both camera cluster (*σ* = 1.23, 95% CrI: 0.51–2.08) and survey area (*σ* = 4.12, 95% CrI: 2.1–7.23) reflected the strong influence of spatial heterogeneity on kudu detections.

Main predator effects were generally weak, though spotted hyena detections showed a negative main effect (*β* = −1.00, CrI: −2.34–0.24). The strongest predictor was a positive interaction between dry season progression and lion long‐term activity (*β* = 1.84, CrI: 0.51–3.21). In contrast, spotted hyena detections decreased kudu detection probability (*β* = −1.00, CrI: −2.34–0.24), indicating potential reactive avoidance.

Conditional logistic regression revealed no significant short‐term or delayed predator effects (Table 2), though positive coefficients for lions (OR = 1.90–2.48) and weak negative coefficients for spotted hyenas (OR = 0.33–0.77) suggest potential low‐level behavioral modulation. Temporal kernel density analyses indicated low diel overlap (Δ^4^ = 0.15 with lions; Δ^1^ = 0.06 with spotted hyenas, Figure 3; Table S11). Kudu also showed strong diurnal activity centred around midday (mean activity time = 12.61 h, 95% CI: 12.30–12.96), largely avoiding the nocturnal peaks of lions and spotted hyenas.

#### 3.1.4 Mountain Zebra

Trail cameras recorded 1652 independent mountain zebra detections, with detection probability strongly influenced by environmental features and seasonally‐mediated predator effects. In the Bayesian post hoc model (Bayes *R* ^2^ = 0.37, 95% CrI: 0.32–0.42; model weight = 0.64, Table S3), mountain zebra detections increased with elevation (*β* = 1.28, 95% CrI: 0.49–2.19, Figure 2, Figures S27–S32; Table 1, Table S7) and within concession areas (*β* = 2.67, 95% CrI: 0.69–4.68). Detections were also positively associated with springbok detections (*β* = 1.17, 95% CrI: 0.72–1.66), reflecting shared habitat use among grazers and indicating possible benefits from mixed‐species vigilance. One of the strongest constraints on mountain zebra detection was distance from water (*β* = −1.45, 95% CrI: −2.70 to −0.22). Correspondingly, dry season progression had a strong positive effect on detection (*β* = 3.12, 95% CrI: −0.13–6.39), consistent with increased reliance on water as resources become scarce. Random effects for both camera cluster (*σ* = 1.43, 95% CrI: 0.91–2.07) and survey area (*σ* = 8.96, 95% CrI: 5.95–13.07) reflected the strong influence of spatial heterogeneity on mountain zebra detections.

Predator effects were shaped by seasonal context: mountain zebra detections decreased when lion detections increased as the dry season progressed (*β* = −0.72, 95% CrI: −1.40– −0.11), but mountain zebra detections increased with lion long‐term activity (*β* = 2.42, CrI: 1.19–3.72) under the same conditions. Among main effects, long‐term lion activity showed a negative effect (*β* = −1.56, 95% CrI: −3.90–0.67), while mountain zebra detections increased with spotted hyena detections (*β* = 1.05, 95% CrI: −1.30–3.39).

Conditional logistic regression revealed pronounced short‐term (0–6 h) avoidance of spotted hyenas (OR = 0.27, 95% CI = 0.14–0.51, *p* < 0.001, Table 2), coupled with an increase in delayed (6–24 h) detections after spotted hyena activity (OR = 1.62, 95% CI = 1.10–2.38, *p* = 0.014), suggesting time‐lagged habitat use following predator activity. Short‐term lion effects were marginally negative (OR = 0.50, *p* = 0.079), while delayed lion activity (6–24 h) had no detectable influence. Temporal overlap was low (Δ^4^ = 0.21 with lions; Δ^4^ = 0.17 with spotted hyenas, Figure 3; Table S11), indicating strong diurnal activity by mountain zebra relative to nocturnal predator activity. Mountain zebra diel activity was tightly clustered around midday (mean activity time = 13.23 h, 95% CI: 13.1–13.37).

#### 3.1.5 Springbok

Trail cameras recorded 826 independent springbok detections, with detection probability shaped primarily by environmental visibility and co‐occurrence with other grazers, with only weak evidence of predator effects. In the Bayesian post hoc model (Bayes *R* ^2^ = 0.32, 95% CrI: 0.25–0.38; model weight = 0.92, Table S3), detection probability increased with visibility (*β* = 2.56, 95% CrI: −0.05–5.12, Figure 2, Figures S33–S38; Table 1, Table S8), and luminosity (*β* = 0.90, CrI: −0.11–1.92), suggesting the importance of clear and far‐seeing lines‐of‐sight, even in low‐light conditions. Detections were also strongly positively associated with mountain zebra detections (β = 0.77, CrI: 0.50–1.07) and dry season depth (*β* = 1.89, CrI: −0.95–4.76). Random effects for both camera cluster (*σ* = 0.74, 95% CrI: 0.14–1.34) and survey area (*σ* = 5.69, 95% CrI: 3.20–9.07) reflected the strong influence of spatial heterogeneity on springbok detections.

Predator variables showed weak and uncertain effects, with credible intervals broadly overlapping zero. Springbok detections increased in areas where spotted hyena were present with high levels of site cover (*β* = 1.19, CrI: −1.60–4.02) potentially reflecting active tracking of springbok by spotted hyena into certain areas. Conditional logistic regression similarly revealed no significant short‐term (0–6 h) or delayed (6–24 h) responses to either lions or spotted hyenas, though all predator coefficients were negative, suggesting mild avoidance tendencies (Table 2).

Temporal kernel density estimates showed low diel overlap with both predators (Δ^4^ = 0.15 with lions; Δ^4^ = 0.11 with spotted hyenas, Figure 3; Table S11) and springbok exhibited tightly clustered diurnal activity pattern centred around midday (mean activity time = 12.32 h, 95% CI: 12.16–12.48).

#### 3.1.6 Black Rhino

Trail cameras recorded 119 independent black rhino detections. Bayesian post hoc models (Bayes *R* ^2^ = 0.34, 95% CrI: 0.25–0.43; model weight = 0.79, Table S3) identified limited environmental or spatial predictors with strong effects on detection probability; random effects for both camera cluster (*σ* = 0.47, 95% CrI: 0.01–1.50, Table S9) and survey area (*σ* = 2.23, 95% CrI: 0.01–5.68) reflected the strong influence of spatial heterogeneity on black rhino detections.

No environmental or predator‐based parameters had credible intervals that excluded zero (Figure 2, Figures S39–S43; Table 1), though weak positive associations were observed with dry season progression (*β* = 1.57, CrI: −1.81–4.97), longitude (*β* = 1.41, CrI: −1.22–4.04), and brown hyena detections (*β* = 1.18, 95% CrI: −0.31–2.69). Marginal plots indicate declining detection with increased human use, although high uncertainty (*β* = −0.77, CrI: −3.12–1.54) likely reflects the dual influence of monitoring efforts in rhino areas (savetherhinotrust.org) and the species' avoidance of anthropogenic disturbance.

Predator main effects were largely absent, but several predator × habitat interactions suggest conditional responses to risk. Notably, black rhino detection was negatively associated with high long‐term lion activity in open areas (*β* = −1.29, CrI: −3.04–0.43). Conversely, at high visibility sites with relatively high lion detections (*β* = 1.43, CrI: −0.73–3.60), black rhino detection probabilities increased. In contrast, probability declined with spotted hyena presence as the dry season progressed (*β* = −2.11, CrI: −4.95–0.26), pointing to spotted hyena avoidance, even during resource‐limited periods.

Conditional logistic regressions indicated significantly lower detection probability immediately (0–6 h) following spotted hyena detections (OR = 0.20, 95% CI = 0.04–0.90, *p* = 0.035, Table 2), while lion activity effects were non‐significant. Black rhino detections exhibited a bimodal nocturnal‐crepuscular activity pattern differing from smaller bodied herbivores (mean activity time = 0.29 h), with peaks occurring during the early night and again before dawn, and high diurnal overlap with predator activity (Δ^1^ = 0.78 with lions; Δ^4^ = 0.70 with spotted hyenas, Figure 3; Table S11).

#### 3.1.7 Elephant

Trail cameras recorded 377 independent elephant detections. The Bayesian post hoc regression model (Bayes *R* ^2^ = 0.41, 95% CrI: 0.34–0.47; model weight = 0.97, Table S3) identified limited environmental predictors (Figure 2, Figures S44–S50; Table 1, Table S10), though detection probability increased with latitude (*β* = 2.62, 95% CrI: −0.40–5.49) and longitude (*β* = 2.02, 95% CrI: −0.51–4.47), suggesting greater presence in more productive northerly and easterly areas of the study area. Distance from water was negatively associated with detection (*β* = −1.23, 95% CrI: −2.79–0.24), underscoring the use of varying travel routes, where cameras may not have been placed, to access water sources. A positive association with eland detections (*β* = 0.71, 95% CrI: −0.11–1.57) likely reflected shared habitat use. Random effects for both camera cluster (*σ* = 0.75, 95% CrI: 0.14–1.38) and survey area (*σ* = 4.03, 95% CrI: 5.95–13.07) reflected the strong influence of spatial heterogeneity on elephant detections.

Predator main effects were largely absent, as expected given elephants' limited vulnerability to predation (Whyte 2005). However, interaction terms revealed how apex predators may contribute to elephant space use under constrained conditions. Detection probability increased significantly in areas of high long‐term lion activity during the dry season (*β* = 2.09, 95% CrI: 0.77–3.45), but declined in areas with high spotted hyena detections (*β* = −1.02, 95% CrI: −2.43–0.39), suggesting reactive avoidance of spotted hyenas even under resource‐limited conditions and despite elephants' size. This pattern was echoed in interaction plots showing detection probabilities diverging across spotted hyena detection rates with increasing dry season depth.

Conditional logistic regression confirmed significant short‐term (0–6 h) avoidance of lions (OR = 0.06, 95% CI = 0.02–0.21, *p* < 0.001, Table 2) and weaker but still notable avoidance of spotted hyenas (OR = 0.25, 95% CI = 0.07–0.99, *p* < 0.048), while delayed predator presence (6–24 h) showed no significant effects. Elephant detections exhibited predominantly nocturnal to crepuscular activity patterns (mean activity time = 14.99 h, 95% CI: 14.08–15.96), with activity peaking between late evening and midnight and tapering toward midday (Δ^4^ = 0.47 with lions; Δ^4^ = 0.43 with spotted hyenas, Figure 3; Table S11). Additionally, activity periods coinciding with typical human activity—contrasting with the nocturnal patterns of black rhino—suggest that elephants do not exhibit the same level of human avoidance as black rhinos.

## 4 Discussion

In arid ecosystems with limited available resources, where spatial flexibility is constrained and environmental stress can be acute, herbivores must navigate a complex trade‐off between acquiring resources and avoiding predation. Across seven large herbivore species, our data demonstrate that space use is governed by bottom‐up constraints while predator risk is buffered in time, producing consistent low diel overlap with nocturnal carnivores and trait‐dependent moderation of spatial responses. By demonstrating that bottom‐up environmental constraints and temporal risk mediation operate in concert, our study contributes to the broader ecological understanding of how prey species resolve this complex trade‐off. These findings refine the LOF framework and underscore the importance of spatiotemporal plasticity in shaping herbivore behavior under environmental stress.

### 4.1 Bottom‐Up Dominance and Guild Contrasts

Across herbivore species, environmental context outweighed predator activity in structuring detections, consistent with our first hypothesis and prior research in arid and seasonal ecosystems (Corp et al. 1998; Riginos 2015); random effects for all Bayesian models underscore the importance of spatial heterogeneity in landscape use. For grazers (mountain zebra, springbok, gemsbok—mixed feeder), Bayesian models highlighted visibility/site cover, luminosity, elevation, and grazer co‐occurrence (mountain zebra‐springbok) as key predictors, whereas predator main effects were weak or contingent. Grazers also showed strongly diurnal activity with low nocturnal overlap with lions and spotted hyenas, indicating grazers' reliance upon time of day rather than displacement to manage risk—linking together our first and second hypotheses. However, mountain zebra may also be forced to modulate their behavior in response to lion activity more than springbok or gemsbok—as evidenced in Bayesian model interaction effects—perhaps due to lions targeting mountain zebra, which may compose up to 50% of local lion diets (Lion Rangers unpublished data). Even so, predator effects were short‐lived or context‐dependent: e.g., modest suppression of gemsbok by spotted hyena; brief avoidance with rapid spatial reoccupation by mountain zebra. These results point to a risk‐tolerant grazer (and mixed grazer‐browser) strategy, reinforcing that bottom‐up conditions set the baseline, grazing efficiency is prioritized, and proactive spatiotemporal strategies favoring visibility are preferred to reactive avoidance. The inclusion of luminosity as a relevant covariate for visibility is intriguing here, as grazers were primarily diurnal.

Browsers (giraffe, kudu) likewise conformed to bottom‐up dominance and diurnal avoidance of predators, but with greater behavioral flexibility in structurally complex habitats. Detection probability varied with vegetation structure (e.g., NDVI/site cover) and season, and predator effects emerged mainly through interactions with habitat rather than as strong main effects—consistent with behavioral adjustments where forage and concealment co‐vary. Both browsers were diurnal—limiting direct encounters with nocturnal predators—but showed tolerance of lion long‐term activity at shared resource patches, and only weak, reactive signals of spotted hyena avoidance. Collectively, these patterns reveal a functional divergence between grazers, which maintained more fixed strategies of predator avoidance centred around high‐visibility areas where grazing may be more abundant and predators easier to detect, and browsers, whose reliance on denser vegetation patches demanded a fine‐tuned strategy and tolerance for predator activity, most clearly evident in Bayesian model interactions effects. These findings support guild‐specific refinements to the LOF framework that emphasize spatiotemporal complexity and flexible behavioral responses (Palmer et al. 2022).

### 4.2 Spatiotemporal Unification of Risk Responses

Across herbivore guilds, the progression of the dry season drove herbivore movements and unified spatial and temporal components of the LOF. The effects of the dry season and temporal behavioral adjustments should be considered in concert. As the dry season progressed and resources contracted waterholes and riparian areas, spatial avoidance options diminished, increasing the likelihood of predator interactions. Temporal avoidance thus becomes increasingly important. Consistent with our second hypothesis, herbivores mitigated predation risk primarily through time‐of‐day shifts rather than spatial displacement. Across guilds, low temporal overlap with apex predators (Δ ≈0.11–0.21 for grazers; Δ ≈0.06–0.26 for browsers) confirms that diel partitioning is the dominant anti‐predator avoidance strategy in this landscape, consistent with findings from mesic savannas (e.g., Tambling et al. 2015), and newly demonstrated here for an arid ecosystem. This strategy entails a clear trade‐off: minimizing predation risk at the expense of increased thermal stress.

These patterns indicate that the LOF functions as a hierarchical spatial–temporal process: when environmental constraints compress space, species can rely on temporal adjustment strategies to maintain coexistence without broadscale displacement. Notably, the midday concentration of herbivore activity diverges from patterns observed in less arid savannas, where ambient temperature and nighttime illumination more strongly structure herbivore activity (Owen‐Smith et al. 2020). At the same time, our results echo findings from wetter systems that document temporal segregation where spatial refuges may be more widely available (e.g., Valeix et al. 2009; Tambling et al. 2015; Kohl et al. 2018).

### 4.3 Reactive Versus Proactive Risk Responses

Across species (excluding megaherbivores, below), our results support what we term a “proactive‐reactive” response to predator activity, providing qualified confirmation of our fourth hypothesis. Focal herbivores proactively minimized encounters with predators by anchoring activity during daylight hours (low Δ), thus pre‐empting many short‐term window effects that conditional logistic models would otherwise detect. Where predator signals emerged, they were reactive, brief, and largely mediated by environmental variables—e.g., transient mountain zebra avoidance; weak or absent short‐term effects for springbok—with browser responses to predators only strengthened through interactions with habitat/dry season progression. Together, Bayesian models, conditional logistic regressions, and overlap analyses converged on a single pattern: temporal partitioning raises spatial tolerance, allowing continued use of predator‐occupied sites while keeping encounter risk low.

Predator identity further structured responses. Herbivores showed stronger reactive avoidance of spotted hyenas than lions, whereas tolerance of lion long‐term activity areas often increased as the dry season deepened. We infer two complementary mechanisms relevant to the LOF framework: (i) hunting mode—coursing spotted hyenas (Kruuk 1974) may be more reliably avoided when they are known to be present than are ambush‐reliant lions (Packer 2023); (ii) territorial control near scarce resources—lions' ability to hold space at productive resource patches, even to the exclusion of other predators (Creel and Creel 1996; Swanson et al. 2014), creates another layer of risk–reward trade‐offs for prey species. These results suggest useful refinements to the LOF framework: incorporate predator territoriality/interference capacity and hunting mode × landscape covariates as explicit modifiers to assess when risk elicits reactive suppression versus when proactive temporal scheduling suffices to maintain coexistence.

### 4.4 Megaherbivores and Upper‐Guild Stability

While megaherbivores (black rhino, elephant) occupied a low‐risk domain within the predator–prey hierarchy, both species exhibited behavioral adjustments to predators—refining hypothesis three. Both species partially segregated their nocturnal activity from predators: while lion and spotted hyena activity was concentrated between midnight and pre‐dawn hours, black rhino and elephants minimized thermal stress via nighttime activity but still largely avoided this time window. Black rhinos' modest avoidance of spotted hyenas, detected in both Bayesian post hoc (interaction effects) and temporal analyses, suggests that even megaherbivores may adjust activity to reduce harassment at shared high‐value resources patches (cf. Macdonald and Johnson 2015; le Roex et al. 2019), such as near waterholes. Black rhino avoided human activity (cf. Odendaal‐Holmes et al. 2014), with clear signals possibly conflated due to anti‐poaching patrols in known rhino areas.

Bayesian models for each megaherbivore species indicated weak or context‐dependent predator effects, with general tolerance of lion long‐term activity coupled with more clear avoidance of spotted hyenas (though long‐term spotted hyena data were not available; these would add interesting contrast to our findings). Conditional logistic regression for elephants showed elephants will avoid areas where lions are present in the short term (0–6 h).

Compared to more vulnerable browsers (giraffe, kudu), black rhino and elephants' fear landscapes were modulated more by thermoregulatory and resource constraints than acute risk. Together, these megaherbivores constitute an upper guild, joining body size to foraging ecology in governing how fear propagates through this arid system. This decoupling of megaherbivore behavior from predator influence contributes to system‐level stability, helping to maintain functional continuity of ecosystem processes within which other predator–prey interactions move in dynamic contrast.

### 4.5 A Hierarchical Landscape of Fear

Together, these results revealed a hierarchically organized LOF spanning from risk‐sensitive grazers to risk‐tolerant megaherbivores, with browsers occupying an intermediate, context‐dependent niche. The gradient of fear demonstrates that body size, trophic position, and foraging mode jointly govern how fear propagates through this arid ecosystem and interacts with the environmental constraints therein (Gaynor et al. 2019; Palmer et al. 2022). Temporal and spatial avoidance are thus not binary responses but strategies scaled by trade‐offs and habitat structure (Schooler et al. 2024). In such non‐equilibrium ecosystems, the LOF is characterized by bottom‐up constraints channeling predator–prey interactions into predictable temporal rhythms that maintain and stabilize community function across fluctuating resource landscapes.

## Author Contributions

**John Heydinger:** conceptualization (lead), data curation (lead), formal analysis (lead), funding acquisition (equal), investigation (lead), methodology (lead), resources (equal), writing – original draft (lead), writing – review and editing (lead). **Uakendisa Muzuma:** investigation (equal), project administration (equal), resources (equal), supervision (equal), writing – review and editing (equal). **Tammy Hoth‐Hanssen:** funding acquisition (supporting), project administration (supporting), resources (supporting), writing – review and editing (supporting). **Genevieve Finerty:** conceptualization (supporting), funding acquisition (supporting), project administration (supporting), resources (supporting), writing – review and editing (supporting). **Natalia Borrego:** funding acquisition (supporting), project administration (supporting), resources (supporting), writing – review and editing (supporting). **James Beasley:** conceptualization (supporting), formal analysis (supporting), funding acquisition (equal), methodology (supporting), project administration (supporting), resources (supporting), supervision (lead), writing – review and editing (equal).

## Funding

This study was supported by Future Faculty for Inclusive Research Excellence (FFIRE) Program, Office of Graduate Research, University of Georgia, the Beasley Lab, Warnell School of Forestry, University of Georgia, the University of Minnesota Lion Center, Namibia Ministry of Environment, Forestry and Tourism, the Lion Rangers Progam, Kunene, Namibia, University of Georgia and the US Department of Energy Office of Environmental Management (award number DE‐EM0005228 to the University of Georgia Research Foundation).

## Disclosure

Disclaimer: This manuscript was prepared as an account of work sponsored by an agency of the United States Government. Neither the United States Government nor any agency thereof, nor any of their employees, makes any warranty, express or implied, or assumes any legal liability or responsibility for the accuracy, completeness, or usefulness of any information disclosed, or represents that its use does not infringe privately owned rights. Reference herein to any specific commercial product, process, or service by trade name, trademark, manufacturer, or otherwise does not constitute or imply its endorsement, recommendation, or favoring by the United States Government or any agency thereof. The views and opinions of the authors expressed herein do not necessarily state or reflect those of the United States Government or any agency thereof.

## Ethics Statement

Lions were collared under MEFT supervision by registered veterinarians and research team members, with conservancy management approval. Immobilization followed Namibia Veterinary Council‐approved protocols (Kock and Burroughs 2012; D. Rodenwoldt pers. comm.) using Zoletil, medetomodine, ketamine, and butorphanol, reversed with atipamezole and trexonil as appropriate. Collars were fitted per veterinary recommendations and best practices. No mortalities occurred, and all lions recovered quickly with no observed lingering effects. Max Planck Ethics Council IACUC No. 2021_39/2022_09.

## Conflicts of Interest

The authors declare no conflicts of interest.

## Supporting information


**Data S1:** ece372836‐sup‐0001‐supinfo.docx.

## Data Availability

All the required data are uploaded as Supporting Information. Data remain the property of Namibia's Ministry of Environment, Forestry and Tourism; they can be requested from Uakendisa Muzuma (uakendisa.muzuma@meft.gov.na).
