# Correction to “Bottom‐Up Space Use With Top‐Down Temporal Risk Buffering in Arid Herbivore Communities”

**DOI:** 10.1002/ece3.73143

**Published:** 2026-02-17

Heydinger, J., U. Muzuma, T. Hoth‐Hanssen, G. Finerty, N. Borrego, J. Beasley. 2026. “Bottom‐Up Space Use With Top‐Down Temporal Risk Buffering in Arid Herbivore Communities.” *Ecology and Evolution* 16, no. 1: e72836. https://doi.org/10.1002/ece3.72836.

The middle initials of the fourth and sixth authors are missing in the published article. The complete names of the fourth and sixth authors are as follows:

- Genevieve E. Finerty
- James C. Beasley

Additionally, the institution listed in the seventh affiliation is incorrectly stated as “Aitkin, South Carolina.” It should be “Aiken, South Carolina.”

We apologize for these errors.

