# Supplementary material for: Bottom‐Up Space Use With Top‐Down Temporal Risk Buffering in Arid Herbivore Communities
Source: Ecol Evol. 2026 Jan 4;16(1):e72836. doi: 10.1002/ece3.72836 (PMC12765598; doi:10.1002/ece3.72836)
Supplement: Supplementary file 1 — Data S1: ece372836‐sup‐0001‐supinfo.docx. [file ECE3-16-e72836-s001.docx]

**APPENDIX**

Bottom-up spatial constraints combine with top-down temporal effects to shape herbivore activity patterns in an arid landscape.

Table S1. Summarizes trail camera deployments across six study sites in northwestern Namibia, detailing number of cameras, camera clusters, and waterholes monitored along with total deployment nights, survey area (minimum convex polygon), approximate geographic centerpoint for each deployment, biome type, number of independent detections for the survey, median number of detections per camera cluster, and survey dates for each deployment period. Arrays varied in camera density and spatial coverage, based on camera availability and landscape features (primarily wildlife water availability), to maximize detections of lions and other large carnivores.

| **Trail Camera Deployments** | | | | | | | | | | |
| --- | --- | --- | --- | --- | --- | --- | --- | --- | --- | --- |
| **Array** | **Cameras** | **Clusters** | **Waterholes** | **Nights** | **Area (km^2^)** | **Centerpoint** | **Biome** | **Indep. Detects** | **Med. Cluster Detects** | **Dates** |
| Etendeka | 42 | 23 | 2 | 1633 | 16.89 | S 19.777699 E 13.961591 | Nama karoo | 306 | 11 | May - July 21 |
|  |  |  |  |  |  |  |  |  |  |  |
| Otjiapa | 80 | 23 | 3 | 4085 | 10.65 | S 19.676652 E 13.978432 | Nama karoo | 1905 | 59 | Oct. - Dec. 21 |
|  |  |  |  |  |  |  |  |  |  |  |
| Omirembue | 81 | 26 | 2 | 4203 | 4.92 | S 19.721752 E 14.118704 | Nama karoo | 1944 | 54 | Dec. 21 - Mar. 22 |
|  |  |  |  |  |  |  |  |  |  |  |
| Hobatere | 76 | 25 | 1 | 1498 | 1.73 | S 19.367357 E 14.34207 | Western highlands | 639 | 15 | July - Sept. 22 |
|  |  |  |  |  |  |  |  |  |  |  |
| Anabeb/Palmwag | 68 | 17 | 7 | 1378 | 20.86 | S 19.563532 E 13.822824 | Nama karoo | 297 | 9 | Jan. 23 |
|  |  |  |  |  |  |  |  |  |  |  |
| Ugab | 27 | 11 | 8 | 525 | 51.92 | S 21.037843 E 14.531782 | Namib desert | 177 | 11 | June - Aug. 23 |

Figure S1. Map of Etendeka trail camera array, Etendeka Concession, northwest Namibia May-July 2021 (Figure 1, inset #1). Red dots indicate camera positions; blue icons are waterholes.


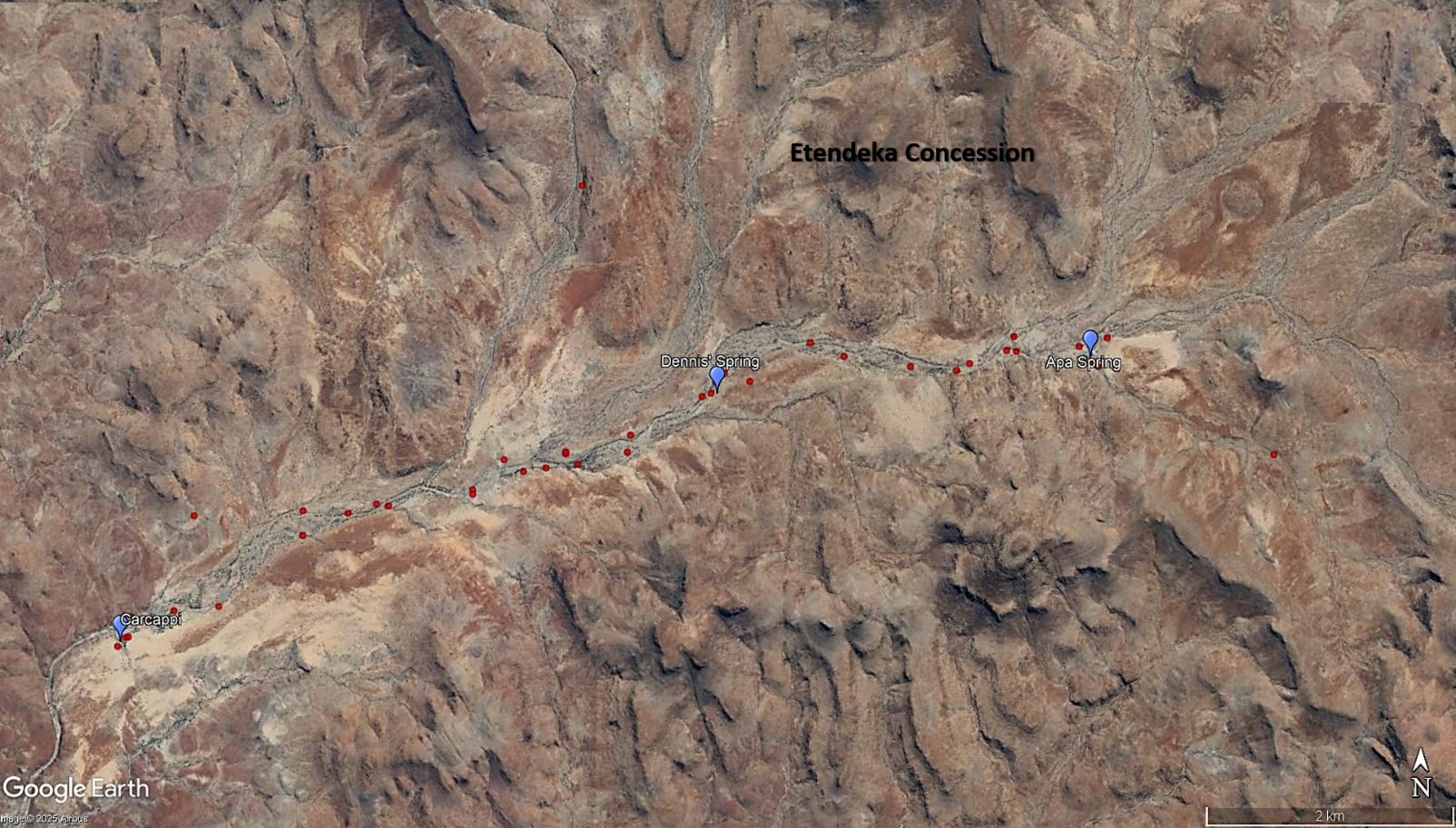


Figure S2. Map of Otjiapa trail camera array, Anabeb and Omatendeka conservancies, Etendeka Concession northwest Namibia October-December 2021 (Figure 1, inset #2). Red dots indicate camera positions; blue icons are waterholes.


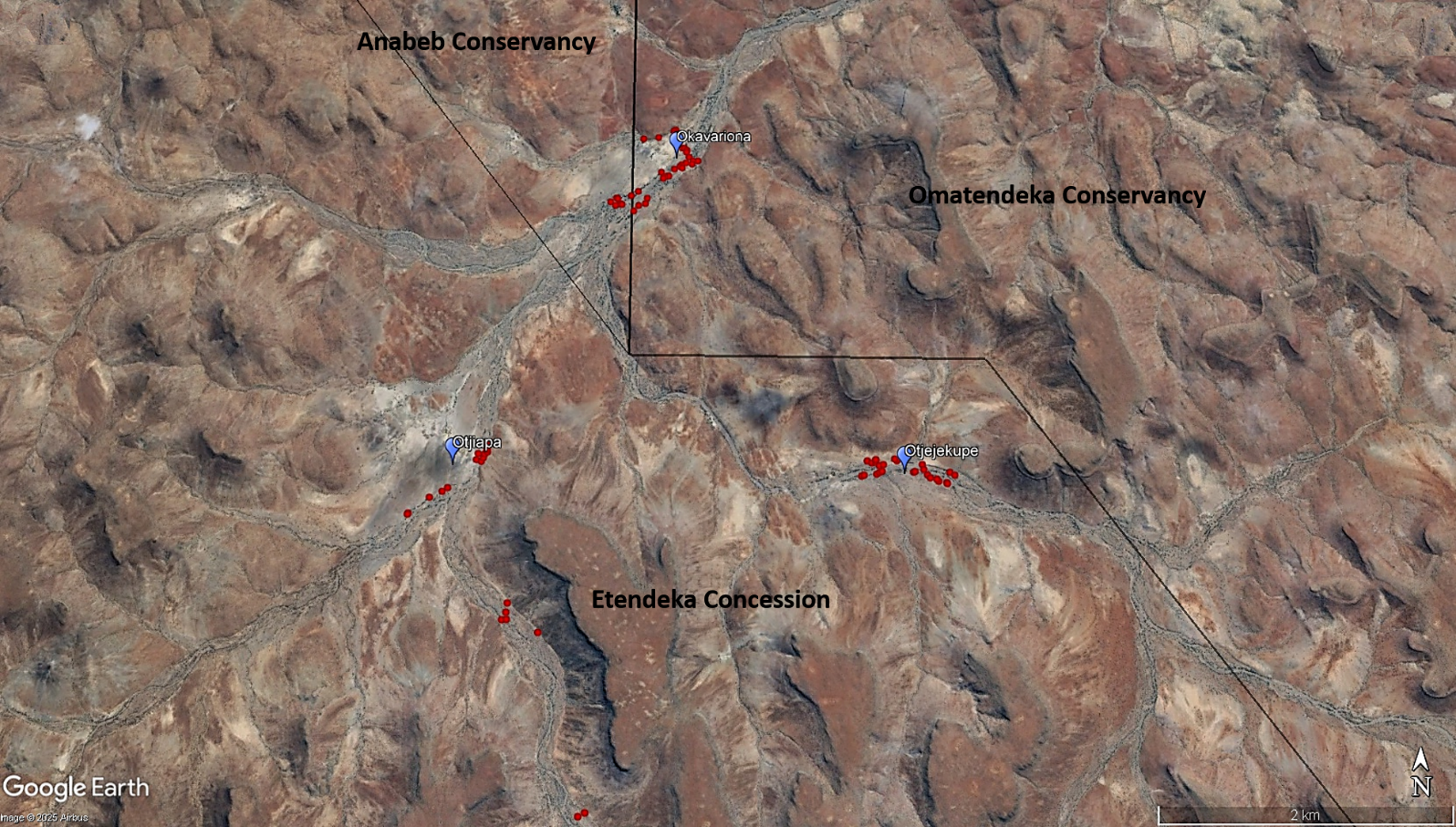


Figure S3. Map of Omirembue trail camera array, Omatendeka Conservancy, northwest Namibia December 2021-February 2022 (Figure 1, inset #3). Red dots indicate camera positions; blue icons are waterholes.


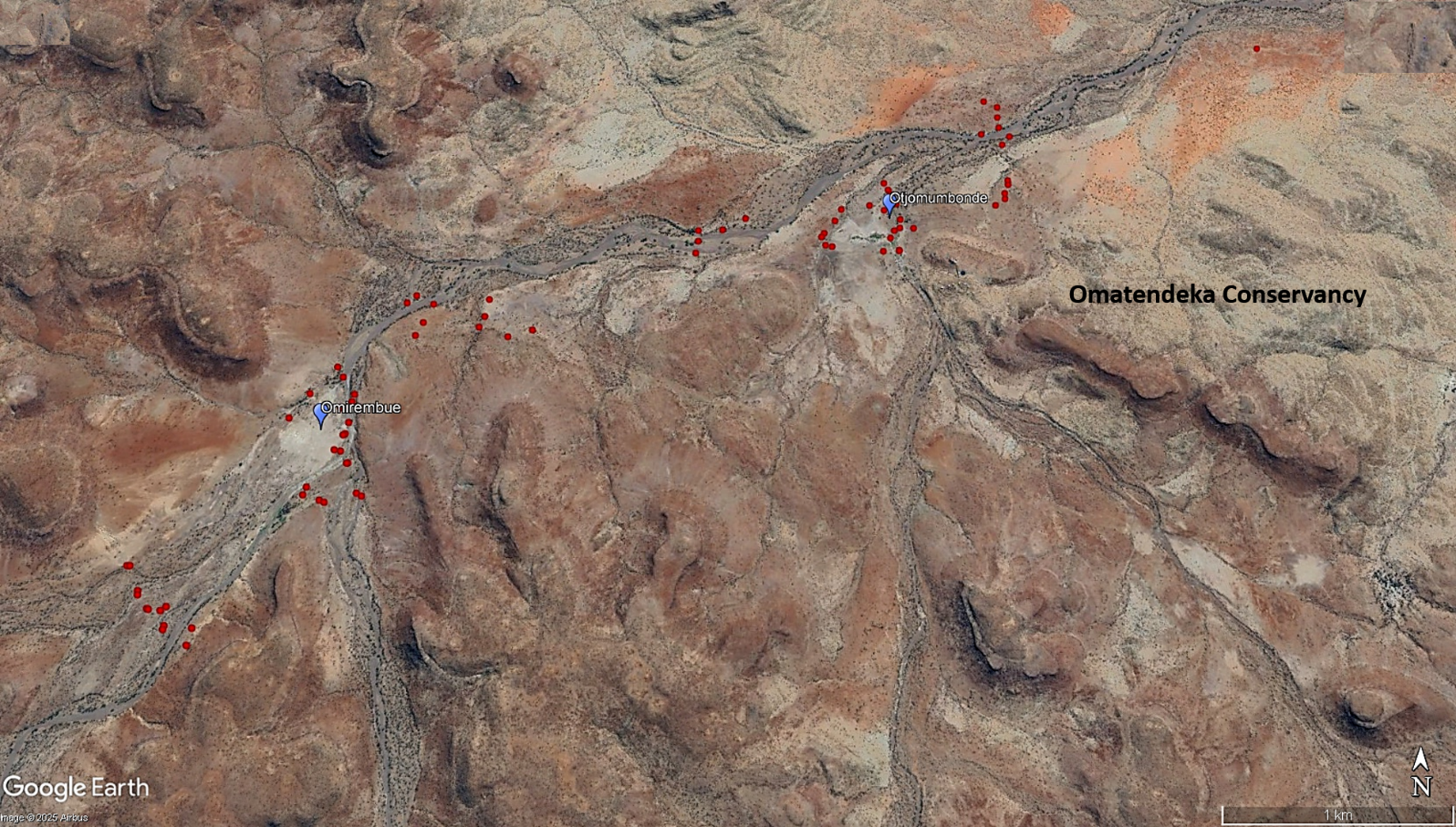


Figure S4. Map of Hobatere trail camera array, Hobatere Concession, northwest Namibia July-September 2022 (Figure 1, inset #4). Red dots indicate camera positions; blue icons are waterholes.


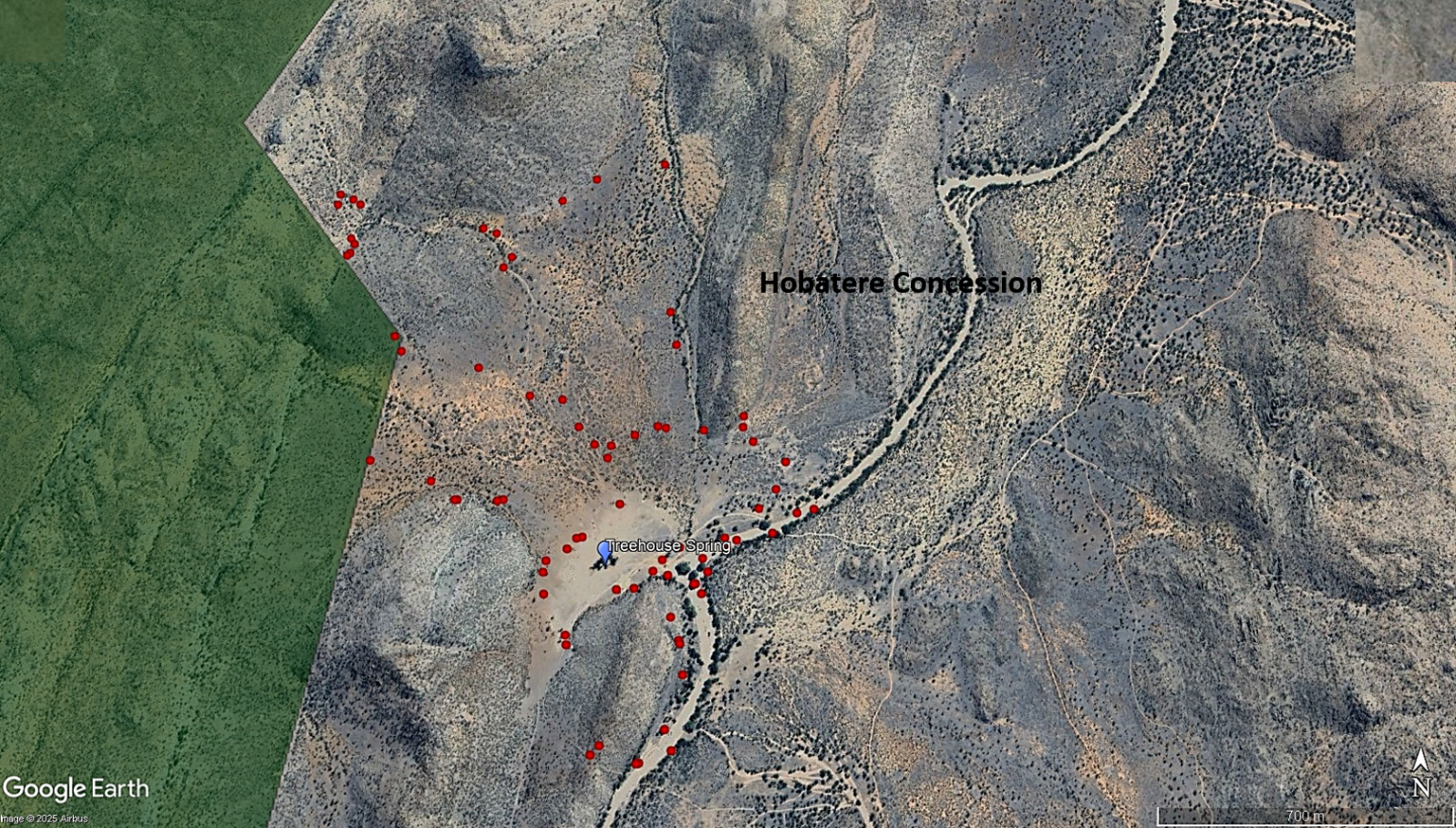


Figure S5.1. Map of Anabeb/Palmwag trail camera array, Palmwag Concession, northwest Namibia January 2023 (Figure 1, inset #5). Red dots indicate camera positions; blue icons are waterholes.


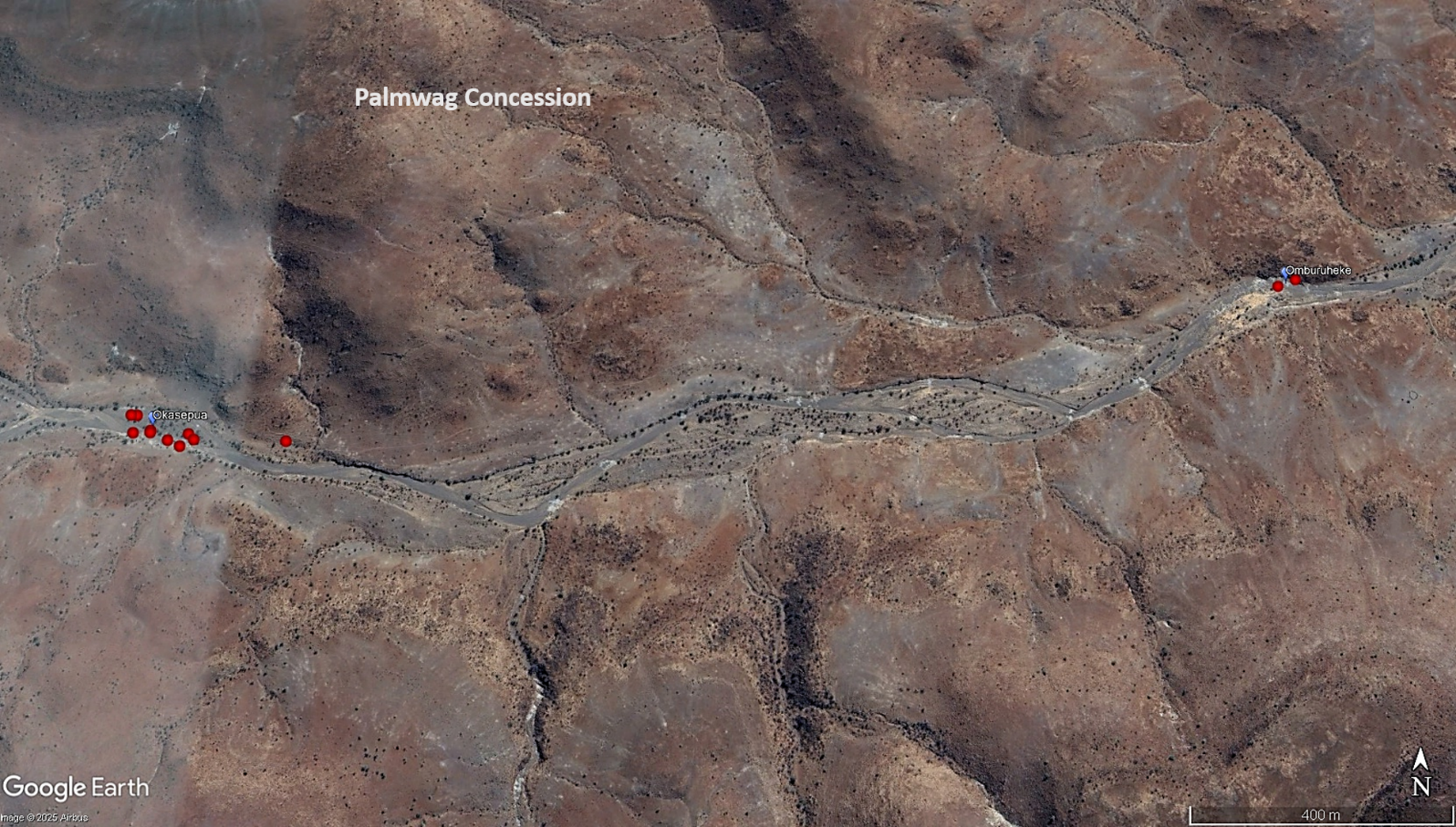


Figure S5.2. Map of Anabeb/Palmwag trail camera array, Palmwag Concession, northwest Namibia January 2023 (Figure 1, inset #5). Red dots indicate camera positions; blue icons are waterholes.


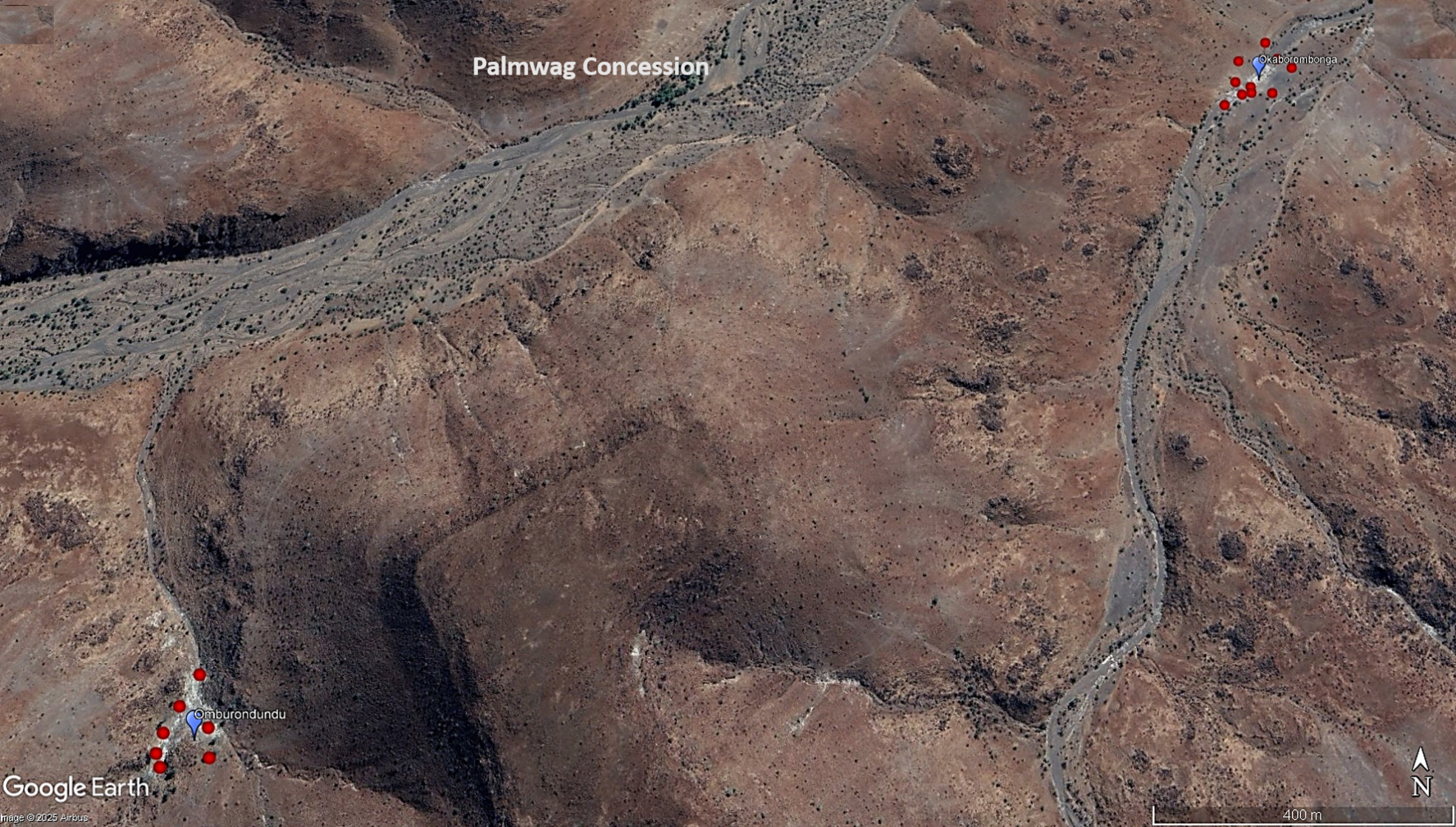


Figure S5.3. Map of Anabeb/Palmwag trail camera array, Anabeb Conserancy, northwest Namibia January 2023 (Figure 1, inset #5). Red dots indicate camera positions; blue icons are waterholes.


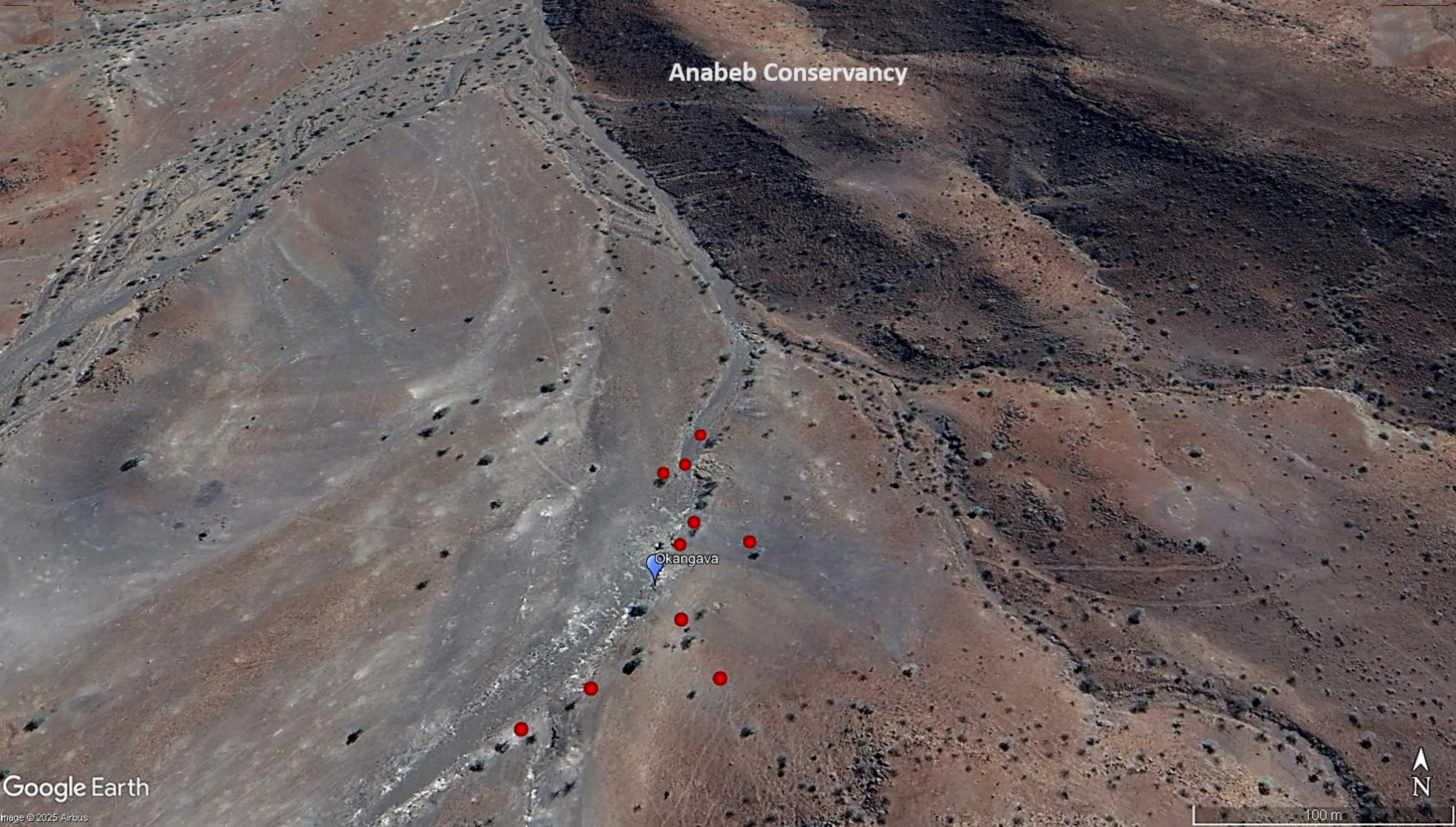


Figure S5.4. Map of Anabeb/Palmwag trail camera array, Anabe Conservancy, northwest Namibia January 2023 (Figure 1, inset #5). Red dots indicate camera positions; blue icons are waterholes.


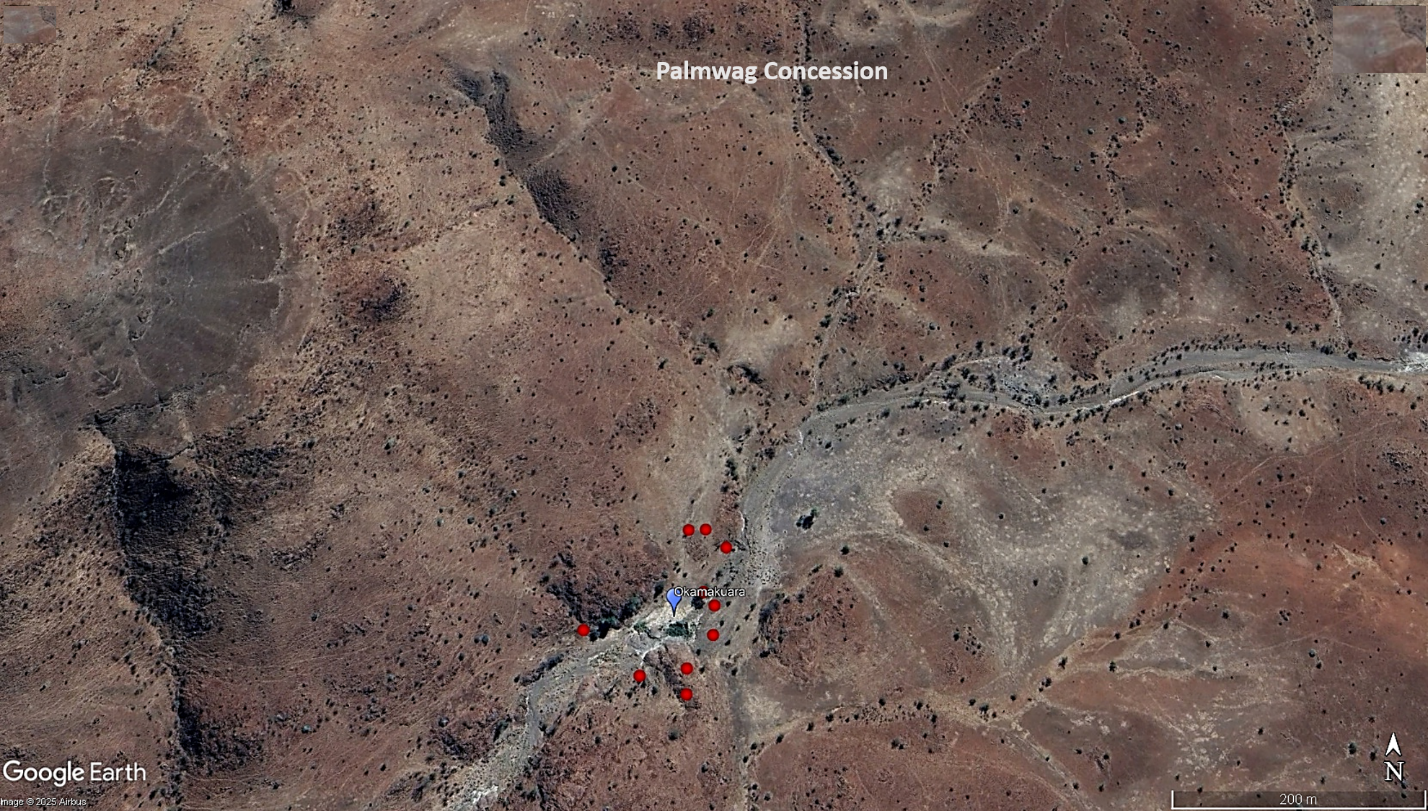


Figure S5.5 Map of Anabeb/Palmwag trail camera array, Anabeb Conservancy, northwest Namibia January 2023 (Figure 1, inset #5). Red dots indicate camera positions; blue icons are waterholes.


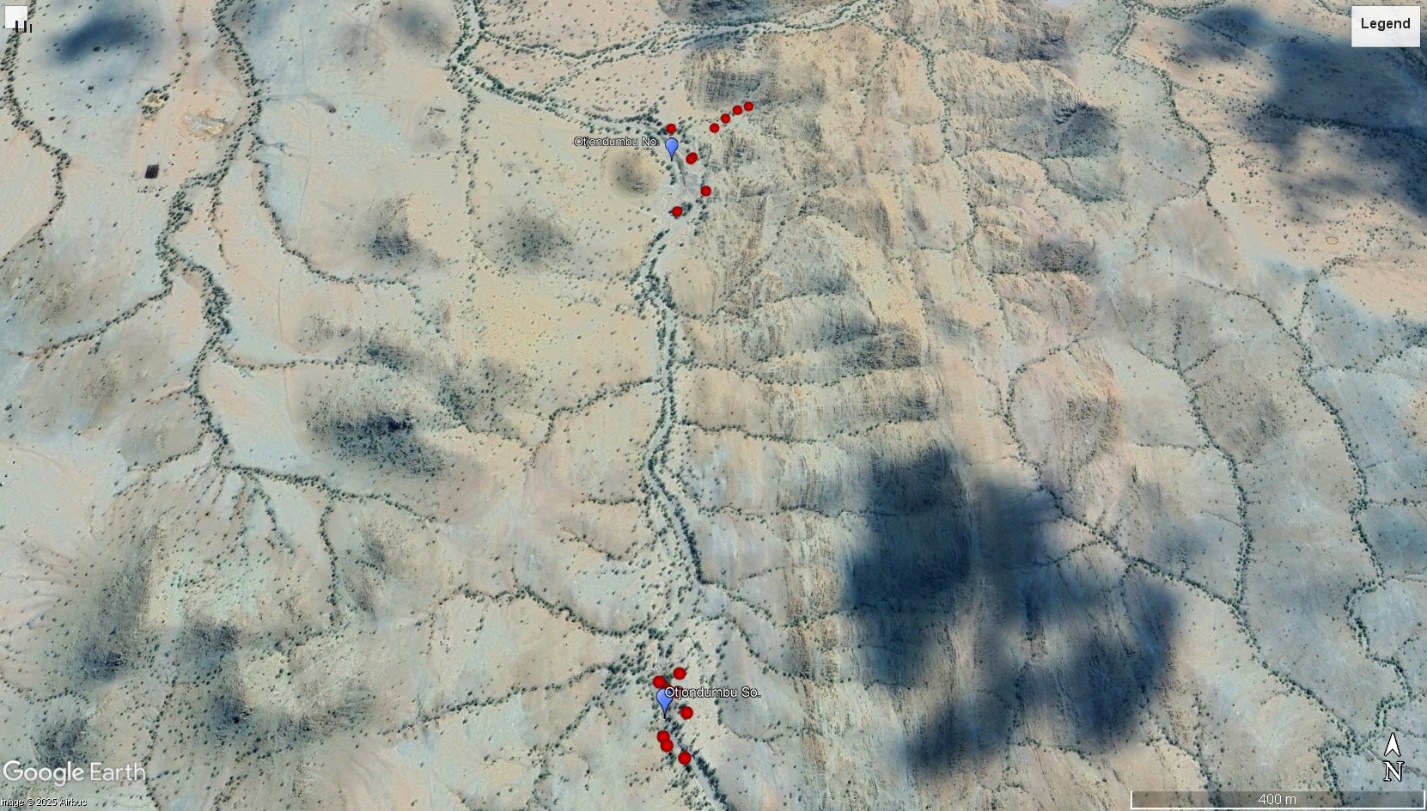


Figure S6.1. Map of Ugab trail camera array, Tsiseb Conservancy, northwest Namibia January 2023 (Figure 1, inset #6). Red dots indicate camera positions; blue icons are waterholes.


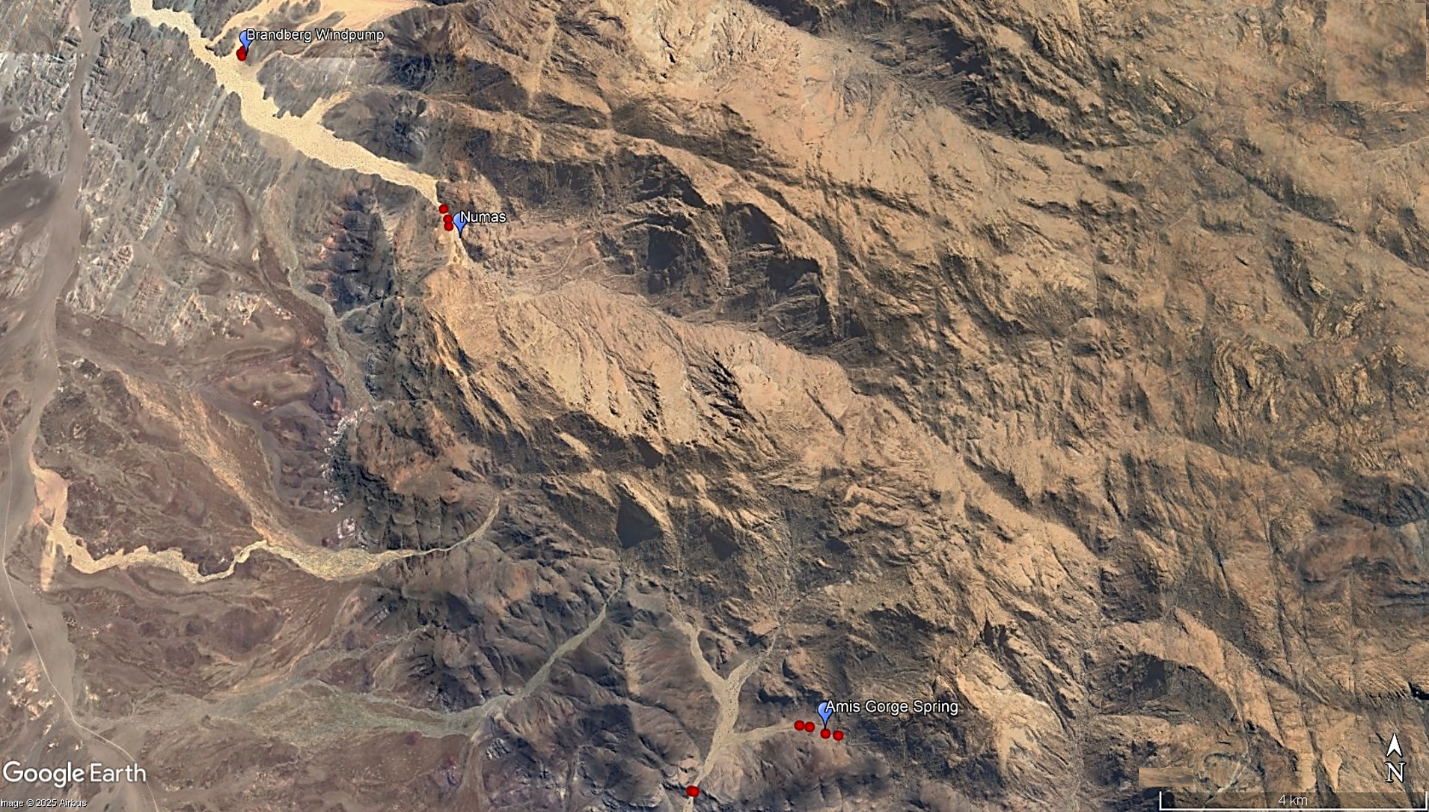


Figure S6.2. Map of Ugab trail camera array, Doro !Nawas Conservancy, northwest Namibia January 2023 (Figure 1, inset #6). Red dots indicate camera positions; blue icons are waterholes.


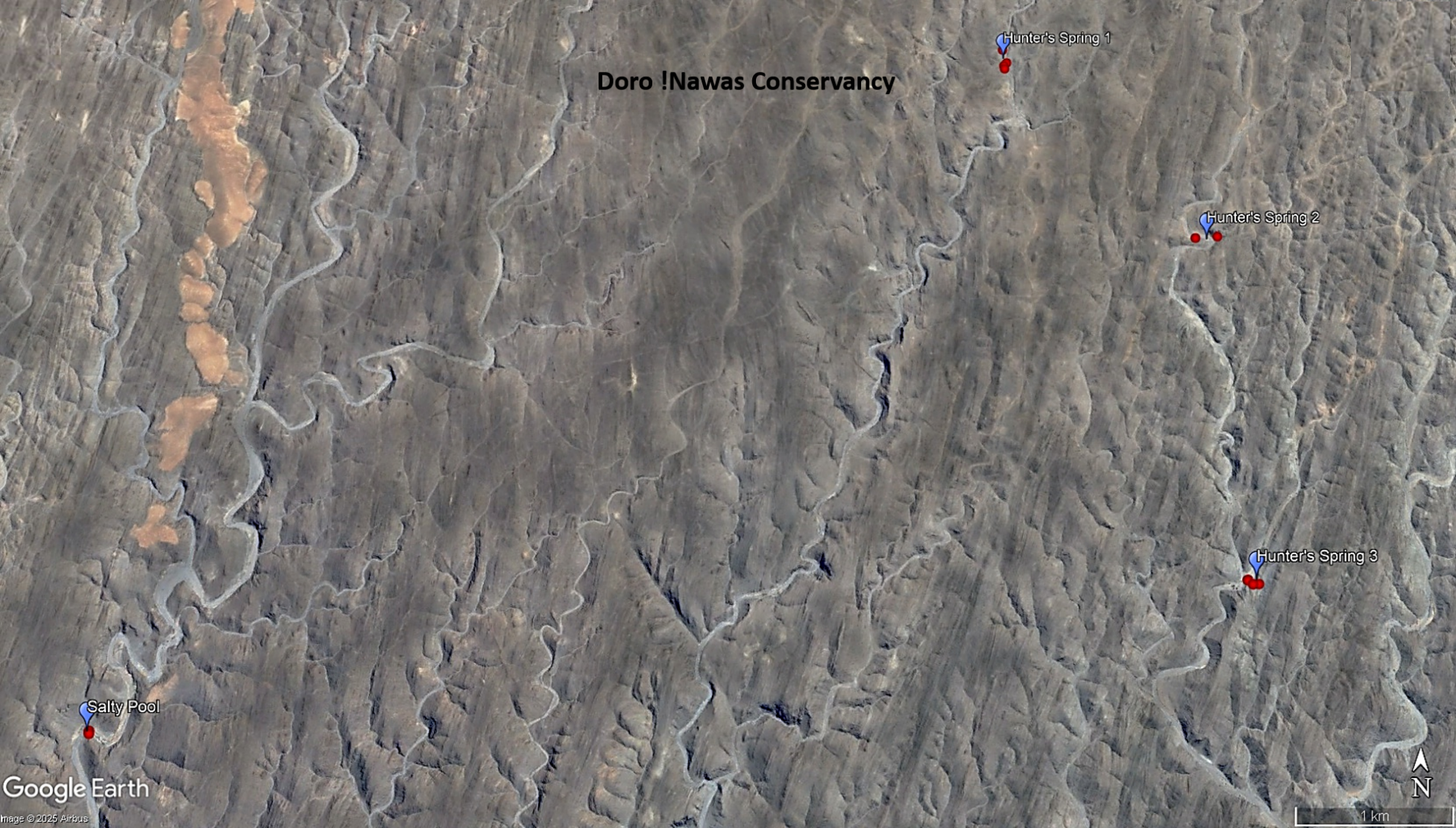


Figure S6.3. Map of Ugab trail camera array, Sorris-Sorris Conservancy, northwest Namibia January 2023 (Figure 1, inset #6). Red dots indicate camera positions; blue icons are waterholes.


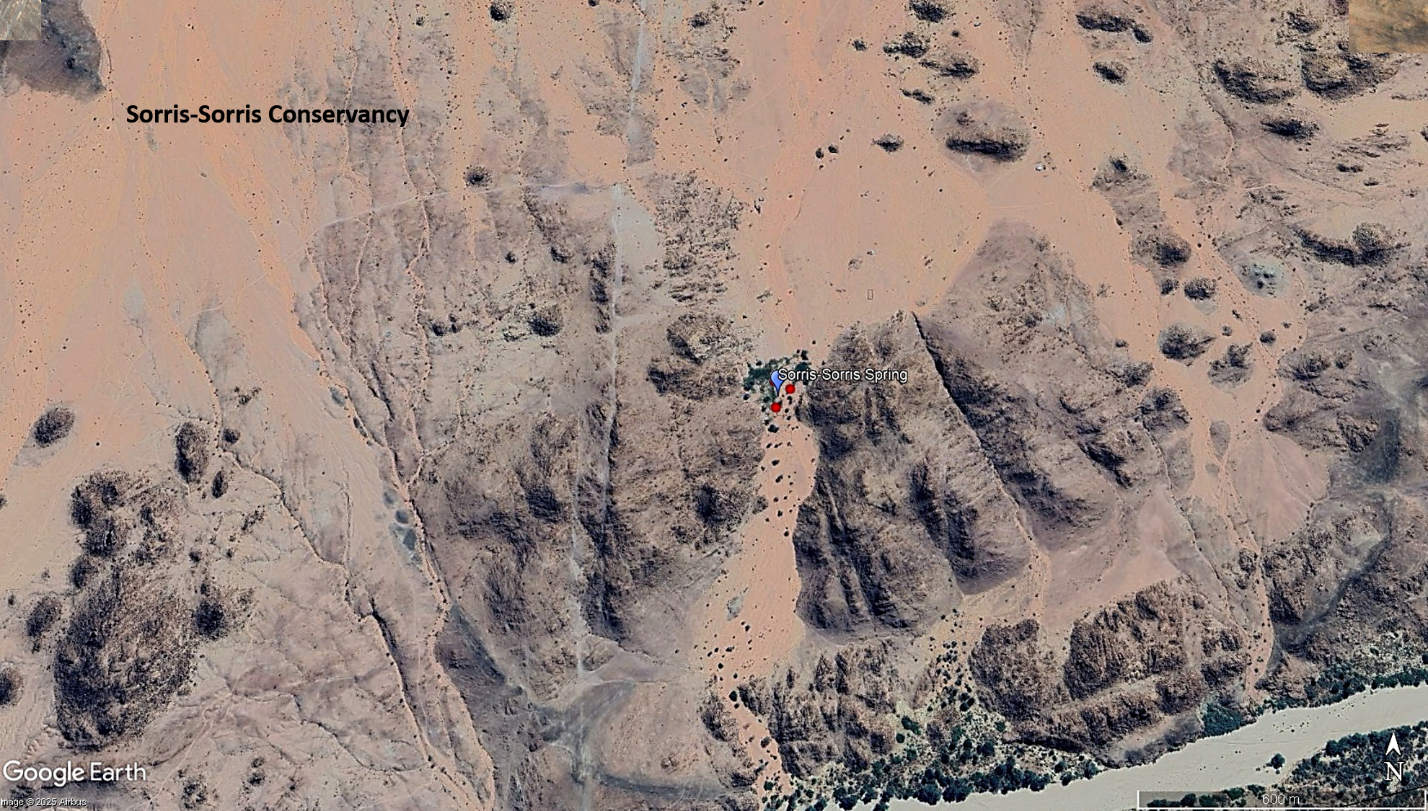


Table S2. Data dictionary for environmental and biotic covariates assessed as drivers of herbivore species detection on camera trap deployment across six areas in northwest Namibia. Covariates assessed, data type, unit resolution, data distribution (untransformed), transformation for analysis, and data source. Vegetation cover, amount of woody and green vegetation at each site **(**1: no vegetation; 1.5: sparse grass, no trees; 2: scattered, sparsely vegetated trees with little grass; 2.5 scattered leafy trees with occasional dense stands of grass or reeds; 3: stands of trees partially obstructing visibility; 3.5: thick stands of tree consistently obstructing visibility). Visibility, line-of-site distance for ≥ 51% of the viewshed from camera position (1: 0-5 meters; 2: 5-10 meters; 3: 10-30 meters; 4: 30+ meters). Environmental covariate details: water proximity, Euclidean distance from camera to nearest water source (0: ≤ 30 meters with water visible; 1: 0-100 meters, no water visible; 2: 100-500 meters from water; 3: ≥ 500 meters). Human use intensity, frequency human of foot and vehicle use (0: minimal/no use, site accessible by foot; 1: infrequent use, accessible by 4x4, or foot; 2: moderate Ranger, tourism, or livestock use of nearby water; 3: frequent Ranger, tourism, or use livestock use of nearby water and site; 4: daily predictable use). Biotic covariate details: predator presence (lion *Panthera leo*, spotted hyena *Crocuta crocuta*) recorded as daily presence/absence (≤ 24 hours prior to herbivore detection); detection rate, species detections per 100 camera-nights per cluster (Cusack et al. 2017); long-term lion activity derived from GPS collar data quantified as lion kernel density estimates (KDE) with scoring that weighted core range (50% KDE = 2) and home range (95% KDE = 1) use based on the number of collared lions’ ranges overlapping with a camera cluster (________ unpublished data).

| **Variable** | **Type** | **Units** | **Temporal Resolution** | **Raw Distribution** | **Transformation** | **Source** |
| --- | --- | --- | --- | --- | --- | --- |
| Vegetation cover | Numeric | 0.5 | Survey | 1–3.5 | log(1+ *x*) | Field assessment |
| Visibility | Numeric | 1 | Survey | 1–4 | log(1+ *x*) | Field assessment |
| Water proximity | Numeric | 1 | Survey | 0–3 | log(1+ *x*) | Remote sensing/Field assessment |
| Human Use Intensity | Numeric | 1 | Survey | 0–4 | log(1+ *x*) | Expert opinion |
| Temperature | Numeric | °C | Detection time | 10–38 | log(1+ *x*) | weatherspark.com |
| Elevation | Numeric | meters | Survey | 361–1219 | log(1+ *x*) | elevatr (Hollister et al. 2025) |
| Latitude | Numeric | degrees south | Survey | 19.36–21.21 | z-score | Field GPS point |
| Longitude | Numeric | degrees east | Survey | 13.74–14.79 | z-score | Field GPS point |
| Dry season progression | Numeric | days | Daily | -9–297 | z-score | Camera logs |
| Lunar illumination | Percentage | 0.01 | Daily | 0–1 | log(1+ *x*) | suncalc (Thieurmel 2024) |
| NDVI | Percentage | 0.01 | Survey | 0–1 | z-score | AppEEARS (nasa.gov) |
| Management type | Categorical | Conservancy (0) / Concession (1) | Survey | 0/1 | none | NACSO 2024 |
| Predator presence | Binary | Y/N | Daily | 0/1 | none | Camera images |
| Detection rates | Numeric | 0.01 | Survey | 0–29.5775 | log(1+ *x*) | Camera images |
| Lion long-term activity | Numeric | 1 | Survey | 0-2 | log(1+ *x*) | Heydinger, unpublished data |

Sources:

Cusack, J.J., Dickman, A.J., Kalyahe, M., Rowcliffe, J.M., Carbone, C., MacDonald, D.W., Coulson, T., 2017. Revealing kleptoparasitic and predatory tendencies in an African mammal community using camera traps: a comparison of spatiotemporal approaches. Oikos 126, 812–822.

Hollister, J., Shah, T., Nowosad, J., Robitaille, A., Beck, M., Johnson, M. 2025. Elevatr: Access Elevation Data from Various APIs. R package version 0.99.1.

https://cran.r-project.org/web/packages/elevatr/citation.html (Accessed 9 April 2025).

(NACSO) Namibia Association of CBNRM Support Organizations, 2023. The State of Community Conservation in Namibia (Annual Report 2022). Windhoek.

NASA, 2025. Application for Extracting and Exploring Analysis Ready Sample (AppEEARS). https://appeears.earthdatacloud.nasa.gov/ (Accessed 11 April 2025).

Thieurmel B, Elmarhraoui A, Thieurmel MB. 2024. Package ‘suncalc’. R package version 0.5. https://cran.r-project.org/web/packages/suncalc/suncalc.pdf/ (Accessed 1 March 2024).

Weatherspark. 2024. Weatherspark: the weather year round anywhere on Earth. URL: https://weatherspark.com/y/74187/Average-Weather-in-Opuwo-Namibia-Year-Round (Accessed 15 February 2024).

Table S3. Model comparison results for Bayesian logistic regression models of herbivore detection probability at camera clusters in northwest Namibia. Four model types were compared: posthoc (parsimonious model based on full model performance and ecological relevance), full (weak priors), full (horseshoe priors), and null (intercept-only). Model performance was assessed using expected log predictive density (ELPD), estimated via leave-one-out cross-validation (LOO). ELPD, expected log point wise predictive density; SE ELPD, standard error of ELPD; Bayesian R^2^, posterior mean estimate of R^2^, r^2^ CrI (2.5%) / r^2^ CrI (97.5), lower and upper bounds of the 95% credible interval; Stacking Weight, model weight from Bayesian model averaging based on LOO indicating relative model weight across candidates based on predictive performance.

| **Model** | ELPD | ELPD SE | Bayes R^2^ | R^2^ SE | R^2^ CrI (2.5%) | R^2^ CrI (97.5%) | Model Weight |
| --- | --- | --- | --- | --- | --- | --- | --- |
| **Gemsbok** |  |  |  |  |  |  |  |
| Posthoc | -150.6 | 11.0 | 0.412 | 0.034 | 0.346 | 0.477 | 0.812 |
| Full (weak priors) | -158.0 | 12.1 | 0.455 | 0.031 | 0.388 | 0.511 | 0.000 |
| Full (horsehoe) | -151.9 | 9.6 | 0.338 | 0.043 | 0.252 | 0.418 | 0.007 |
| Null | -166.0 | 8.9 | 0.261 | 0.043 | 0.173 | 0.339 | 0.181 |
|  |  |  |  |  |  |  |  |
| **Giraffe** |  |  |  |  |  |  |  |
| Posthoc | -191.5 | 12.7 | 0.391 | 0.029 | 0.331 | 0.445 | 1.000 |
| Full (weak priors) | -199.6 | 13.6 | 0.423 | 0.028 | 0.367 | 0.475 | 0.000 |
| Full (horsehoe) | -196.5 | 12.0 | 0.355 | 0.034 | 0.284 | 0.418 | 0.000 |
| Null | -240.3 | 11.5 | 0.205 | 0.036 | 0.133 | 0.273 | 0.000 |
|  |  |  |  |  |  |  |  |
| **Kudu** |  |  |  |  |  |  |  |
| Posthoc | -138.2 | 9.2 | 0.404 | 0.042 | 0.315 | 0.479 | 1.000 |
| Full (weak priors) | -147.2 | 10.5 | 0.436 | 0.037 | 0.358 | 0.502 | 0.000 |
| Full (horsehoe) | -145.9 | 9.1 | 0.372 | 0.049 | 0.266 | 0.460 | 0.000 |
| Null | -177.7 | 7.2 | 0.158 | 0.048 | 0.062 | 0.247 | 0.000 |
|  |  |  |  |  |  |  |  |
| **Mtn Zebra** |  |  |  |  |  |  |  |
| Posthoc | -364.5 | 22.3 | 0.371 | 0.026 | 0.319 | 0.419 | 0.638 |
| Full (weak priors) | -366.9 | 22.8 | 0.392 | 0.024 | 0.344 | 0.437 | 0.355 |
| Full (horsehoe) | -368.0 | 22.0 | 0.365 | 0.028 | 0.308 | 0.416 | 0.000 |
| Null | -476.1 | 26.0 | 0.199 | 0.027 | 0.145 | 0.252 | 0.007 |
|  |  |  |  |  |  |  |  |
| **Springbok** |  |  |  |  |  |  |  |
| Posthoc | -268.1 | 17.7 | 0.315 | 0.033 | 0.248 | 0.376 | 0.914 |
| Full (weak priors) | -295.0 | 21.8 | 0.353 | 0.029 | 0.293 | 0.407 | 0.000 |
| Full (horsehoe) | -276.5 | 18.1 | 0.305 | 0.034 | 0.235 | 0.368 | 0.000 |
| Null | -310.2 | 18.3 | 0.220 | 0.032 | 0.153 | 0.280 | 0.086 |
|  |  |  |  |  |  |  |  |
| **Black rhino** |  |  |  |  |  |  |  |
| Posthoc | -72.9 | 7.4 | 0.344 | 0.046 | 0.248 | 0.429 | 0.794 |
| Full (weak priors) | -89.0 | 9.6 | 0.383 | 0.041 | 0.300 | 0.462 | 0.000 |
| Full (horsehoe) | -78.1 | 5.1 | 0.200 | 0.074 | 0.053 | 0.336 | 0.000 |
| Null | -78.9 | 4.2 | 0.143 | 0.068 | 0.019 | 0.275 | 0.206 |
|  |  |  |  |  |  |  |  |
| **Elephant** |  |  |  |  |  |  |  |
| Posthoc | -154.3 | 11.7 | 0.406 | 0.032 | 0.341 | 0.467 | 0.972 |
| Full (weak priors) | -160.6 | 12.8 | 0.429 | 0.030 | 0.369 | 0.487 | 0.005 |
| Full (horsehoe) | -157.1 | 11.0 | 0.379 | 0.037 | 0.305 | 0.448 | 0.003 |
| Null | -196.4 | 10.5 | 0.196 | 0.038 | 0.119 | 0.267 | 0.020 |

**GEMSBOK**

Table S4. Posterior summaries from the posthoc Bayesian regression model with weak priors evaluating covariates influencing gemsbok (*Oryx gazella*) detection probability at camera clusters. Continuous variables log-transformed using log1p to reduce skew. Spatial and temporal predictors were standardized (z-score scaled). Columns include posterior mean estimates, standard errors and 95% credible intervals. Parameters that do not overlap zero are interpreted as having strong evidence of effect.

| **Parameter** | **Estimate** | **Est. Error** | **CrI (2.5%)** | **CrI (97.5%)** |
| --- | --- | --- | --- | --- |
| Intercept | 4.319 | 5.889 | -7.102 | 15.938 |
| Visibility | 1.186 | 1.677 | -2.110 | 4.457 |
| Site Cover | 0.091 | 1.886 | -3.621 | 3.765 |
| Temperature | -1.165 | 0.882 | -2.916 | 0.540 |
| Luminosity | 0.080 | 1.826 | -3.501 | 3.665 |
| Dry season | -0.252 | 1.599 | -3.320 | 2.912 |
| NDVI | 0.070 | 0.407 | -0.805 | 0.808 |
| Concession/Cons | 0.577 | 1.911 | -3.171 | 4.338 |
| Lion present | -0.440 | 1.469 | -3.255 | 2.536 |
| Sp hyena daily presence | -0.158 | 1.089 | -2.203 | 2.069 |
| Lion detects | -0.048 | 1.606 | -3.191 | 3.123 |
| Sp hyena detects | 0.086 | 1.738 | -3.290 | 3.493 |
| Lion long-term | -0.780 | 1.390 | -3.483 | 1.936 |
| Bl rhino detects | 0.343 | 0.623 | -0.877 | 1.595 |
| BF impala detects | 0.797 | 0.523 | -0.221 | 1.862 |
| Giraffe detects | 0.287 | 0.386 | -0.476 | 1.051 |
| Mtn zebra detects | 0.660 | 0.343 | 0.026 | 1.387 |
| Springbok detects | 0.038 | 1.057 | -2.069 | 2.086 |
| Visibility x Lion detects | 0.334 | 1.161 | -1.963 | 2.641 |
| Visibility x Sp hyena detects | -0.028 | 1.112 | -2.243 | 2.138 |
| Site Cover x Springbok detects | -0.772 | 0.961 | -2.663 | 1.112 |
| Site Cover x Sp hyena detects | 0.747 | 1.414 | -1.999 | 3.577 |
| Site Cover x Lion long term | -0.784 | 0.996 | -2.773 | 1.158 |
| Luminosity x Lion present | -0.788 | 1.918 | -4.562 | 2.952 |
| Luminosity x Sp hyena present | 0.611 | 0.971 | -1.296 | 2.508 |
| Luminosity x Lion long term | 0.286 | 0.660 | -1.008 | 1.587 |
| Concession/Cons x Lion present | -1.538 | 1.767 | -4.977 | 1.938 |
| Concession/Cons x Sp hyena present | 1.227 | 1.586 | -1.749 | 4.481 |
| Concession/Cons x Lion detects | -0.072 | 1.082 | -2.168 | 2.086 |
| Concession/Cons x Lion long term | 0.767 | 0.745 | -0.684 | 2.240 |
| Dry season x Lion present | 1.716 | 1.735 | -1.644 | 5.172 |
| Dry season x Sp hyena present | 1.649 | 0.823 | 0.109 | 3.339 |
| Dry season x Lion long term | 1.131 | 0.592 | 0.038 | 2.358 |
|  |  |  |  |  |
| **Random Effects** (Intercept) |  |  |  |  |
| Camera Cluster | 0.37 | 0.26 | 0.01 | 0.96 |
| Survey | 5.13 | 1.45 | 2.76 | 8.4 |

**GEMSBOK BAYESIAN POSTHOC PLOTS**

Figure S7. Marginal effects plots from the Bayesian posthoc model showing predicted detection probability of gemsbok (*Oryx gazella*) at camera clusters across continuous covariates in northwest Namibia. Species and environmental predictors include black-faced impala (*Aepyceros melampus petersi*) detections, black rhino (*Diceros bicornis*) detections, dry season progression, southern giraffe (*Giraffa giraffa*) detections, lion (*Panthera leo*) detections, lion long-term activity, mountain zebra (*Equus zebra*) detections, NDVI (standardized), camera cluster vegetation cover, spotted hyena (*Crocuta crocuta*) detections, springbok (*antidorcas marsupialis*) detections, temperature (modelled), and camera cluster visibility. Shaded ribbons represent 95% credible intervals.


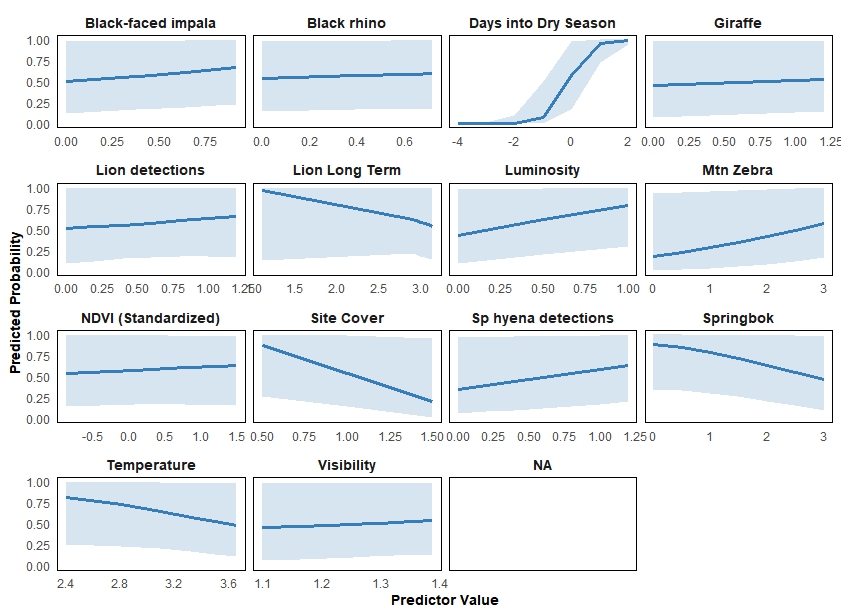


Figure S8. Marginal predicted detection probabilities for gemsbok (*Oryx gazella*) across binary predictors in the Bayesian posthoc model at camera clusters, using camera trap data from northwest Namibia. Panels show estimated probabilities by (a) land tenure (communal conservancy vs. government concession), (b) lion (*Panthera leo*) daily presence, and (c) spotted hyena (*Crocuta crocuta*) daily presence, with bars representing posterior means and lines showing 95% credible intervals.


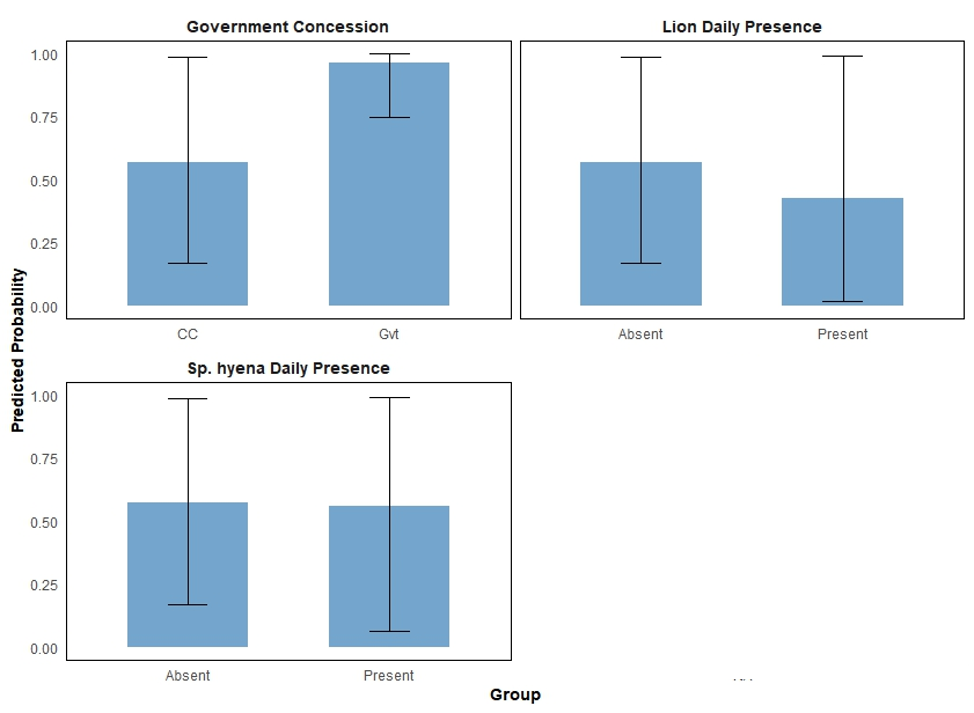


Figure S9. Interaction plots showing predicted detection probability of gemsbok (*Oryx gazella*) in response to interactions between (top left) camera cluster vegetative cover and detections of spotted hyena (*Crocuta crocuta*), (top right) camera cluster vegetative cover and detections of springbok (*antidorcas marsupialis*), (bottom left) camera cluster visibility and detections of lion (*Panthera leo*), and (bottom right) camera cluster visibility and detections of spotted hyena. Lines show mean predicted values from the Bayesian posthoc model, with shaded ribbons showing 95% credible intervals, based on camera trap data from northwest Namibia.


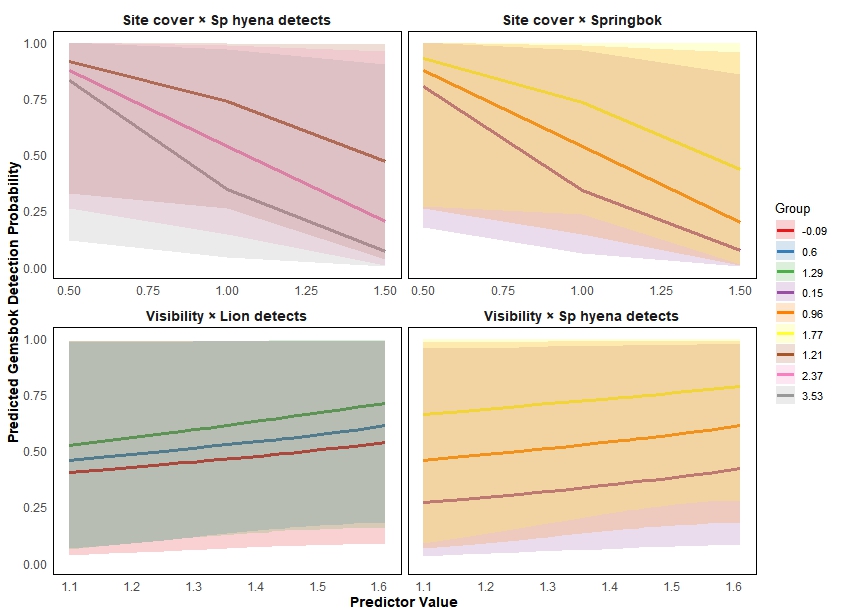


Figure S10. Interaction plots showing predicted detection probability of gemsbok (*Oryx gazella*) in response to combinations of (top left) luminosity and long-term lion (*Panthera leo*) presence, (top right) luminosity and binary lion presence, (bottom left) luminosity and spotted hyena (*Crocuta crocuta*) detections, and (bottom right) site vegetative cover and long-term lion activity. Predictions are based on the Bayesian posthoc model using camera trap data from northwest Namibia.


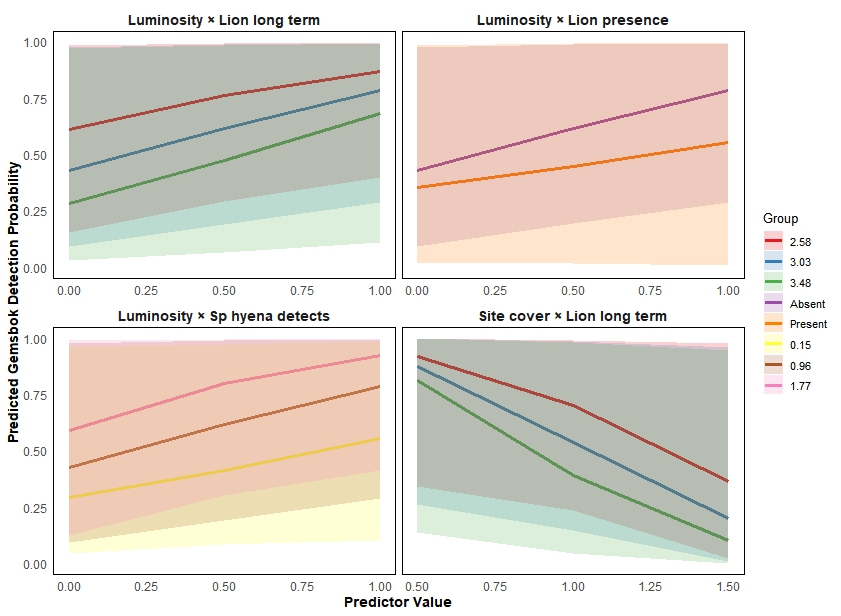


Figure S11. Interaction plots from the Bayesian posthoc model showing the effect of seasonal progression and predator presence on the detection probability of gemsbok (*Oryx gazella*) in northwest Namibia. Plots illustrate predicted detection in response to (left) days into the dry season and daily lion (*Panthera leo*) presence, (center) days into the dry season and long-term lion presence, and (right) days into the dry season and daily presence of spotted hyena (*Crocuta crocuta*).


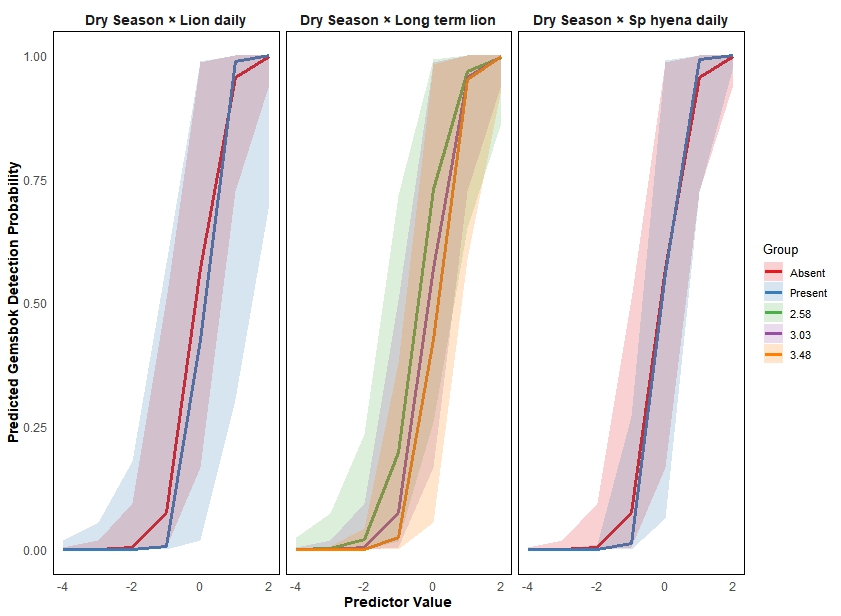


Figure S12. Interaction plots from the Bayesian posthoc model showing predicted detection probability of gemsbok (*Oryx gazella*) in response to interactions between land tenure (communal conservancies vs. government concessions) and predator presence at camera clusters. Panels show interactions between (top left) spotted hyena (*Crocuta crocuta*), (top right) lion (*Panthera leo*) daily presence, (bottom left) lion detection rate, (d) long-term lion activity, with land tenure. All predictions are derived from camera trap data collected in northwest Namibia.


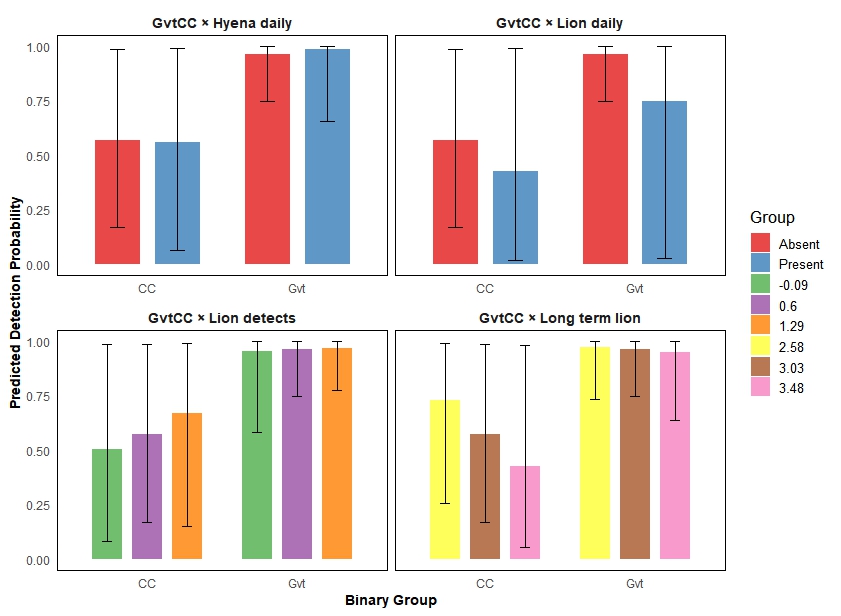


Figure S13. Comparison of predicted probabilities from the top-ranked Bayesian posthoc model versus the model-averaged predictions from Bayesian stacking for gemsbok (*Oryx gazella*), based on camera trap detections in northwest Namibia. The strong linear correlation reflects excellent agreement between top model and model-averaged predictions.


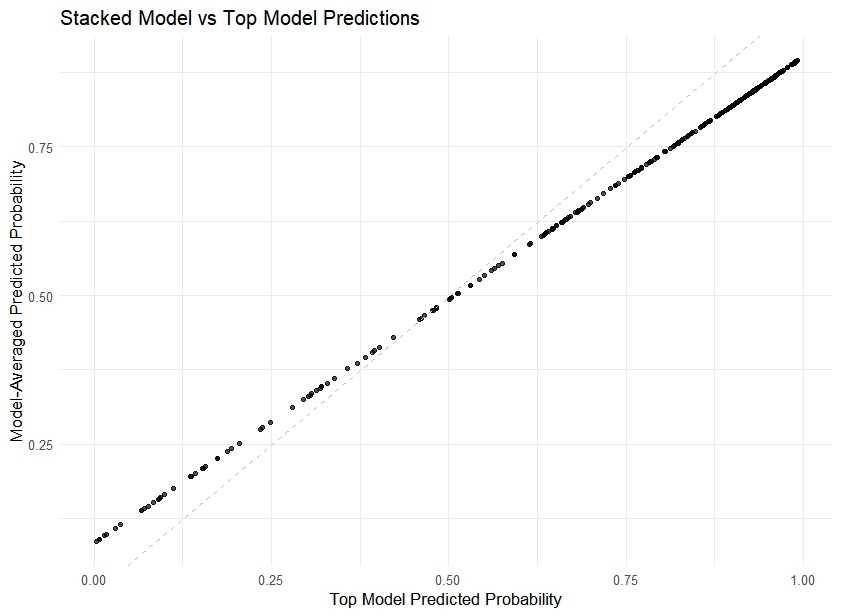


Figure S14. Posterior predictive check (pp_check) plot showing the distribution of predicted versus observed detection probabilities from the Bayesian posthoc model for gemsbok (*Oryx gazella*) based on camera trap survey data from northwest Namibia. Light blue lines show model-simulated draws; dark line shows observed distribution.


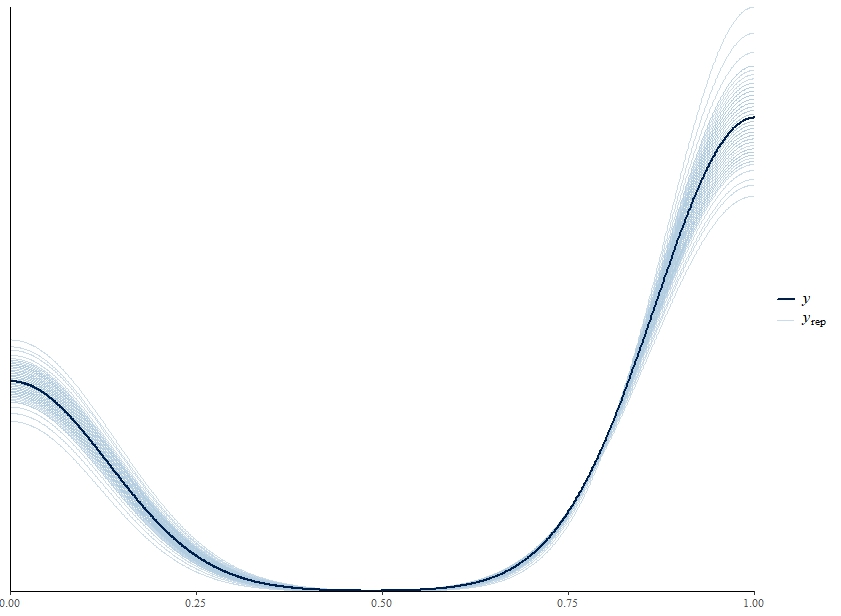


**GIRAFFE**

Table S5. Posterior summaries from the posthoc Bayesian regression model with weak priors evaluating covariates influencing giraffe (*giraffa giraffa*) detection probability at camera clusters. Continuous variables log-transformed using log1p to reduce skew. Spatial and temporal predictors were standardized (z-score scaled). Columns include posterior mean estimates, standard errors and 95% credible intervals. Parameters that do not overlap zero are interpreted as having strong evidence of effect.

| **Parameter** | **Estimate** | **Est. Error** | **CrI (2.5%)** | **CrI (97.5%)** |
| --- | --- | --- | --- | --- |
| Intercept | -1.979 | 4.957 | -11.587 | 7.802 |
| Visibility | -0.721 | 1.253 | -3.163 | 1.732 |
| Site Cover | -0.075 | 1.154 | -2.346 | 2.187 |
| Human use | 0.379 | 0.756 | -1.115 | 1.856 |
| Luminosity | -0.121 | 0.774 | -1.626 | 1.387 |
| Dry Season | 1.343 | 1.787 | -2.164 | 4.797 |
| Eland detects | 0.958 | 0.292 | 0.407 | 1.551 |
| Kudu detects | 0.368 | 0.210 | -0.042 | 0.778 |
| Lion present | 0.939 | 1.065 | -1.016 | 3.154 |
| Lion detects | 0.507 | 1.322 | -2.088 | 3.093 |
| Spotted hyena detects | 1.606 | 1.187 | -0.730 | 3.925 |
| Lion long term | 0.970 | 1.269 | -1.483 | 3.481 |
| Visibility x Lion detects | 0.274 | 0.946 | -1.570 | 2.126 |
| Site Cover x Spotted hyena detects | -1.888 | 1.158 | -4.170 | 0.365 |
| Luminosity x Lion detects | -0.808 | 0.769 | -2.328 | 0.675 |
| Dry Season x Lion present | 1.718 | 1.256 | -0.421 | 4.423 |
| Dry Season x Lion long term | 0.969 | 0.599 | -0.182 | 2.152 |
| Lion detects x NDVI | -0.964 | 0.401 | -1.787 | -0.211 |
| Spotted hyena detects x NDVI | -1.229 | 0.513 | -2.272 | -0.249 |
| Lion long term x NDVI | 0.638 | 0.279 | 0.127 | 1.218 |
|  |  |  |  |  |
| **Random Effects** (Intercept) | | |  |  |
| Camera Cluster | 0.37 | 0.26 | 0.01 | 0.96 |
| Survey | 5.13 | 1.45 | 2.76 | 8.4 |

**GIRAFFE BAYESIAN POSTHOC PLOTS**

Figure S15. Marginal effects plots from the Bayesian posthoc model showing predicted detection probability of southern giraffe (*Giraffa giraffa*) at camera clusters across continuous covariates in northwest Namibia. Species and environmental predictors include dry season progression, eland (*Taurotragus oryx*) detections, human usage, kudu (*Tragelaphus strepsiceros*), lion (*Panthera leo*) detection rate, lion long-term activity, luminosity, camera cluster vegetation cover, spotted hyena (*Crocuta crocuta*) detections, and camera cluster visibility. Shaded ribbons represent 95% credible intervals.


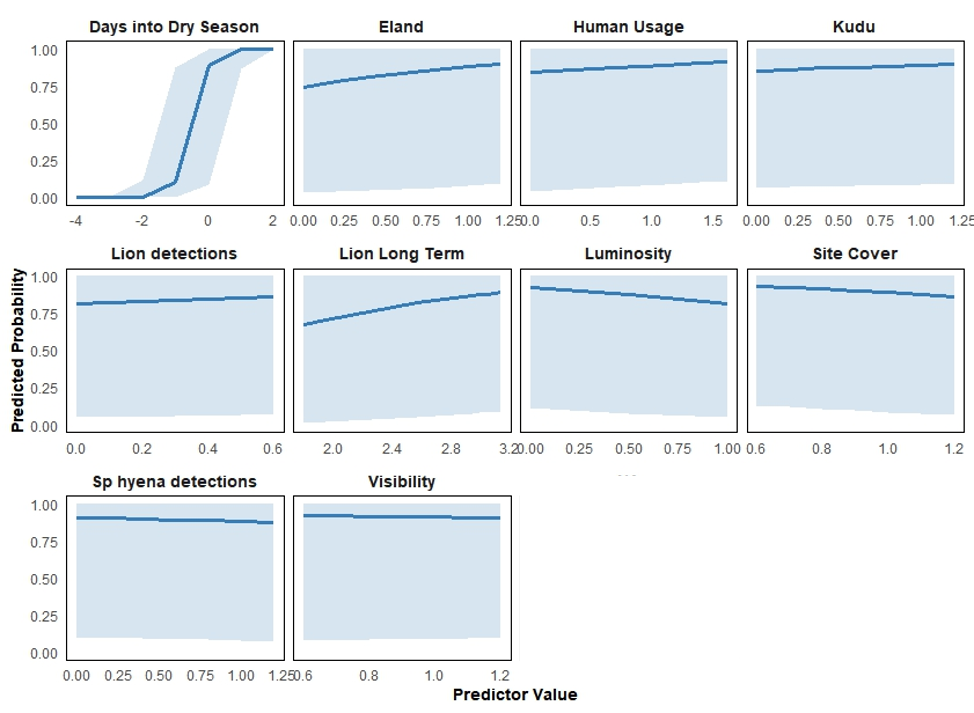


Figure S16**.** Marginal effect of lion (*Panthera leo*) daily presence on southern giraffe (*Giraffa giraffa*) detection probability. Posterior means and 95% credible intervals estimated from Bayesian posthoc model based on camera trap data in northwest Namibia.


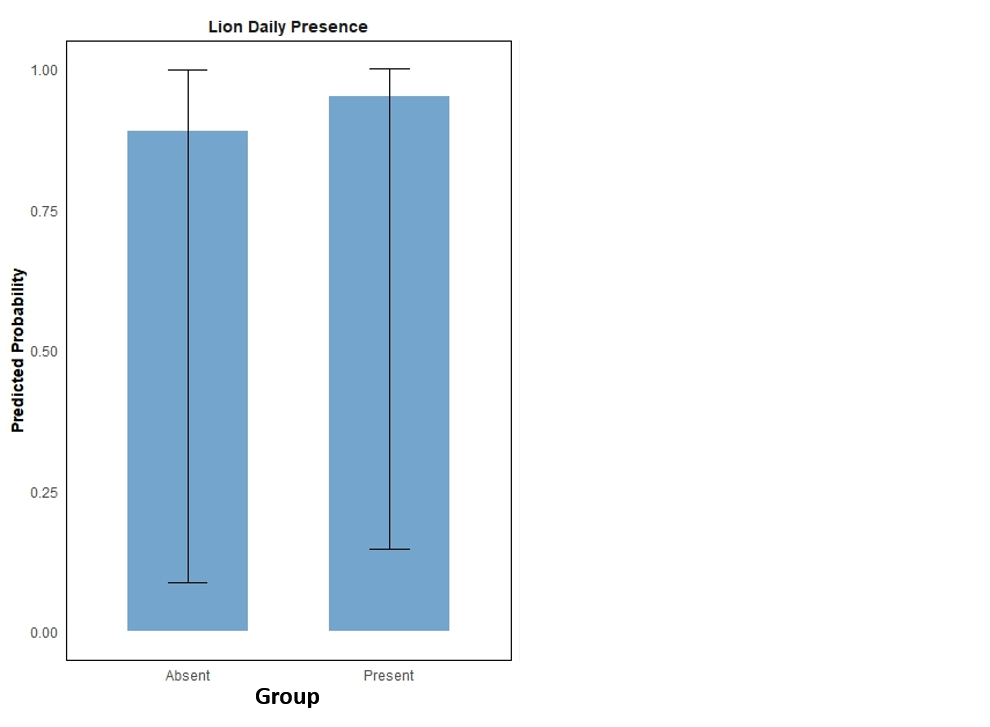


Figure S17. Predicted southern giraffe (*Giraffa giraffa*) detection probabilities based on significant two-way interaction effects. Panels show interactions between (top left) long-term lion (*Panthera leo*) activity and dry season progression, (top right) luminosity and lion detection rate, (bottom left) camera cluster vegetation cover and spotted hyena (*Crocuta crocuta*) detections, (bottom right) interaction between camera cluster visibility and lion detection rate. Estimates derived from Bayesian posthoc model applied to camera trap data in northwest Namibia.


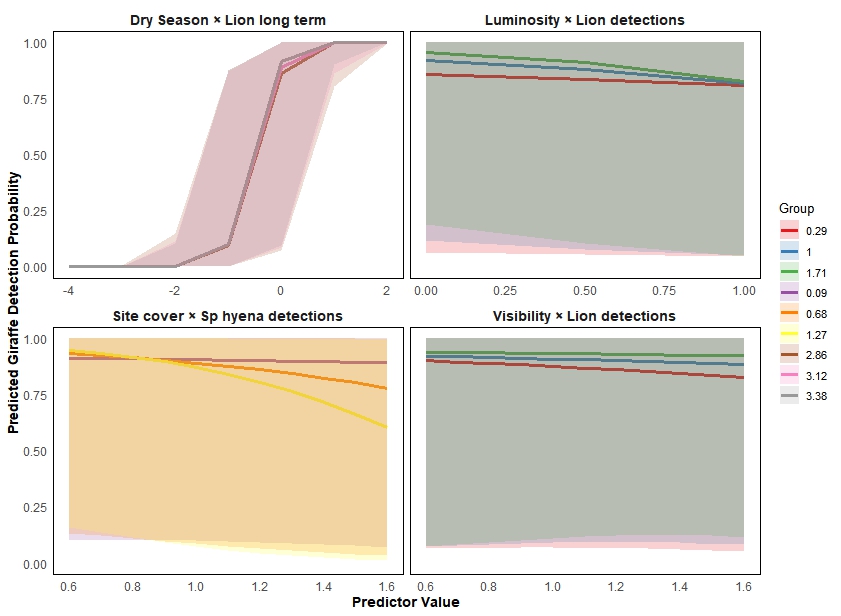


Figure S18. Predicted southern giraffe (*Giraffa giraffa*) detection probabilities in relation to vegetation productivity (NDVI - standardized) and predator presence. Left: interaction between NDVI and lion (*Panthera leo*) detection rate. Center: interaction between NDVI and long-term lion activity. Right: interaction between NDVI and spotted hyena (*Crocuta crocuta*) detections. Data come from Bayesian posthoc models of camera trap surveys in northwest Namibia.


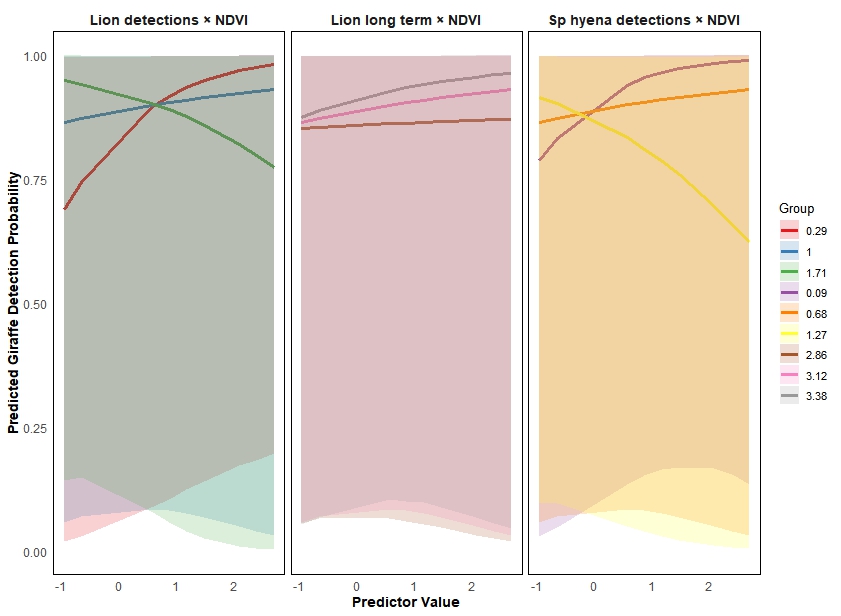


Figure S19. Predicted southern giraffe (*Giraffa giraffa*) detection probability across a binned index of dry season progression, grouped by lion (*Panthera leo*) presence (blue) or absence (red) within the previous 24 hours. Bars represent posterior means and 95% credible intervals from Bayesian posthoc camera trap models in northwest Namibia.


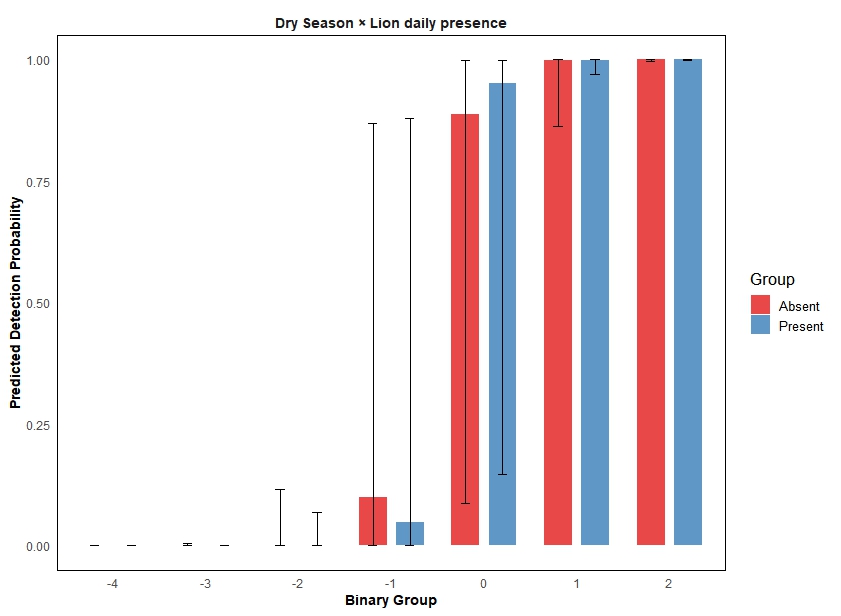


Figure S20. Comparison of top-model predictions versus model-averaged predictions for southern giraffe (*Giraffa giraffa*) detection probability. Points represent posterior means of predicted probabilities from the top-ranked and model-averaged Bayesian posthoc models. Based on camera trap data from northwest Namibia.


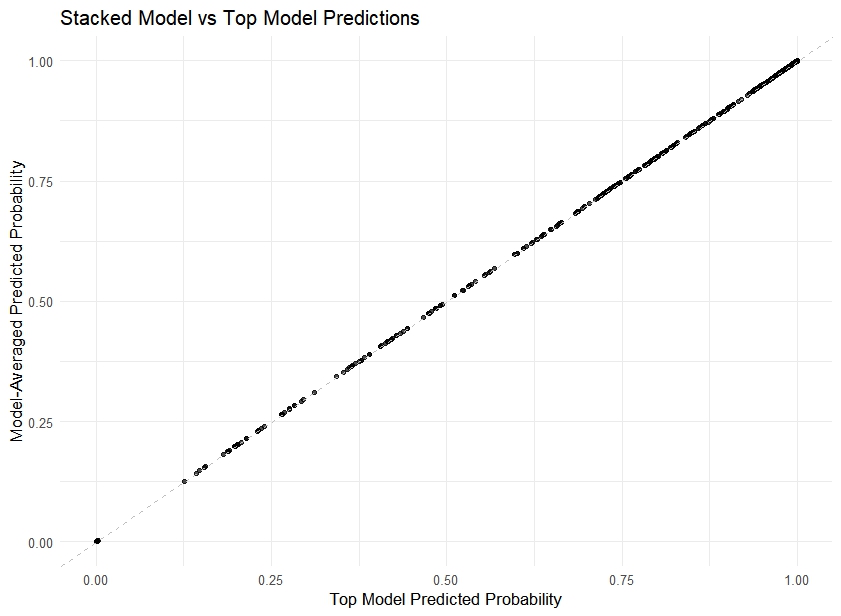


Figure S21. Posterior predictive checks for southern giraffe (*Giraffa giraffa*) Bayesian posthoc model. The observed detection response (dark line) is plotted against simulated responses (light lines) from the posterior distribution, indicating strong model fit to the empirical data.


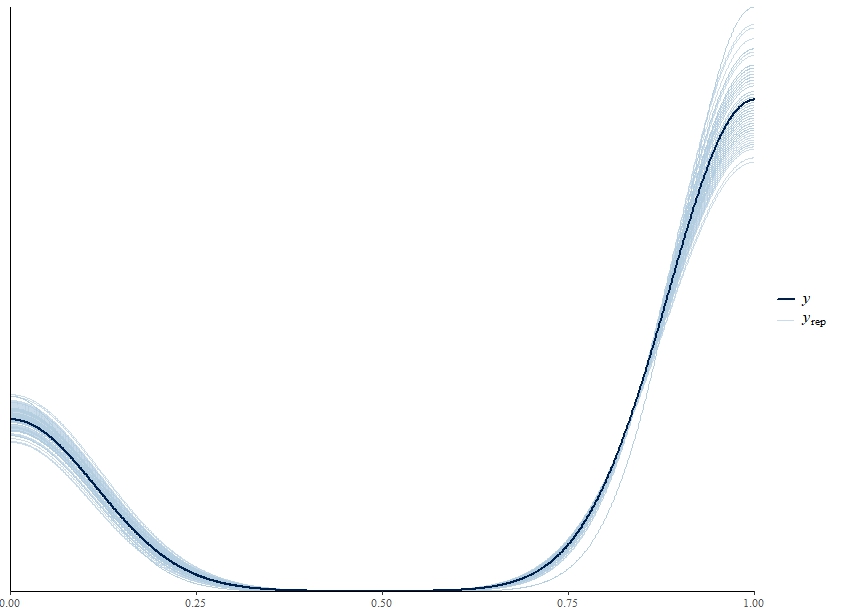


**KUDU**

Table S6. Posterior summaries from the posthoc Bayesian regression model with weak priors evaluating covariates influencing kudu (*Tragelaphus strepsiceros*) detection probability at camera clusters. Continuous variables log-transformed using log1p to reduce skew. Spatial and temporal predictors were standardized (z-score scaled). Columns include posterior mean estimates, standard errors and 95% credible intervals. Parameters that do not overlap zero are interpreted as having strong evidence of effect.

| **Parameter** | **Estimate** | **Est. Error** | **CrI (2.5%)** | **CrI (97.5%)** |
| --- | --- | --- | --- | --- |
| Intercept | -7.501 | 15.151 | -36.707 | 22.249 |
| Site cover | 0.732 | 1.877 | -2.934 | 4.411 |
| Temperature | 1.848 | 0.898 | 0.090 | 3.627 |
| Concession/Cons | 2.055 | 0.942 | 0.282 | 3.985 |
| Longitude | 1.905 | 1.210 | -0.487 | 4.251 |
| Elevation | -0.143 | 1.988 | -4.073 | 3.745 |
| Dry season | 1.165 | 1.814 | -2.372 | 4.705 |
| Sp hyena present | -0.360 | 1.416 | -3.074 | 2.476 |
| Sp hyena detects | -0.998 | 0.655 | -2.343 | 0.238 |
| Lion detects | 0.561 | 0.393 | -0.206 | 1.349 |
| Lion long term | -0.936 | 1.800 | -4.442 | 2.597 |
| Eland detects | 1.308 | 0.520 | 0.328 | 2.384 |
| Site cover x Lion long term | 1.075 | 0.798 | -0.473 | 2.646 |
| Dry season x Sp hyena present | -1.933 | 1.568 | -5.083 | 1.054 |
| Dry season x Lion long term | 1.837 | 0.691 | 0.506 | 3.208 |
|  |  |  |  |  |
| **Random Effects** (Intercept) | | |  |  |
| Camera Cluster | 1.23 | 0.4 | 0.51 | 2.08 |
| Survey | 4.12 | 1.3 | 2.1 | 7.23 |

**KUDU BAYESIAN POSTHOC PLOTS**

Figure S22. Marginal effects of continuous predictors on kudu (*Tragelaphus strepsiceros*) detection probability. Predictors include dry season progression, eland (*Taurotragus oryx*) detections, elevation, lion (*Panthera leo*) detections, long-term lion activity, longitude, camera cluster vegetation cover, spotted hyena (*Crocuta crocuta*) detections, and temperature (modelled). Lines represent posterior mean estimates; shaded areas represent 95% credible intervals. Derived from Bayesian posthoc models from camera trap surveys in northwest Namibia.


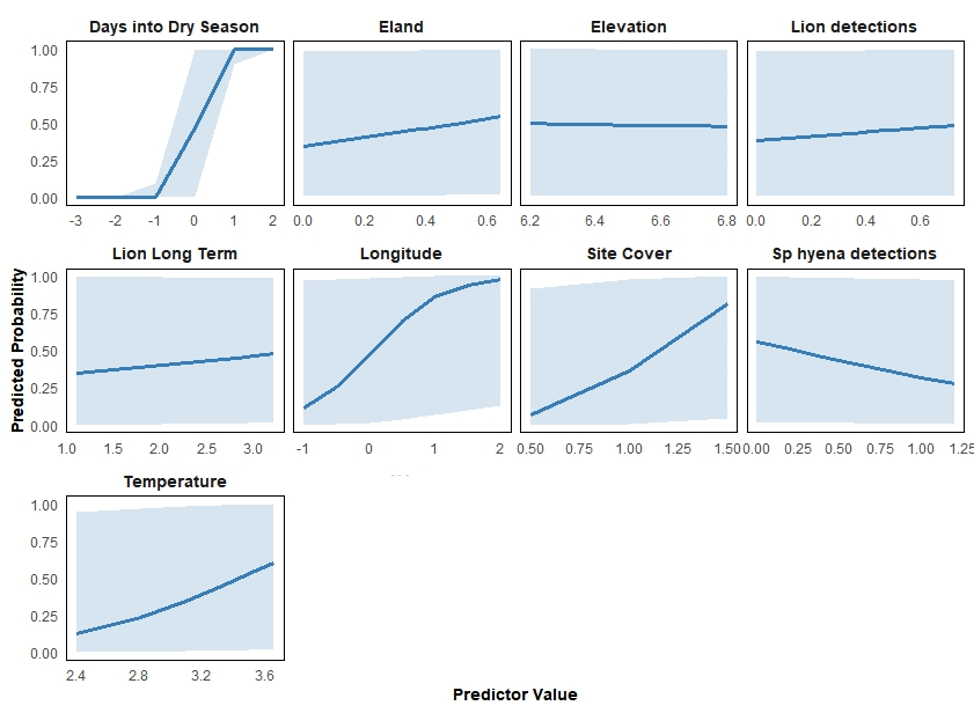


Figure S23. Marginal predicted detection probabilities of kudu (*Tragelaphus strepsiceros*) across binary predictors including land tenure (government vs. conservancy) and daily presence of spotted hyenas (*Crocuta crocuta*). Estimates and 95% credible intervals are based on Bayesian posthoc models from camera trap data in northwest Namibia.


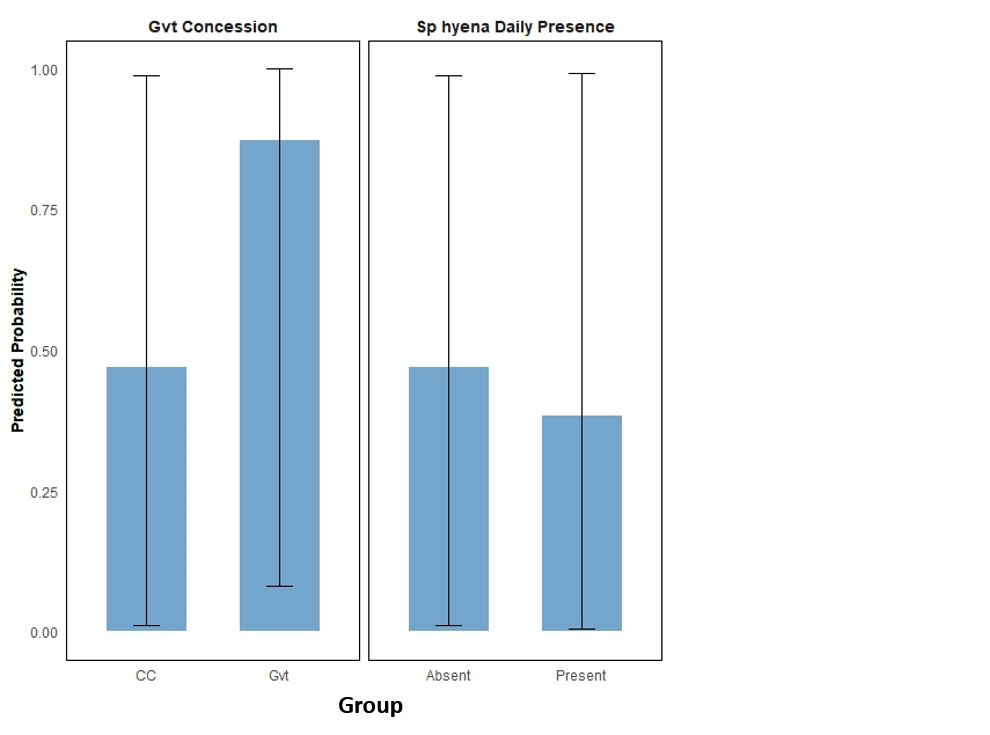


Figure S24. Interaction plots illustrating the predicted detection probability of kudu (*Tragelaphus strepsiceros*) as a function of (left) dry season × long-term lion (*Panthera leo*) activity, (center) dry season × spotted hyena (*Crocuta crocuta*) daily presence, and camera cluster vegetation cover × long-term lion activity. Shaded areas represent 95% credible intervals from Bayesian posthoc models based on camera trap data in northwest Namibia.


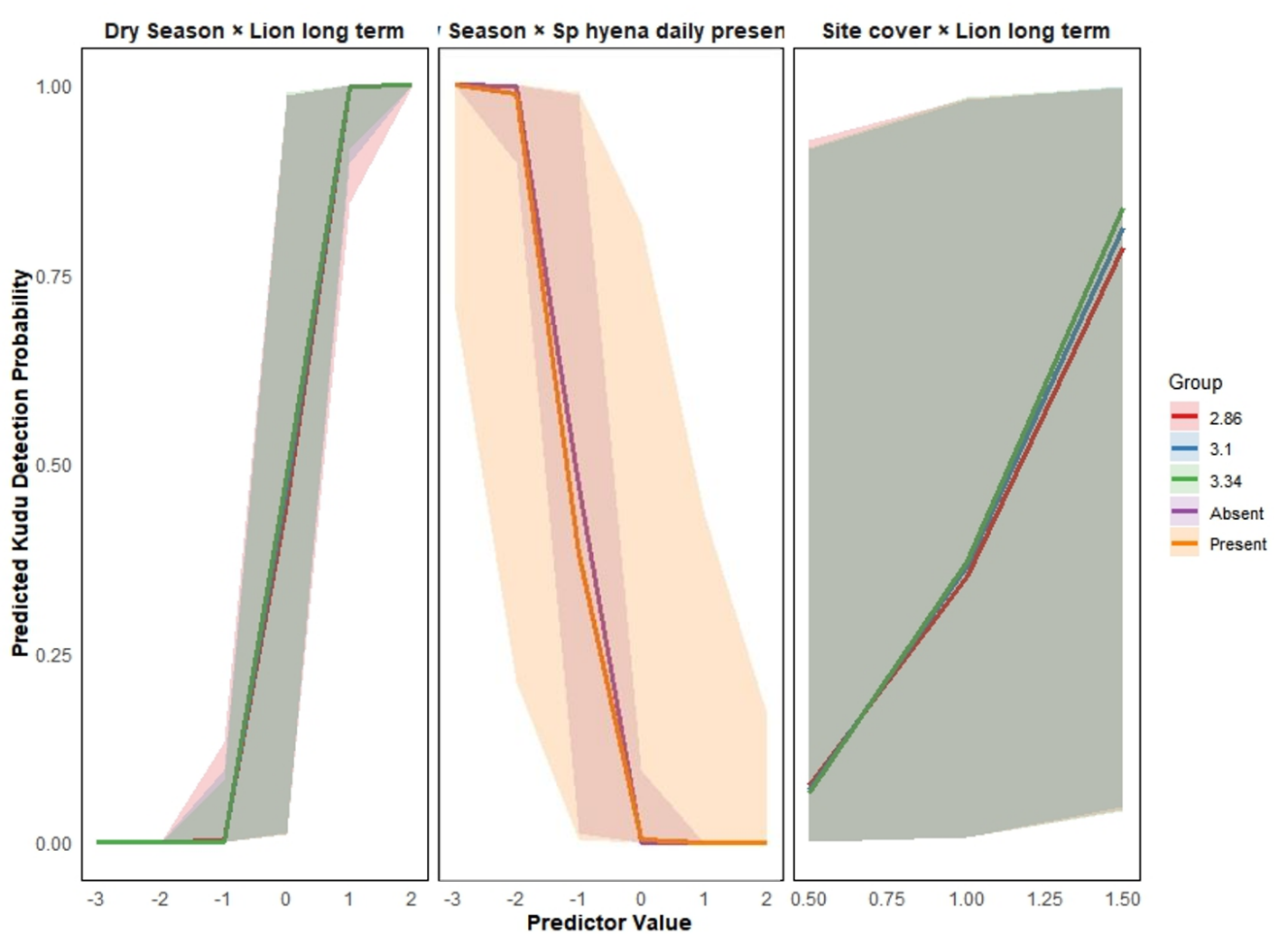


Figure S25. Predicted probabilities of kudu (*Tragelaphus strepsiceros*) detection comparing model-averaged estimates and top model predictions from Bayesian posthoc analysis of camera trap data in northwest Namibia.


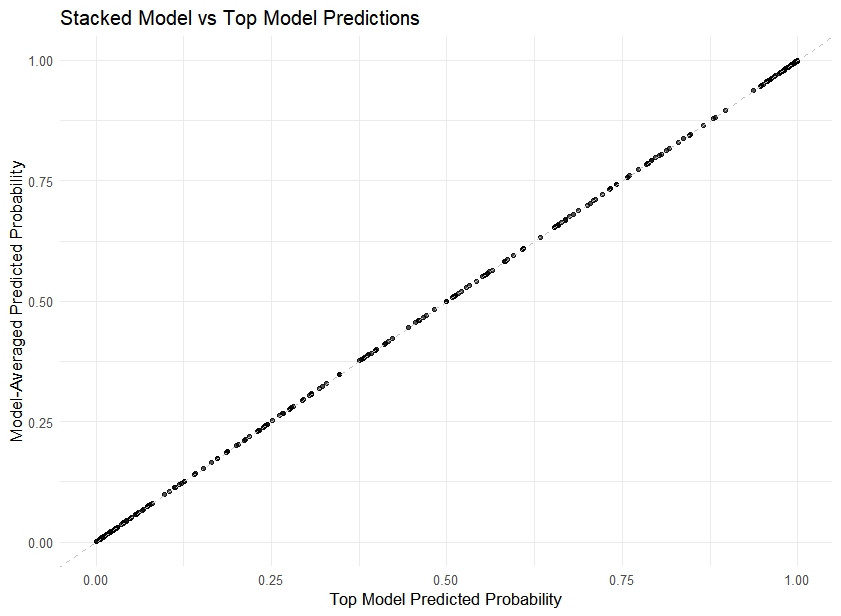


Figure S26. Posterior predictive check plot comparing simulated data to observed detection probabilities for kudu (*Tragelaphus strepsiceros*). Model fit was assessed using Bayesian posthoc model diagnostics from camera trap surveys in northwest Namibia.


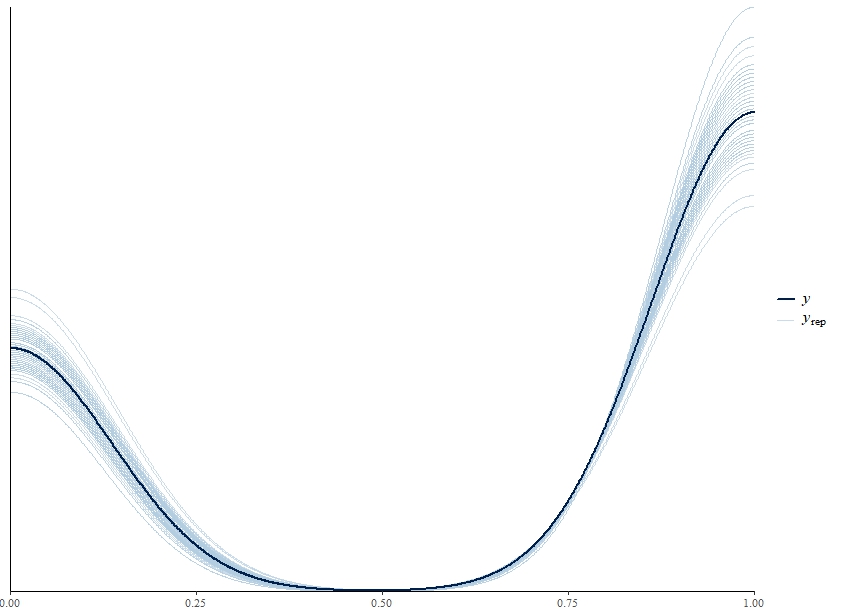


**MOUNTAIN ZEBRA**

Table S7. Posterior summaries from the posthoc Bayesian regression model with weak priors evaluating covariates influencing mountain zebra (*Equus zebra*) detection probability at camera clusters. Continuous variables log-transformed using log1p to reduce skew. Spatial and temporal predictors were standardized (z-score scaled). Columns include posterior mean estimates, standard errors and 95% credible intervals. Parameters that do not overlap zero are interpreted as having strong evidence of effect.

| **Parameter** | **Estimate** | **Est. Error** | **CrI (2.5%)** | **CrI (97.5%)** |
| --- | --- | --- | --- | --- |
| Intercept | 8.314 | 4.611 | -0.645 | 17.508 |
| Site Cover | 0.397 | 1.900 | -3.345 | 4.101 |
| Dist to water | -1.445 | 0.621 | -2.697 | -0.217 |
| Luminosity | 0.722 | 0.539 | -0.334 | 1.772 |
| Elevation | 1.283 | 0.429 | 0.493 | 2.185 |
| Dry season | 3.117 | 1.673 | -0.135 | 6.388 |
| Concession/Cons | 2.668 | 1.013 | 0.689 | 4.677 |
| Springbok detects | 1.171 | 0.239 | 0.719 | 1.660 |
| Sp hyena detects | 1.046 | 1.201 | -1.295 | 3.391 |
| Lion detects | 0.175 | 1.194 | -2.175 | 2.518 |
| Lion long term | -1.560 | 1.165 | -3.900 | 0.673 |
| Site Cover x Sp hyena detects | -0.747 | 1.258 | -3.214 | 1.708 |
| Site Cover x Lion detects | -1.002 | 1.153 | -3.293 | 1.254 |
| Site Cover x Lion long term | -0.401 | 0.770 | -1.932 | 1.101 |
| Dry season x Lion detects | -0.723 | 0.326 | -1.401 | -0.118 |
| Dry season x Lion long term | 2.421 | 0.649 | 1.186 | 3.719 |
|  |  |  |  |  |
| **Random Effects** (Intercept) | | |  |  |
| Camera Cluster | 1.43 | 0.3 | 0.91 | 2.07 |
| Survey | 8.96 | 1.81 | 5.95 | 13.07 |

**MOUNTAIN ZEBRA BAYESIAN POSTHOC PLOTS**

Figure S27. Marginal effects plots from Bayesian posthoc model showing predicted detection probability of mountain zebra (*Equus zebra*) across continuous covariates in northwest Namibia. Species and environmental predictors include dry season progression, distance to water, elevation, lion (*Panthera leo*) detections, lion long-term activity, luminosity, camera cluster vegetation cover, spotted hyena (*Crocuta crocuta*) detections, and springbok (*Antidorcas marsupialis*) detections. Shaded ribbons represent 95% credible intervals.


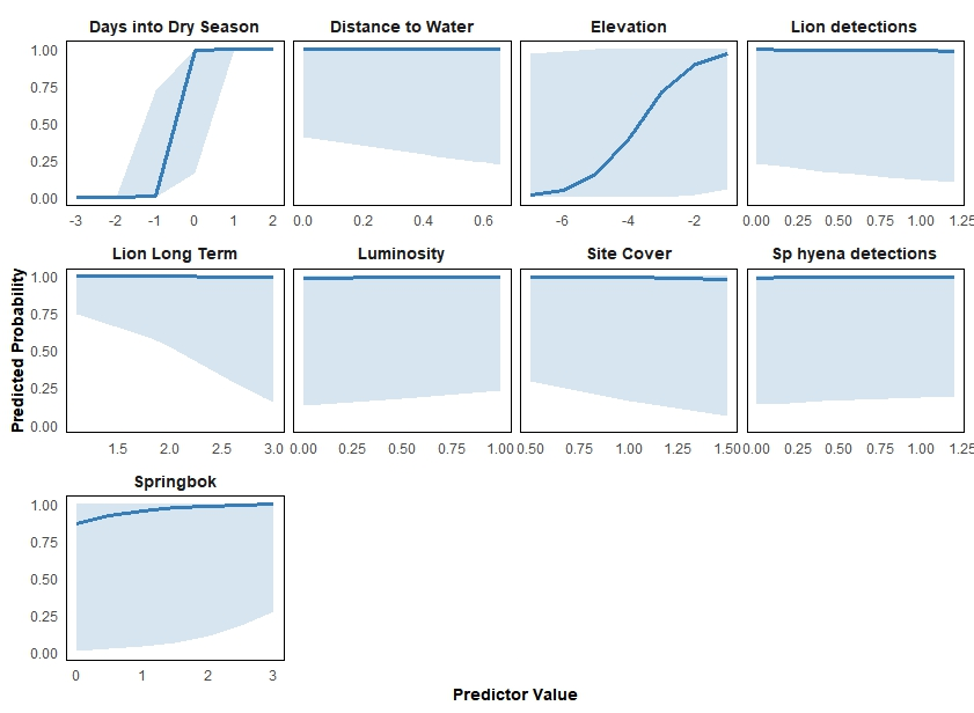


Figure S28. Marginal predicted detection probabilities of mountain zebra (*Equus zebra*) across land tenure binary predictor (government vs. conservancy). Estimates and 95% credible intervals are based on Bayesian posthoc models from camera trap data in northwest Namibia.


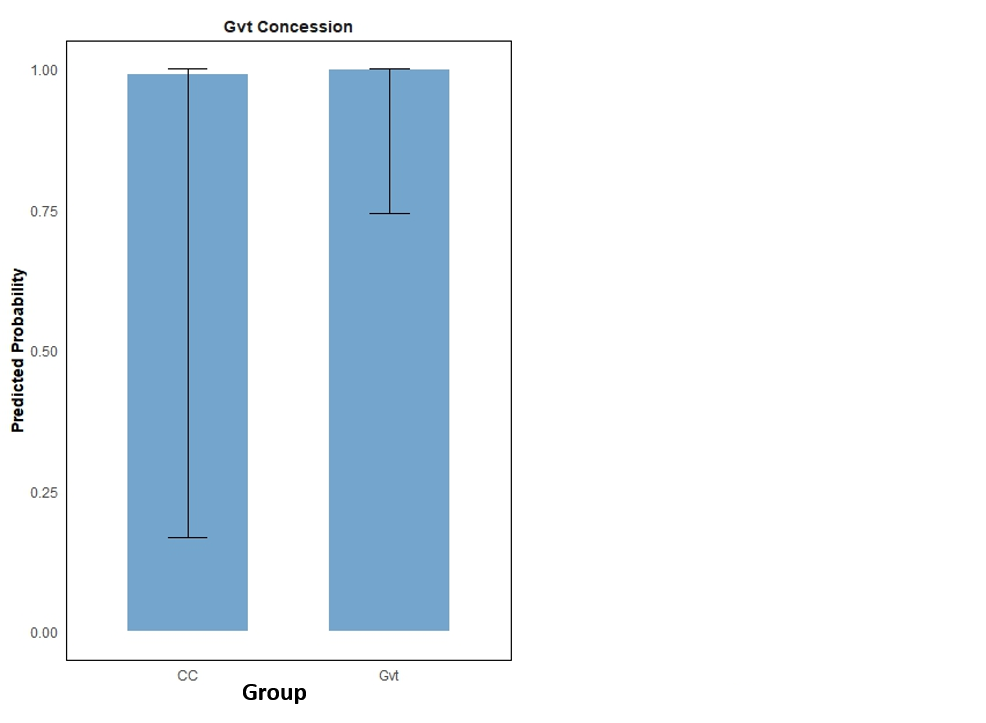


Figure S29. Interaction effects plots showing predicted detection probability of mountain zebra (*Equus zebra*) in response to interactions between (top left) dry season progression and lion (*Panthera leo*) detections, (top right) camera cluster vegetation cover and lion detections, (bottom left) camera cluster vegetation cover and lion long-term activity, and (bottom right) camera cluster vegetation cover and spotted hyena (*Crocuta crocuta*) detections. Shaded areas represent 95% credible intervals around predicted detection probability across a gradient of environmental or predator presence values.


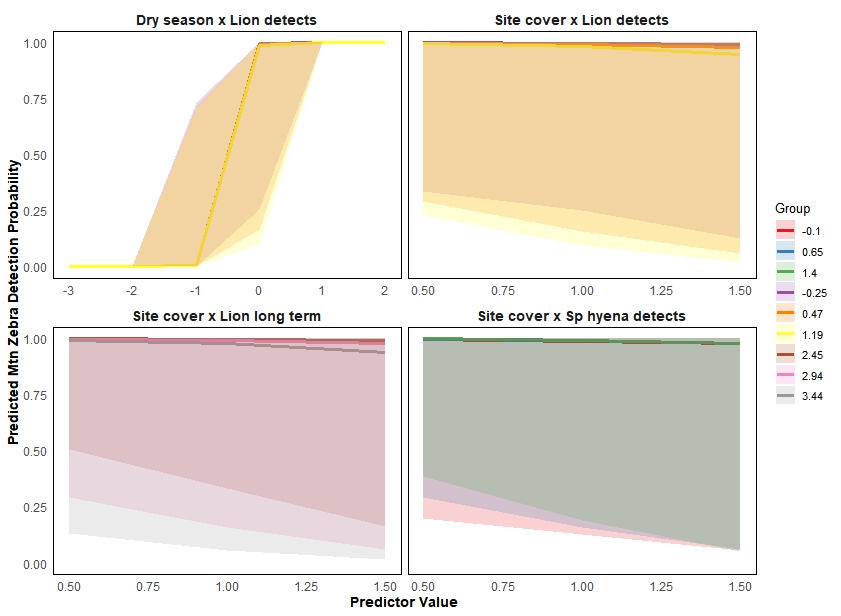


Figure S30. Predicted detection probability of mountain zebra (*Equus zebra*) as a function of long-term lion (*Panthera leo*) activity across the dry season. Results from the Bayesian posthoc model; lines show mean predicted values from the Bayesian posthoc model, with shaded ribbons showing 95% credible intervals, based on camera trap data from northwest Namibia.


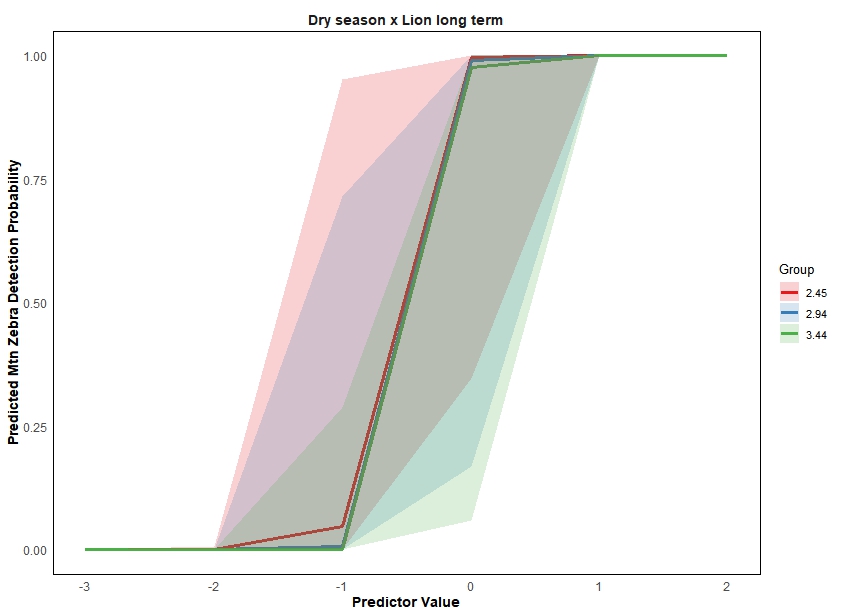


Figure S31. Model-averaged predicted detection probability vs. top model predictions for mountain zebra (*Equus zebra*) from the Bayesian posthoc model. Strong alignment along the 1:1 line indicates close agreement between the best-fit model and the stacked model ensemble.


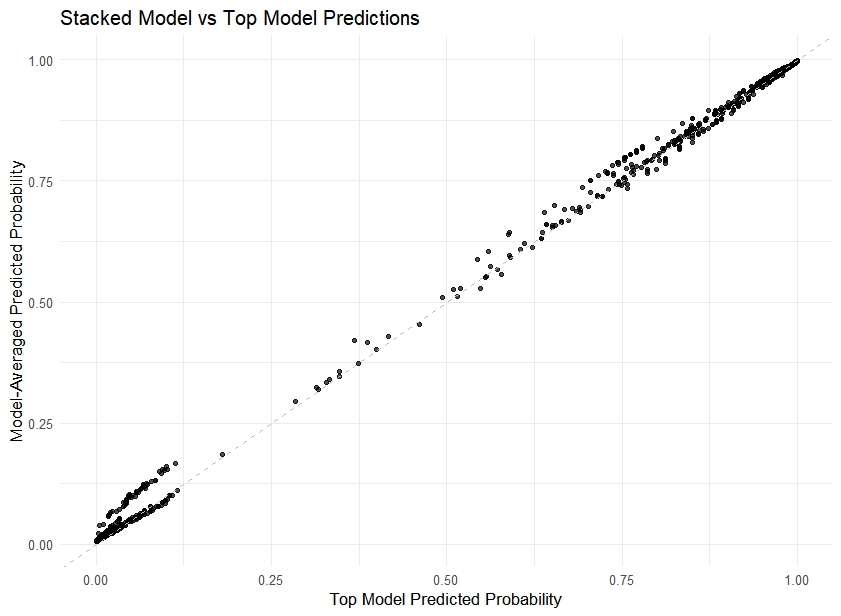


Figure S32. Posterior predictive check for the Bayesian posthoc model predicting mountain zebra (*Equus zebra*) detection probability. The observed values (dark line) fall within the predictive distribution of replicated data (light lines), indicating adequate model fit.


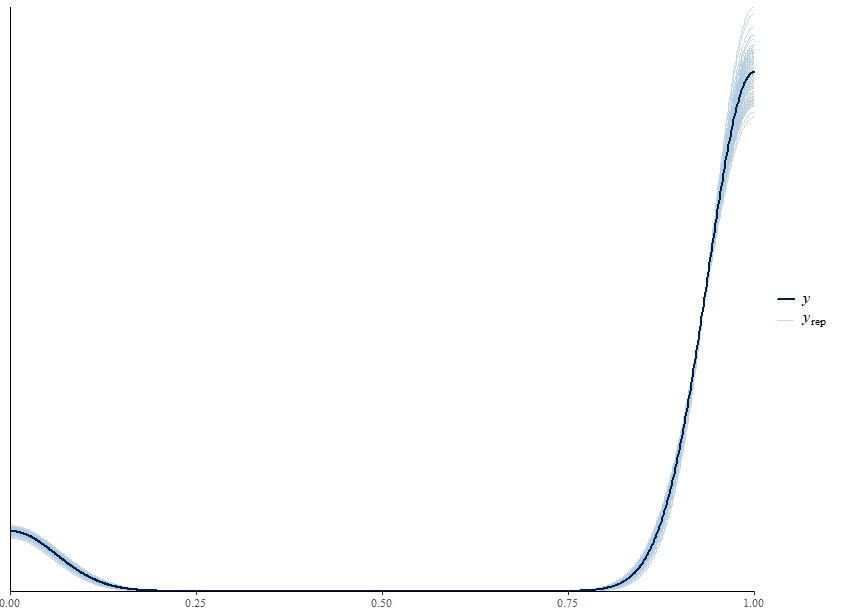


**SPRINGBOK**

Table S8. Posterior summaries from the posthoc Bayesian regression model with weak priors evaluating covariates influencing springbok (*Antidorcas marsupialis*) detection probability at camera clusters. Continuous variables log-transformed using log1p to reduce skew. Spatial and temporal predictors were standardized (z-score scaled). Columns include posterior mean estimates, standard errors and 95% credible intervals. Parameters that do not overlap zero are interpreted as having strong evidence of effect.

| **Parameter** | **Estimate** | **Est. Error** | **CrI (2.5%)** | **CrI (97.5%)** |
| --- | --- | --- | --- | --- |
| Intercept | 1.526 | 3.998 | -6.300 | 9.336 |
| Visibility | 2.563 | 1.319 | -0.046 | 5.116 |
| Site cover | -0.357 | 1.228 | -2.742 | 2.075 |
| Luminosity | 0.897 | 0.514 | -0.109 | 1.916 |
| Dry season | 1.892 | 1.459 | -0.951 | 4.758 |
| Sp hyena present | 0.219 | 1.405 | -2.496 | 2.974 |
| Lion detects | 0.333 | 0.311 | -0.282 | 0.948 |
| Lion long term | -0.937 | 0.773 | -2.472 | 0.579 |
| Mtn zebra detects | 0.769 | 0.145 | 0.497 | 1.069 |
| Site cover x Sp hyena present | 1.188 | 1.441 | -1.601 | 4.020 |
| Dry season x Lion long term | 0.756 | 0.508 | -0.207 | 1.779 |
|  |  |  |  |  |
| **Random Effects** (Intercept) | | |  |  |
| Camera Cluster | 0.74 | 0.29 | 0.14 | 1.34 |
| Survey | 5.69 | 1.51 | 3.2 | 9.07 |

**SPRINGBOK BAYESIAN POSTHOC PLOTS**

Figure S33. Marginal effects plots from the Bayesian posthoc model showing predicted detection probability of springbok (*Antidorcas marsupialis* across continuous covariates from trail camera data in northwest Namibia. Species and environmental predictors include dry season progression, lion (*Panthera leo*) detections, lion long-term activity, luminosity, mountain zebra (*Equus zebra*) detections, camera cluster vegetation cover, and visibility. Shaded ribbons represent 95% credible intervals.


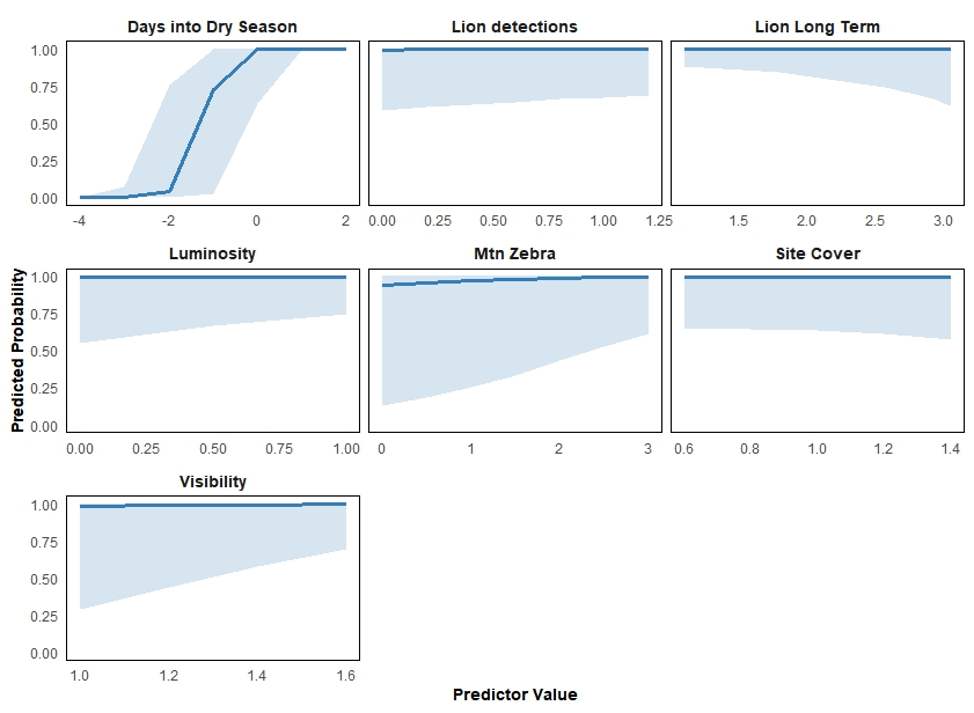


Figure S34. Continuous binary effects from the Bayesian posthoc model for springbok (*Antidorcas marsupialis*), depicting detection probability responses to spotted hyena (*Crocuta crocuta*) daily presence covariate, derived from camera traps in northwest Namibia.


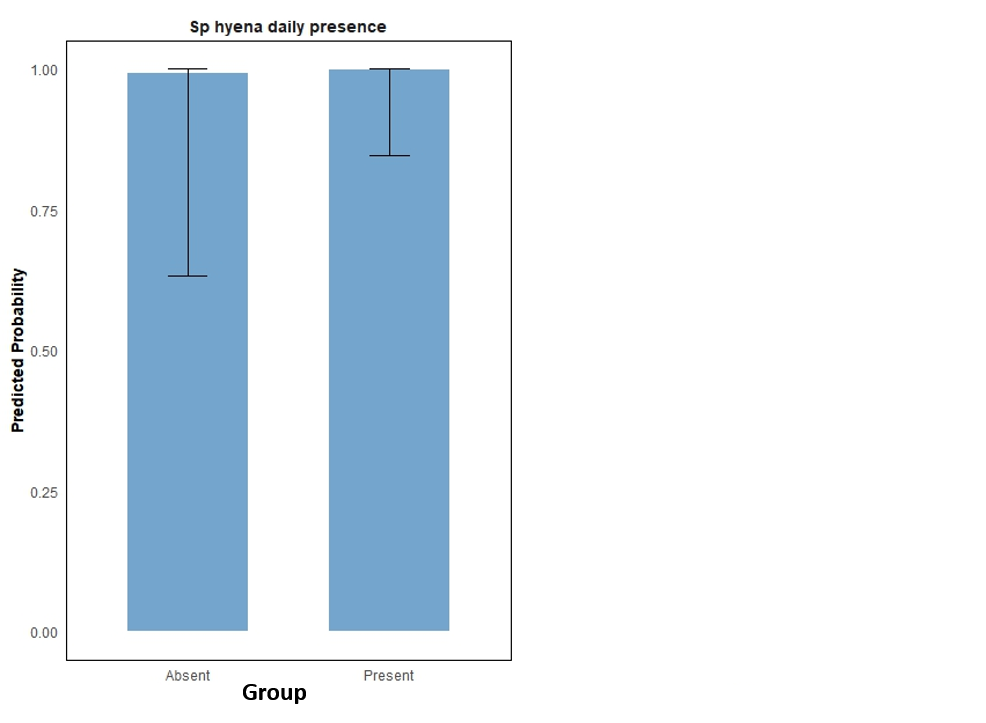


Figure S35. Predicted springbok (*Antidorcas marsupialis*) detection probability for the interaction between dry season × long-term lion (*Panthera leo*) activity. Lines show mean predicted values from the Bayesian posthoc model, with shaded ribbons showing 95% credible intervals, based on camera trap data from northwest Namibia.


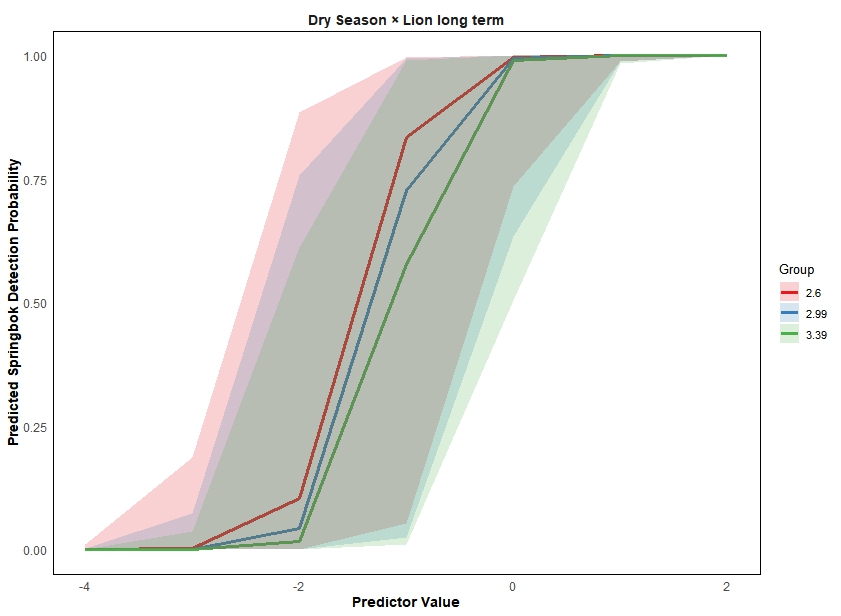


Figure S36. Predicted springbok (*Antidorcas marsupialis*) detection probability for the interaction between camera cluster vegetation cover × spotted hyena (*Crocuta crocuta*) daily presence. Lines show mean predicted values from the Bayesian posthoc model, with shaded ribbons showing 95% credible intervals, based on camera trap data from northwest Namibia.


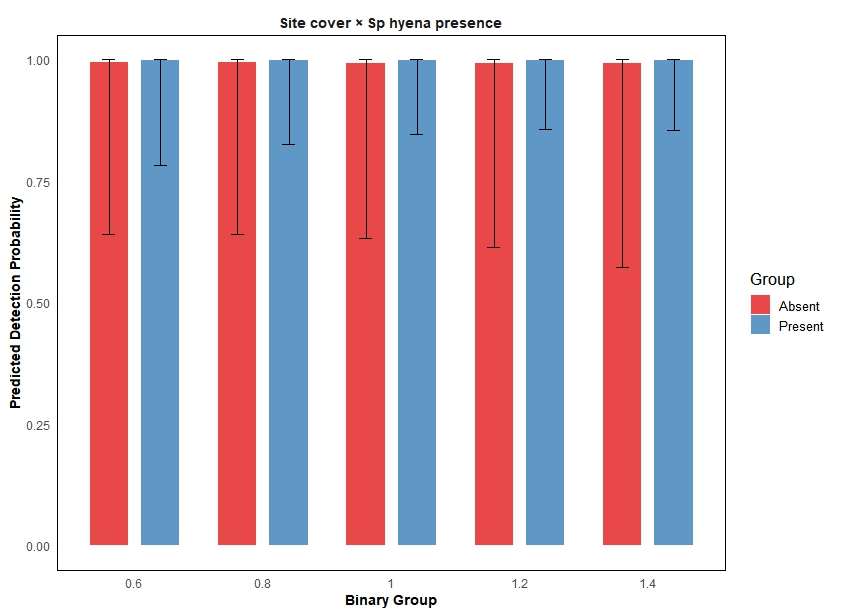


Figure S37. Comparison between model-averaged predicted detection probabilities and those from the top-performing Bayesian posthoc model for springbok (*Antidorcas marsupialis*), showing close agreement and high predictive accuracy.


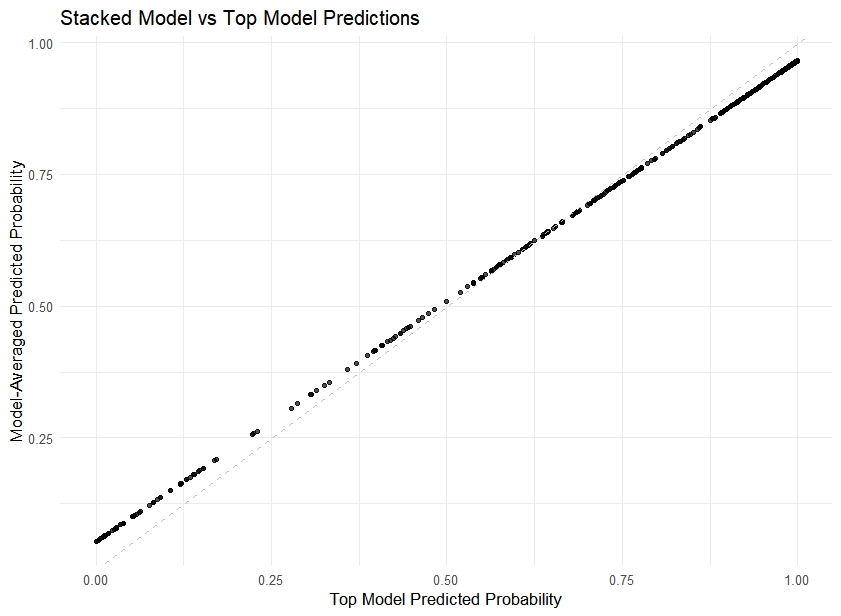


Figure S38. Posterior predictive checks for springbok (*Antidorcas marsupialis*) Bayesian posthoc model. Light blue lines show simulated posterior draws, with observed response curve in black, indicating good model fit.


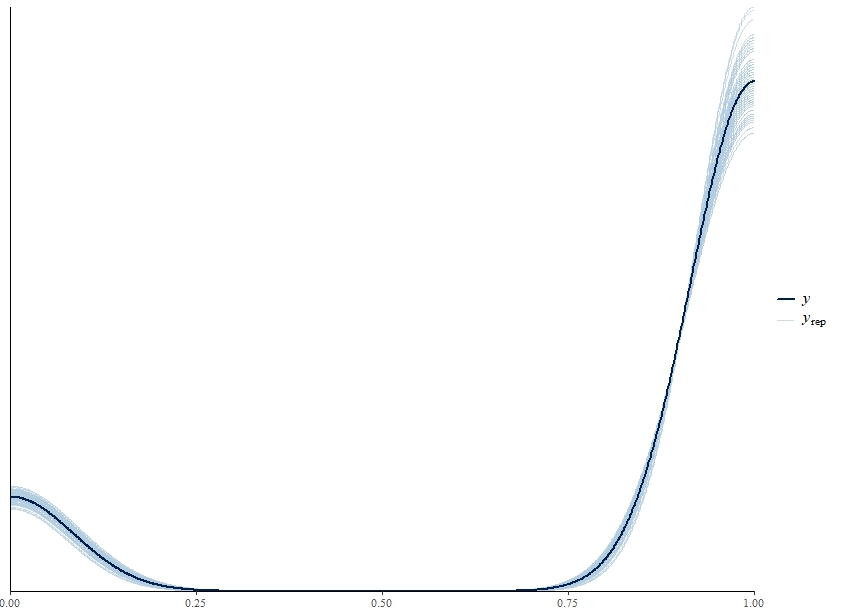


**BLACK RHINO**

Table S9. Posterior summaries from the posthoc Bayesian regression model with weak priors evaluating covariates influencing Black rhinoceros (*Diceros bicornis*) detection probability at camera clusters. Continuous variables log-transformed using log1p to reduce skew. Spatial and temporal predictors were standardized (z-score scaled). Columns include posterior mean estimates, standard errors and 95% credible intervals. Parameters that do not overlap zero are interpreted as having strong evidence of effect.

| **Parameter** | **Estimate** | **Est. Error** | **CrI (2.5%)** | **CrI (97.5%)** |
| --- | --- | --- | --- | --- |
| Intercept | 2.320 | 4.446 | -6.191 | 11.525 |
| Visibility | -0.337 | 1.715 | -3.707 | 3.004 |
| Site cover | 0.564 | 1.541 | -2.465 | 3.580 |
| Dist to water | -0.410 | 0.965 | -2.247 | 1.540 |
| Human use | -0.772 | 1.188 | -3.123 | 1.545 |
| Latitude | -0.087 | 1.479 | -2.998 | 2.845 |
| Longitude | 1.410 | 1.348 | -1.216 | 4.040 |
| Dry season | 1.569 | 1.724 | -1.805 | 4.968 |
| Concession/Cons | -0.057 | 1.764 | -3.432 | 3.483 |
| Br hyena detects | 1.177 | 0.762 | -0.310 | 2.694 |
| Giraffe detects | -0.611 | 0.599 | -1.818 | 0.532 |
| Lion detects | 0.496 | 1.641 | -2.703 | 3.716 |
| Sp hyena detects | 0.139 | 0.385 | -0.630 | 0.885 |
| Lion long term | 0.372 | 1.482 | -2.564 | 3.245 |
| Visibility x Lion detects | 1.432 | 1.095 | -0.725 | 3.596 |
| Visibility x Lion long term | -1.292 | 0.885 | -3.043 | 0.430 |
| Site cover x Lion detects | -0.381 | 1.166 | -2.647 | 1.895 |
| Dry season x Sp hyena present | -2.110 | 1.325 | -4.955 | 0.261 |
| Dry season x Lion long term | 0.564 | 0.758 | -0.835 | 2.122 |
|  |  |  |  |  |
| **Random Effects** (Intercept) | | |  |  |
| Camera Cluster | 0.47 | 0.41 | 0.01 | 1.5 |
| Survey | 2.23 | 1.5 | 0.1 | 5.68 |

**BLACK RHINO BAYESIAN POSTHOC PLOTS**

Figure S39. Marginal effects plots from the Bayesian posthoc model showing predicted detection probability of black rhinoceros (*Diceros bicornis*) across continuous covariates in northwest Namibia. Species and environmental predictors include brown hyena (*Parahyeana brunnea*), dry season progression, distance to water, southern giraffe (*Giraffa giraffa*) detections, human area usage, latitude, lion (*Panethera leo*) detections, lion long-term activity, longitude, camera cluster vegetation cover, spotted hyena (*Crocuta crocuta*) detections, and camera cluster visibility. Shaded ribbons represent 95% credible intervals.


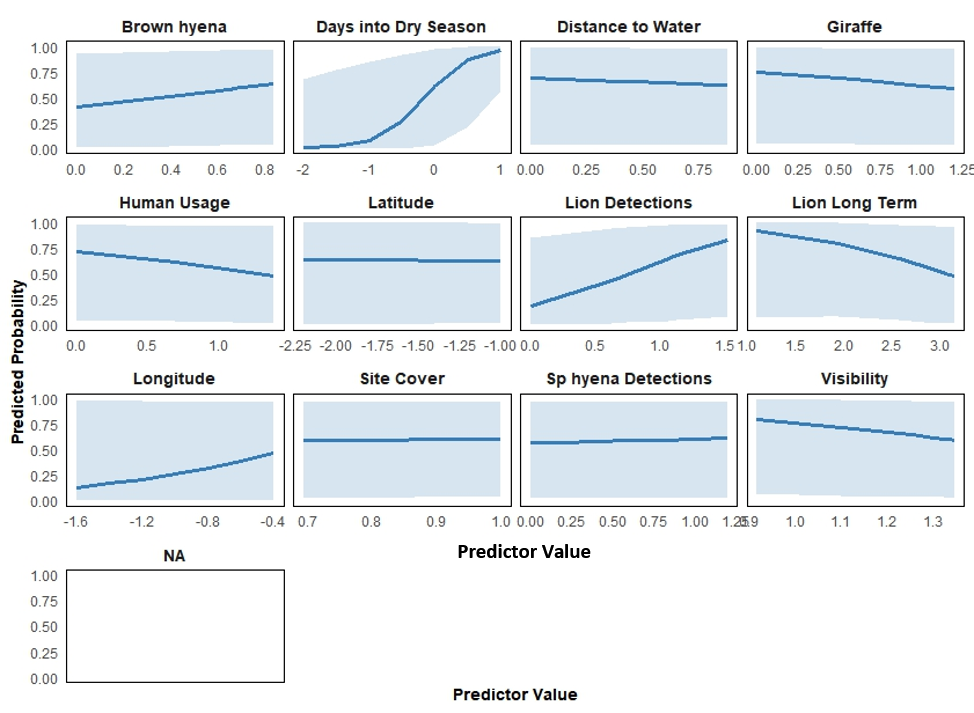


Figure S40. Predicted detection probability of black rhinoceros (*Diceros bicornis*) by land type (government vs. communal concession) based on Bayesian posthoc model outputs derived from camera trap surveys in northwest Namibia.


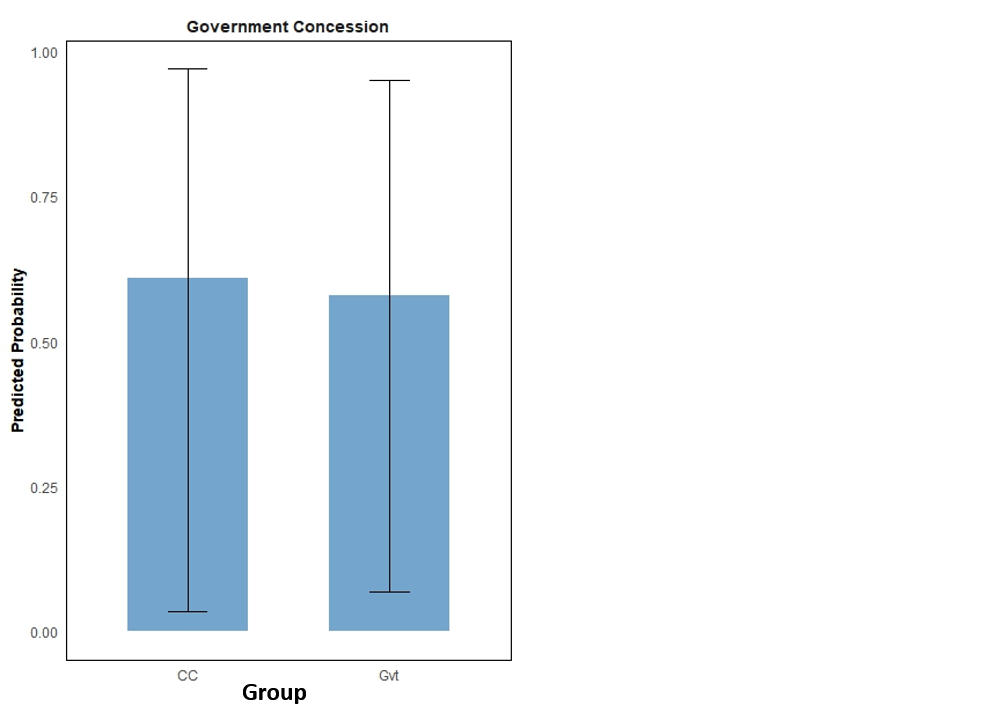


Figure S41. Interaction plots showing predicted detection probability of black rhinoceros (*Diceros bicornis*) in response to interactions between (top left) dry season progression × lion (*Panethera leo*) long-term activity, (top center) dry season progression × spotted hyena (*Crocuta crocuta*) detections, (top right) camera cluster vegetation cover × lion long-term activity, (bottom left) camera cluster visibility × lion detections, and (bottom center) camera cluster visibility × lion long-term activity. Lines show mean predicted values from the Bayesian posthoc model, with shaded ribbons showing 95% credible intervals, based on camera trap data from northwest Namibia.


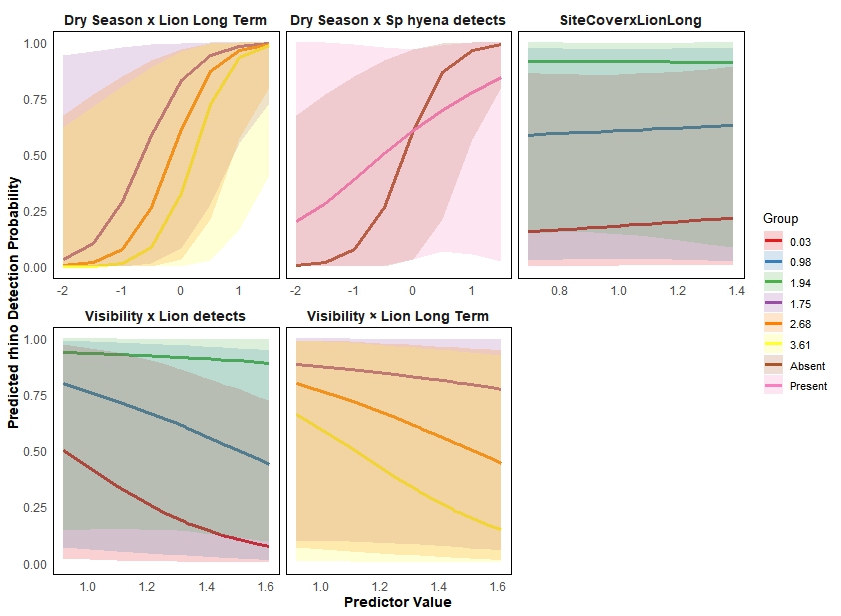


Figure S42. Comparison between predicted detection probabilities from the top Bayesian posthoc model and model-averaged predictions across the candidate model set for black rhinoceros (*Diceros bicornis*), indicating strong agreement and model robustness, derived from camera trap surveys in northwest Namibia.


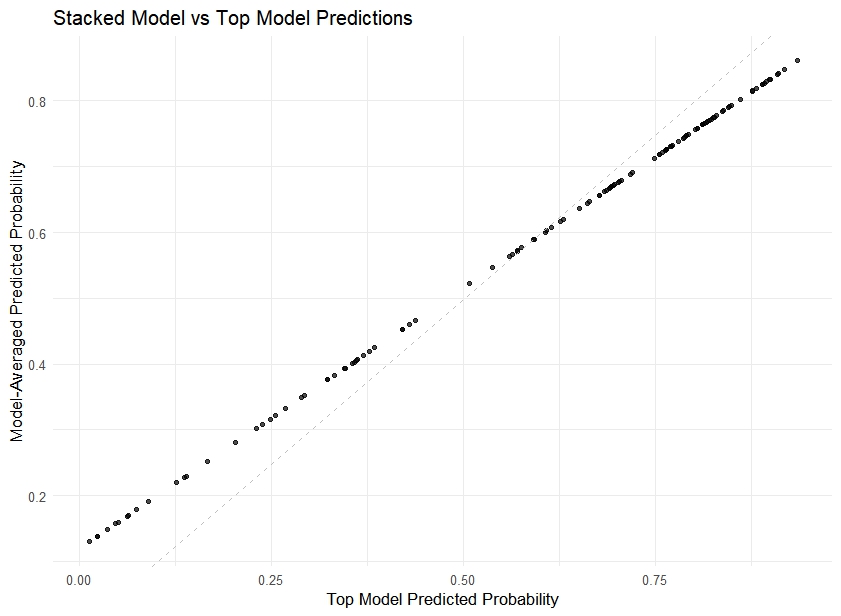


Figure S43. Posterior predictive check for the Bayesian posthoc model of black rhinoceros (*Diceros bicornis*) detection, showing strong alignment between simulated (y_rep) and observed (y) values, indicating good model fit; derived from camera trap surveys in northwest Namibia.


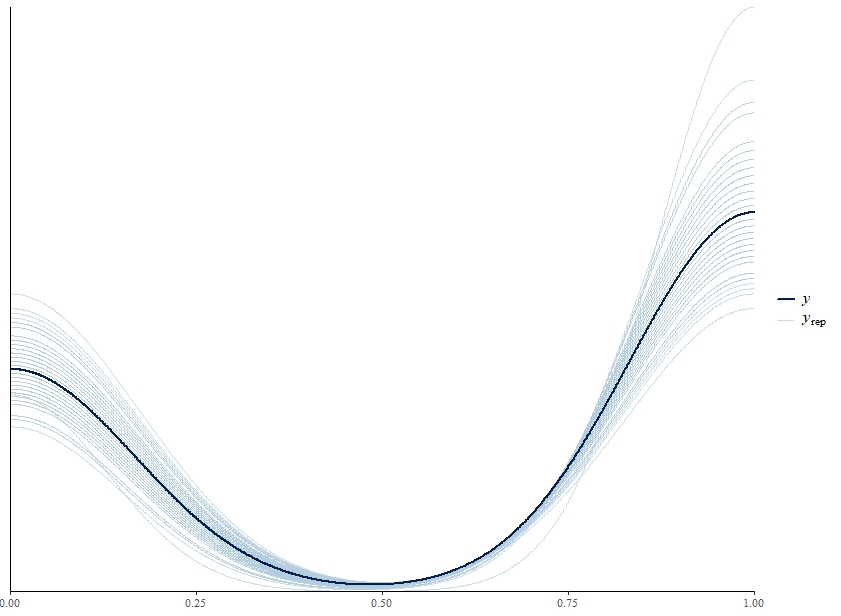


**ELEPHANT**

Table S10. Posterior summaries from the posthoc Bayesian regression model with weak priors evaluating covariates influencing African bush elephant (*Loxodonta africana*) detection probability at camera clusters. Continuous variables log-transformed using log1p to reduce skew. Spatial and temporal predictors were standardized (z-score scaled). Columns include posterior mean estimates, standard errors and 95% credible intervals. Parameters that do not overlap zero are interpreted as having strong evidence of effect.

| **Parameter** | **Estimate** | **Est. Error** | **CrI (2.5%)** | **CrI (97.5%)** |
| --- | --- | --- | --- | --- |
| Intercept | 1.277 | 5.433 | -9.399 | 12.040 |
| Site cover | 0.197 | 1.882 | -3.482 | 3.891 |
| Dist to water | -1.232 | 0.763 | -2.787 | 0.245 |
| Latitude | 2.625 | 1.502 | -0.399 | 5.492 |
| Longitude | 2.021 | 1.252 | -0.514 | 4.472 |
| Dry season | 0.544 | 1.865 | -3.094 | 4.209 |
| Concession/Cons | -0.101 | 1.901 | -3.795 | 3.596 |
| Eland | 0.714 | 0.426 | -0.111 | 1.568 |
| Lion detects | -0.406 | 1.336 | -3.018 | 2.235 |
| Sp hyena detects | -0.364 | 1.381 | -3.082 | 2.358 |
| Lion long term | -0.133 | 1.644 | -3.341 | 3.133 |
| Site cover x Sp hyena detects | 0.871 | 1.280 | -1.637 | 3.391 |
| Site cover x Lion detects | 0.337 | 1.215 | -2.046 | 2.747 |
| Site cover x Lion long term | -0.261 | 0.756 | -1.737 | 1.215 |
| Dry season x Sp hyena detects | -1.015 | 0.717 | -2.423 | 0.393 |
| Dry season x Lion long term | 2.094 | 0.678 | 0.772 | 3.446 |
| Concession/Cons x Sp hyena detects | 0.261 | 0.954 | -1.607 | 2.103 |
| Concession/Cons x Lion detects | 1.157 | 0.657 | -0.149 | 2.451 |
| Concession/Cons x Lion long term | -0.719 | 0.657 | -2.011 | 0.566 |
|  |  |  |  |  |
| **Random Effects** (Intercept) | | |  |  |
| Camera Cluster | 0.75 | 0.31 | 0.14 | 1.38 |
| Survey | 4.03 | 1.4 | 1.91 | 7.35 |

**ELEPHANT BAYESIAN POSTHOC PLOTS**

Figure S44. Marginal effects plots from the Bayesian posthoc model showing predicted detection probability of elephant (*Loxodonta africana*) across continuous covariates at camera traps in northwest Namibia. Species and environmental predictors include dry season progression, eland (*Taurotragus oryx*) detections, latitude, lion (*Panthera leo*) detections, lion long-term activity, longitude, camera cluster vegetation cover, and spotted hyena (*Crocuta crocuta*) detections. Shaded ribbons represent 95% credible intervals.


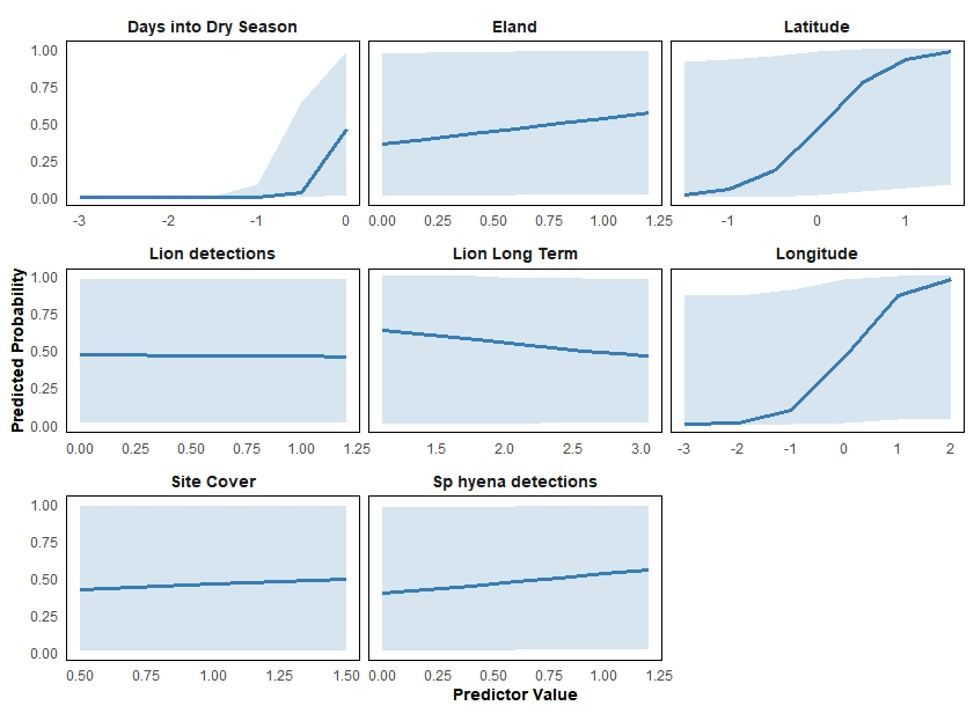


Figure S45. Marginal effects of land management on elephant (*Loxodonta africana*) detection probability within Bayesian posthoc model, derived from camera traps in northwest Namibia. Predicted values and 95% credible intervals are shown for communal conservancies (CC) and government-managed areas (Gvt), averaged across other covariates.


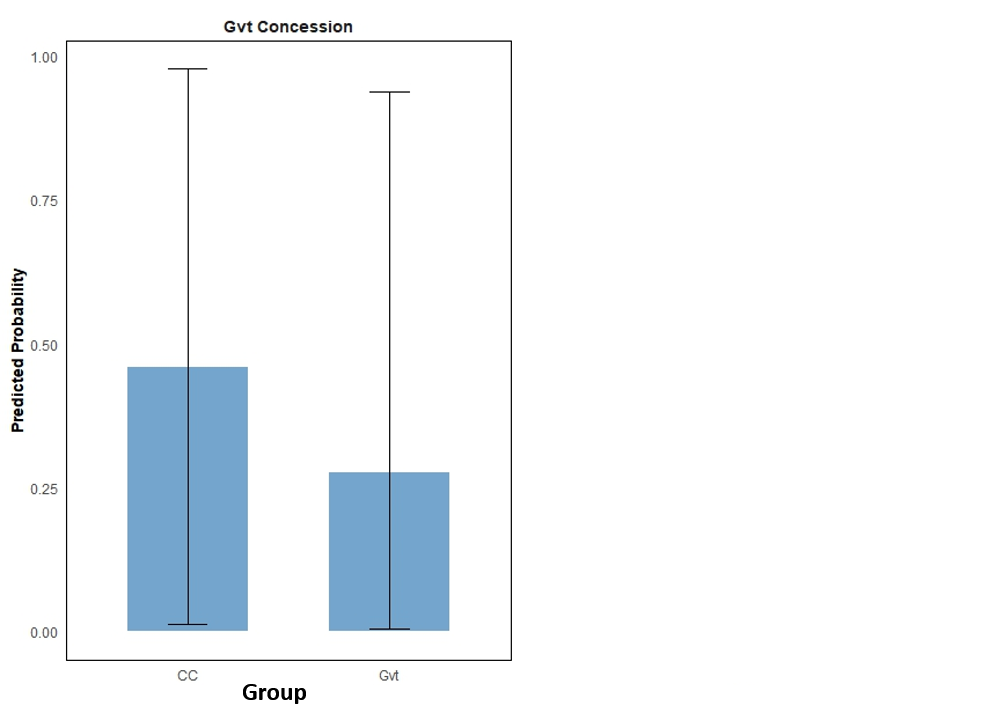


Figure S46. Interaction plots showing predicted detection probability of elephant (*Loxodonta africana*) in response to interactions between (top left) dry season progression × spotted hyena (*Crocuta crocuta*) detections, (top right) camera cluster vegetation cover × lion (*Panthera leo*) detections, (bottom left) camera cluster vegetation cover × lion long-term activity, and (bottom right) camera cluster vegetation cover × spotted hyena detections. Lines show mean predicted values from the Bayesian posthoc model, with shaded ribbons showing 95% credible intervals, based on camera trap data from northwest Namibia.


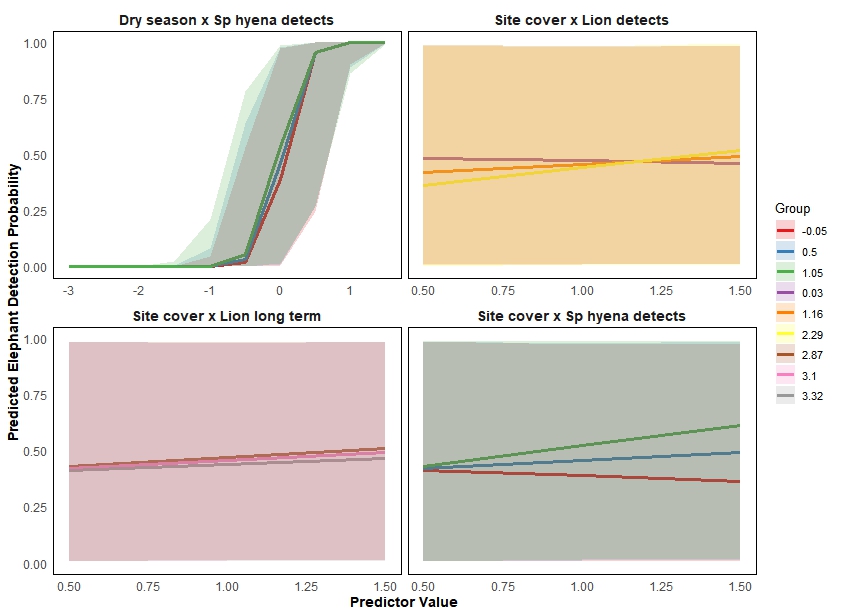


Figure S47. Predicted interaction between dry season depth and lion (*Panthera leo*) long-term presence on elephant (*Loxodonta africana*) detection probability from Bayesian posthoc model, derived from camera traps in northwest Namibia. Detection probability increases during the dry season with greater lion long-term use. Line colors represent varying values of lion long-term presence, with shaded ribbons indicating 95% credible intervals.


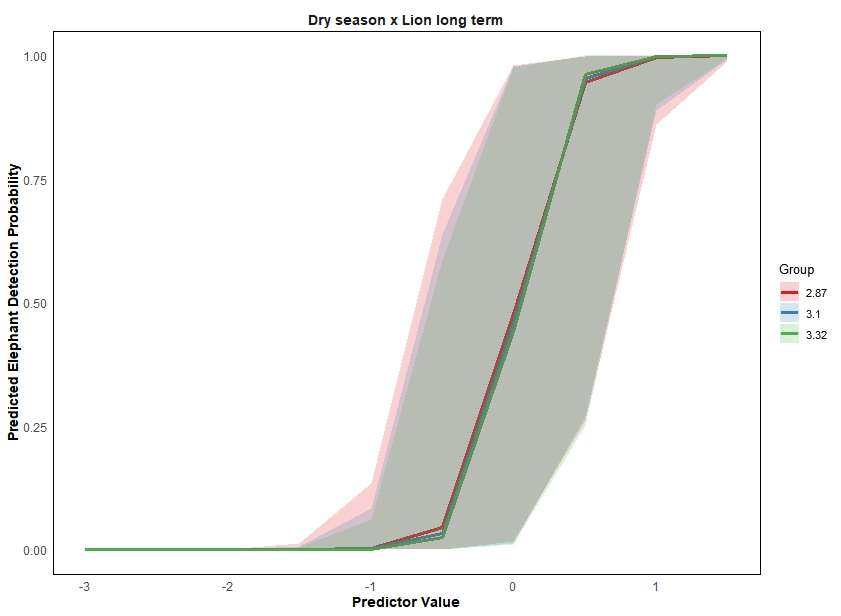


Figure S48. Interaction plots for top continuous × binary predictors from the final Bayesian posthoc elephant (*Loxodonta africana*) detection model, derived from camera traps in northwest Namibia. Each panel shows predicted detection probability of elephants across a continuous predictor gradient, with shaded ribbons representing 95% credible intervals. Group colors represent different levels of the binary variable (e.g., government-managed vs. communal conservancies or predator presence/absence). Panels indicate interactions between (left) government management × lion (*Panthera leo*) detections, (right) government management × lion long-term activity, (left) government management × spotted hyena (*Crocuta crocuta*) detections.


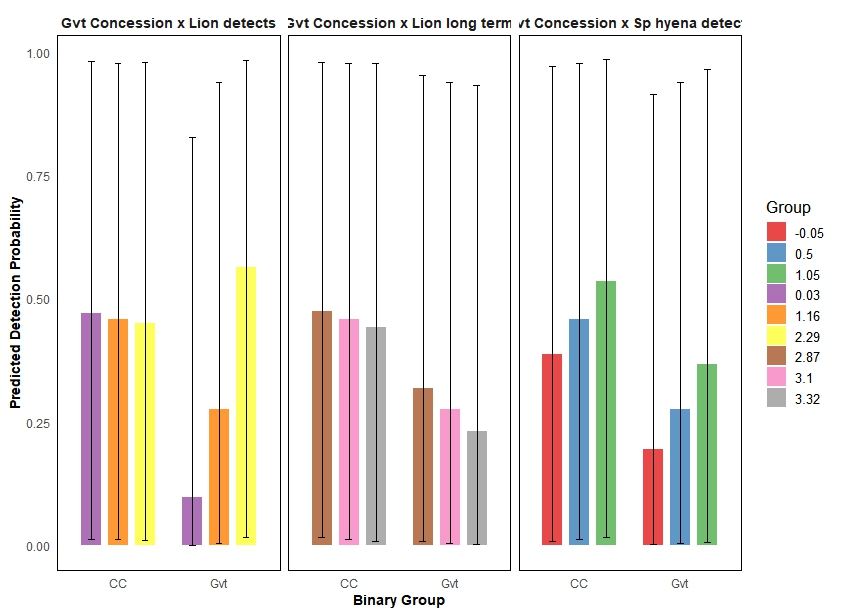


Figure S49. Model-averaged predicted detection probabilities (y-axis) versus predictions from the top individual Bayesian posthoc model (x-axis) for elephant (*Loxodonta africana*) detections, derived from camera traps in northwest Namibia.


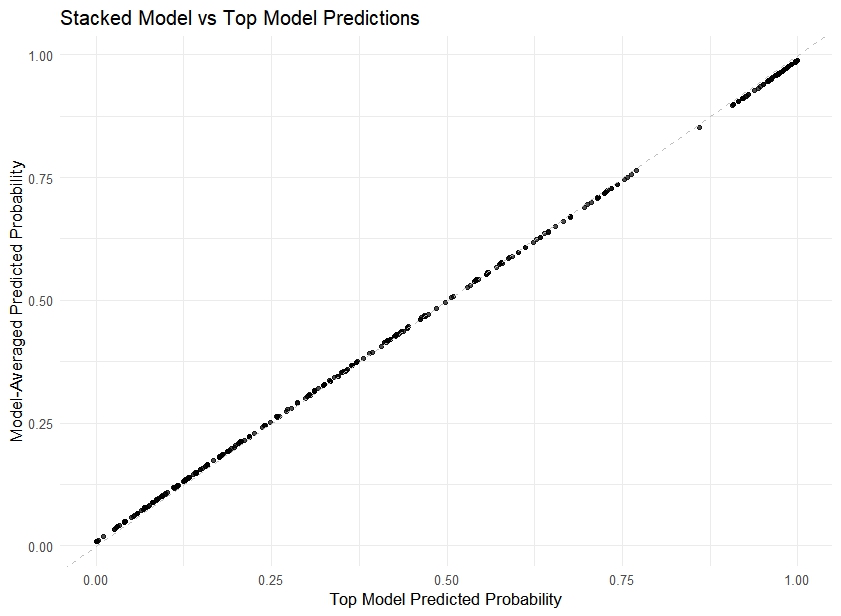


Figure S50. Posterior predictive check comparing model-predicted detection probabilities (light blue curves, *y_rep*) against observed data (*y*, dark line) for Bayesian posthoc model for elephant (*Loxodonta africana*) detections, derived from camera traps in northwest Namibia.


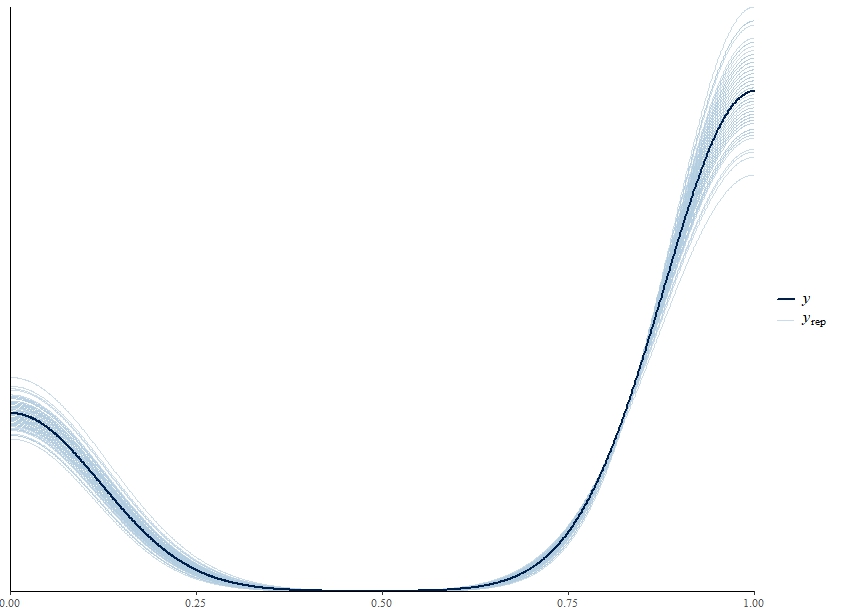


Table S11. Table of results for diel activity pattern overlap estimate (kernel density) of focal herbivores (gemsbok *Oryx gazella*, southern giraffe *Giraffa giraffa*, kudu *Tragelaphus strepsiceros*, Hartmann’s mountain zebra *Equus zebra*, springbok *Antidorcas marsupialis*, black rhinoceros *Diceros bicornis*, African bush elephant *Loxodonta africana*) and apex predators (lion *Panthera leo*, spotted hyena *Crocuta crocuta*) in northwest Namibia. Columns show focal herbivore species; predators; delta (Δ) overlap estimate from 0 (no overlap) to 1 (complete overlap); 95% confidence interval; n, number of independent focal herbivore detections at shared camera clusters; n, number of independent focal carnivore detections at shared camera clusters; estimator (Δ^1^, Δ^4^) used for kernel density.

| **Herbivore-Predator Diel Overlap Estimates** | | | | | | |
| --- | --- | --- | --- | --- | --- | --- |
| Herbivore | Predator | Δ | 95% CI | n herbivore | n predator | Estimator |
| Gemsbok | Lion | 0.185 | 0.14–0.279 | 152 | 115 | Δ^4^ |
|  | Spotted hyena | 0.161 | 0.137–0.244 | 224 | 129 | Δ^4^ |
| Giraffe | Lion | 0.263 | 0.23–0.35 | 357 | 176 | Δ^4^ |
|  | Spotted hyena | 0.119 | 0.114–0.204 | 349 | 128 | Δ^4^ |
| Kudu | Lion | 0.152 | 0.14–0.273 | 164 | 100 | Δ^4^ |
|  | Spotted hyena | 0.061 | 0.055–0.154 | 145 | 61 | Δ^1^ |
| Mountain zebra | Lion | 0.205 | 0.186–0.283 | 668 | 186 | Δ^4^ |
|  | Spotted hyena | 0.172 | 0.16–0.233 | 945 | 183 | Δ^4^ |
| Springbok | Lion | 0.152 | 0.131–0.233 | 388 | 149 | Δ^4^ |
|  | Spotted hyena | 0.111 | 0.1–0.181 | 538 | 143 | Δ^4^ |
| Black rhino | Lion | 0.78 | 0.673–0.862 | 67 | 117 | Δ^1^ |
|  | Spotted hyena | 0.708 | 0.607–0.812 | 103 | 128 | Δ^4^ |
| Elephant | Lion | 0.471 | 0.434–0.582 | 249 | 201 | Δ^4^ |
|  | Spotted hyena | 0.425 | 0.372–0.511 | 230 | 110 | Δ^4^ |
